# The nonreceptor tyrosine kinase SRMS inhibits autophagy and promotes tumor growth by phosphorylating the scaffolding protein FKBP51

**DOI:** 10.1371/journal.pbio.3001281

**Published:** 2021-06-02

**Authors:** Jung Mi Park, Seung Wook Yang, Wei Zhuang, Asim K. Bera, Yan Liu, Deepak Gurbani, Sergei J. von Hoyningen-Huene, Sadie Miki Sakurada, Haiyun Gan, Shondra M. Pruett-Miller, Kenneth D. Westover, Malia B. Potts

**Affiliations:** 1 Department of Cell and Molecular Biology, St. Jude Children’s Research Hospital, Memphis, Tennessee, United States of America; 2 Department of Oncology Research, Amgen Research, Thousand Oaks, California, United States of America; 3 Departments of Biochemistry and Radiation Oncology, The University of Texas Southwestern Medical Center, Dallas, Texas, United States of America; Institute of Cancer Research, Chester Beatty Laboratories, London, UNITED KINGDOM

## Abstract

Nutrient-responsive protein kinases control the balance between anabolic growth and catabolic processes such as autophagy. Aberrant regulation of these kinases is a major cause of human disease. We report here that the vertebrate nonreceptor tyrosine kinase Src-related kinase lacking C-terminal regulatory tyrosine and N-terminal myristylation sites (SRMS) inhibits autophagy and promotes growth in a nutrient-responsive manner. Under nutrient-replete conditions, SRMS phosphorylates the PHLPP scaffold FK506-binding protein 51 (FKBP51), disrupts the FKBP51-PHLPP complex, and promotes FKBP51 degradation through the ubiquitin-proteasome pathway. This prevents PHLPP-mediated dephosphorylation of AKT, causing sustained AKT activation that promotes growth and inhibits autophagy. SRMS is amplified and overexpressed in human cancers where it drives unrestrained AKT signaling in a kinase-dependent manner. SRMS kinase inhibition activates autophagy, inhibits cancer growth, and can be accomplished using the FDA-approved tyrosine kinase inhibitor ibrutinib. This illuminates SRMS as a targetable vulnerability in human cancers and as a new target for pharmacological induction of autophagy in vertebrates.

## Abstract 2

This study describes the discovery and characterization of a nutrient-sensitive signaling pathway that drives growth and inhibits autophagy in mammalian cells. This pathway, which involves the non-receptor tyrosine kinase SRMS and the PHLPP scaffold protein FKBP51, promotes tumor growth and is amenable to pharmacological inhibition.

## Introduction

Evolutionarily ancient nutrient-sensitive kinases such as mTORC1 and AMPK control the metabolic switch between cell growth (when conditions are favorable) and adaptive survival (under times of scarcity) [1]. This is accomplished via opposing regulation of biosynthetic metabolism versus energy-yielding processes such as autophagy [2]. Powerful pharmacological agents including rapamycin, metformin, and their derivatives modulate the activity of this functional class of nutrient-sensitive metabolic regulators for therapeutic benefit in diverse disease settings [1]. Here, we describe an evolutionarily recent addition to this functional class: the vertebrate nonreceptor tyrosine kinase Src-related kinase lacking C-terminal regulatory tyrosine and N-terminal myristylation sites (SRMS) [3].

Nonreceptor tyrosine kinases phosphorylate various substrates to regulate significant molecular pathways in mammalian development and cell growth [4]. SRMS is a nonreceptor tyrosine kinase belonging to the breast tumor kinase (BRK) and Fyn-related kinase (FRK) family [5,6]. SRMS comprises an Src-homology 3 (SH3) and an Src-homology 2 (SH2) domain as well as a kinase domain. Key conserved residues include an ATP-contacting lysine (K258) and an autophosphorylation site in the activation loop (Y380), which are both essential for the enzymatic activation of SRMS [7]. Murine SRMS is located on chromosome 2 and is expressed in various organs and at multiple developmental stages [3]. Human SRMS is located on chromosome 20q13.33, which is adjacent to BRK on the same locus. Interestingly, SRMS is overexpressed in breast cancer cells relative to nontumorigenic breast epithelial cells [7].

We previously identified the nonreceptor tyrosine kinase SRMS as a negative regulator of autophagy [8]. SRMS overexpression inhibits autophagy, and SRMS depletion activates autophagy as evidenced by increased formation of LC3-positive puncta accompanied by the lysosome-dependent degradation of LC3 and p62 in an ULK1- and ATG5-dependent manner [8]. SRMS depletion activates autophagy without detectably altering mTORC1 activity, suggesting that SRMS may inhibit autophagy through an mTORC1-independent mechanism [8]. Recent studies have identified DOK1, BRK, Vimentin, and Sam68 as SRMS substrates [7,9,10]. However, the SRMS substrates reported to date do not adequately explain the mechanism through which SRMS inhibits autophagy.

FK506-binding protein 51 (FKBP51, also known as FKBP5) is a stress response protein that scaffolds specific protein–protein interactions [11]. FKBP51 mediates the interaction between AKT and the PHLPP phosphatase for dephosphorylation of AKT at Ser 473, which reduces AKT activity [12,13]. Moreover, the cellular levels of FKBP51 affect sensitivity to chemotherapy by regulation of AKT as a cell survive kinase. Low levels of FKBP51 permit AKT activity and chemoresistance through persistent AKT phosphorylation on Ser 473 [12]. Active AKT inhibits autophagy through phosphorylation of Beclin 1 at Ser 234 and 295, which facilitates cytoskeletal sequestration of Beclin 1 [14,15].

We report here the identification of FKBP51 as a bona fide endogenous substrate of SRMS. We demonstrate that SRMS phosphorylates FKBP51 to inhibit its scaffolding activity and promote its degradation through the ubiquitin-proteasome pathway. Phosphorylation of FKBP51 by SRMS prevents the interaction of AKT with the PHLPP phosphatase, which leads to persistent activation of AKT under nutrient-replete conditions. Therefore, SRMS inhibits autophagy and accelerates tumor growth by direct phosphorylation of FKBP51.

## Results

### SRMS restrains autophagy under nutrient-rich conditions in a kinase-dependent manner

**Fig 1:**
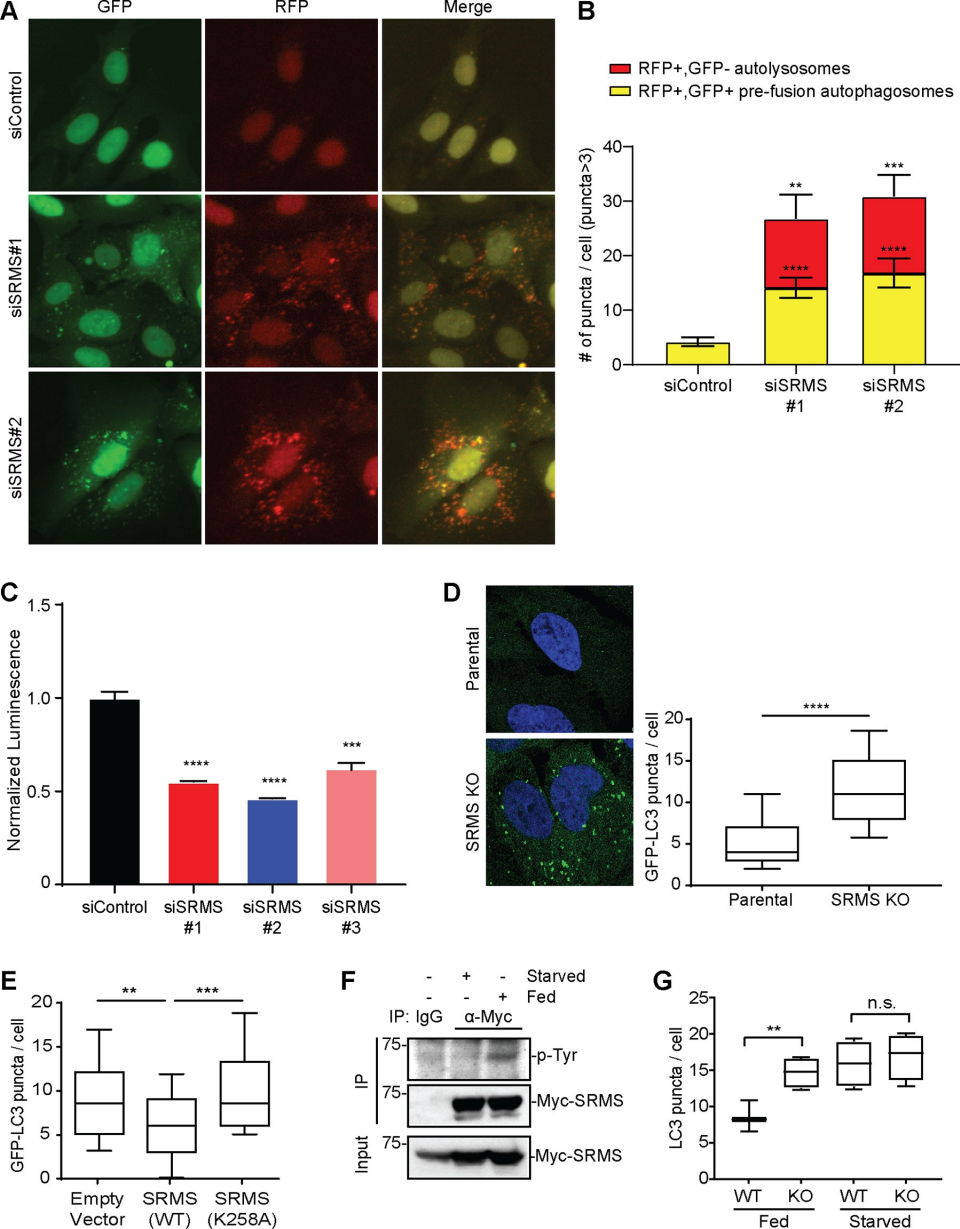
SRMS restrains autophagy under nutrient-replete condition. (**A, B**) Autophagosome–lysosome fusion was assessed in U2OS cells stably expressing tandem monomeric RFP-GFP-LC3. Seventy-two hours after transfection with the indicated siRNA oligonucleotides cells were imaged by IncuCyte to identify prefusion autophagic compartments (i.e., RFP+, GFP+ puncta) and postfusion autolysosomes (i.e., RFP+, GFP− puncta). Representative images are shown in (**A**) and quantitation from cells having more than 3 puncta is shown in (**B**). Mean + SEM from *n =* 18 cells per condition is shown. **p < 0.01, ***p < 0.001, ****p < 0.0001, *t* test. (**C**) Autophagic flux was assessed using the Autophagy LC3 HiBiT Reporter Assay System 72 hours after transfection with the indicated siRNA oligonucleotides. Degradation of the autophagy substrate HiBiT-LC3 was detected as reduced luminescence indicative of increased autophagic flux in SRMS-depleted cells compared to controls. Mean + SEM of *n =* 3 triplicates is shown. ***p < 0.001, ****p < 0.0001, *t* test. (**D**) Baseline numbers of GFP-LC3-positive puncta per cell were compared in parental vs. SRMS KO U2OS GFP-LC3 cells. From left to right *n* = 24 cells and *n* = 23 cells. Representative images are shown. ****p < 0.0001, *t* test. (**E**) SRMS inhibits LC3 puncta formation in a kinase-dependent manner. Myc-SRMS(WT) and Myc-SRMS(K258A) were transiently expressed in U2OS cells stably expressing GFP-LC3. Cells were fixed and stained with anti-Myc antibody to detect transfected cells. The number of GFP-LC3 puncta per cell from *n =* 50 transfected cells per condition is shown. **p < 0.01, ***p < 0.001, *t* test. (**F**) Autophosphorylation of Myc-SRMS was compared between starved (EBSS, 4 hours) and fed (fresh complete growth media, 4 hours) conditions via anti-Myc IP followed by anti-p-Tyr immunoblot. Results shown are representative of 3 independent experiments. (**G**) The number of LC3-positive puncta per cell was compared between SRMS KO vs. WT U2OS cells under starved and fed conditions. From left to right, *n =* 45, *n* = 38, *n* = 40, and *n* = 36 cells were analyzed. ns *p* > 0.05, **p < 0.01, *t* test. The data underlying the figure can be found in S1 Data. EBSS, Earle’s balanced salt solution; GFP, green fluorescent protein; IP, immunoprecipitation; KO, knockout; n.s., non-significant (*p*>0.05); RFP, red fluorescent protein; siRNA, small interfering RNA; SRMS, Src-related kinase lacking C-terminal regulatory tyrosine and N-terminal myristylation sites; WT, wild-type.

We previously reported that SRMS depletion activates autophagy as evidenced by increased formation of LC3-positive puncta accompanied by the lysosome-dependent degradation of LC3 and p62 in an ULK1- and ATG5-dependent manner [8]. To determine whether SRMS depletion also activates autophagosome–lysosome fusion, we used tandem monomeric RFP-GFP-tagged LC3, which distinguishes prefusion autophagic compartments from mature acidic autolysosomes based on the differential pH sensitivity of GFP versus RFP [16]. We found that SRMS depletion increased the average number of both prefusion autophagosomes (RFP-positive; GFP-positive) and postfusion autolysosomes (RFP-positive; GFP-negative) per cell in U2OS RFP-GFP-LC3 cells (Fig 1A and 1B). We next used the U2OS Autophagy LC3 HiBiT Reporter Assay (Promega, Madison, Wisconsin, USA) to monitor degradation of HiBiT-LC3 through autophagic flux [16]. SRMS depletion decreased luminescence in this assay, which indicates increased autophagic flux (Fig 1C). Thus, depletion of SRMS unleashes not only autophagosomes biogenesis but also productive autophagosome–lysosome fusion and autophagic flux. We next generated SRMS knockout U2OS osteosarcoma cells via CRISPR/Cas9-mediated genome editing as an alternative methodology to RNA interference (RNAi) (S1A Fig). The SRMS knockout cells exhibited increased basal autophagy relative to the parental cells as evidenced by an increase in the average number of LC3-positive puncta per cell (Fig 1D). In addition, we isolated primary mouse embryonic fibroblasts (MEFs) from SRMS knockout [3] and wild-type littermates. We measured autophagic flux by counting LC3-positive puncta in the absence and presence of the vacuolar type H^+^-ATPase inhibitor bafilomycin A1, which inhibits lysosomal degradation of autophagosomes [16]. We found that SRMS knockout MEFs have increased numbers of LC3-positive puncta per cell compared to wild-type cells both at baseline and after treatment with bafilomycin A1 (S1B and S1C Fig). Calculations of autophagic flux indicated that approximately twice as many autophagosomes were generated and turned over per hour in SRMS knockout MEFs than in wild-type MEFs (S1D Fig). In addition, the accumulation of lipidated LC3-II in response to bafilomycin treatment was heightened in SRMS knockout MDA-MB-231 cells relative to parental controls (S1E Fig). Together, these results indicate that genetic disruption or depletion of SRMS induces autophagy, confirming that SRMS is a bona fide negative regulator of autophagy in mammalian cells.

We next tested whether the catalytic lysine of SRMS (K258) is required for inhibition of autophagy by SRMS. Catalytic SRMS mutant (K258A) rendered SRMS enzymatically inactive as evidenced by loss of autophosphorylation (S1F Fig). Overexpression of wild-type SRMS, but not kinase-dead SRMS(K258A), reduced the formation of LC3-positive puncta in U2OS cells stably expressing GFP-LC3 (Fig 1E, S1G Fig). This suggests that SRMS inhibits autophagy in a kinase-dependent manner. We next tested whether SRMS kinase activity is sensitive to nutrient status by monitoring SRMS autophosphorylation as previously described [7]. Using phosphoproteomics, we confirmed that, as was previously reported, Y380 in the activation loop of SRMS is the major site of autophosphorylation (S1H Fig). We observed robust SRMS autophosphorylation under nutrient-replete culture conditions (4 hours in fresh, complete growth media) but not under starvation (4 hours in Earle’s balanced salt solution (EBSS)), which indicates that SRMS is sensitive to one or more nutrients present in complete growth media (Fig 1F). The SRMS knockout U2OS cells exhibited increased numbers of LC3-positive puncta per cell selectively under nutrient-rich culture conditions, whereas no significant difference was observed under the starved condition (Fig 1G). Taken together, these results indicate that SRMS restrains autophagy under nutrient-replete conditions in a kinase-dependent manner.

### Ibrutinib inhibits SRMS kinase activity and increases autophagy

**Fig 2:**
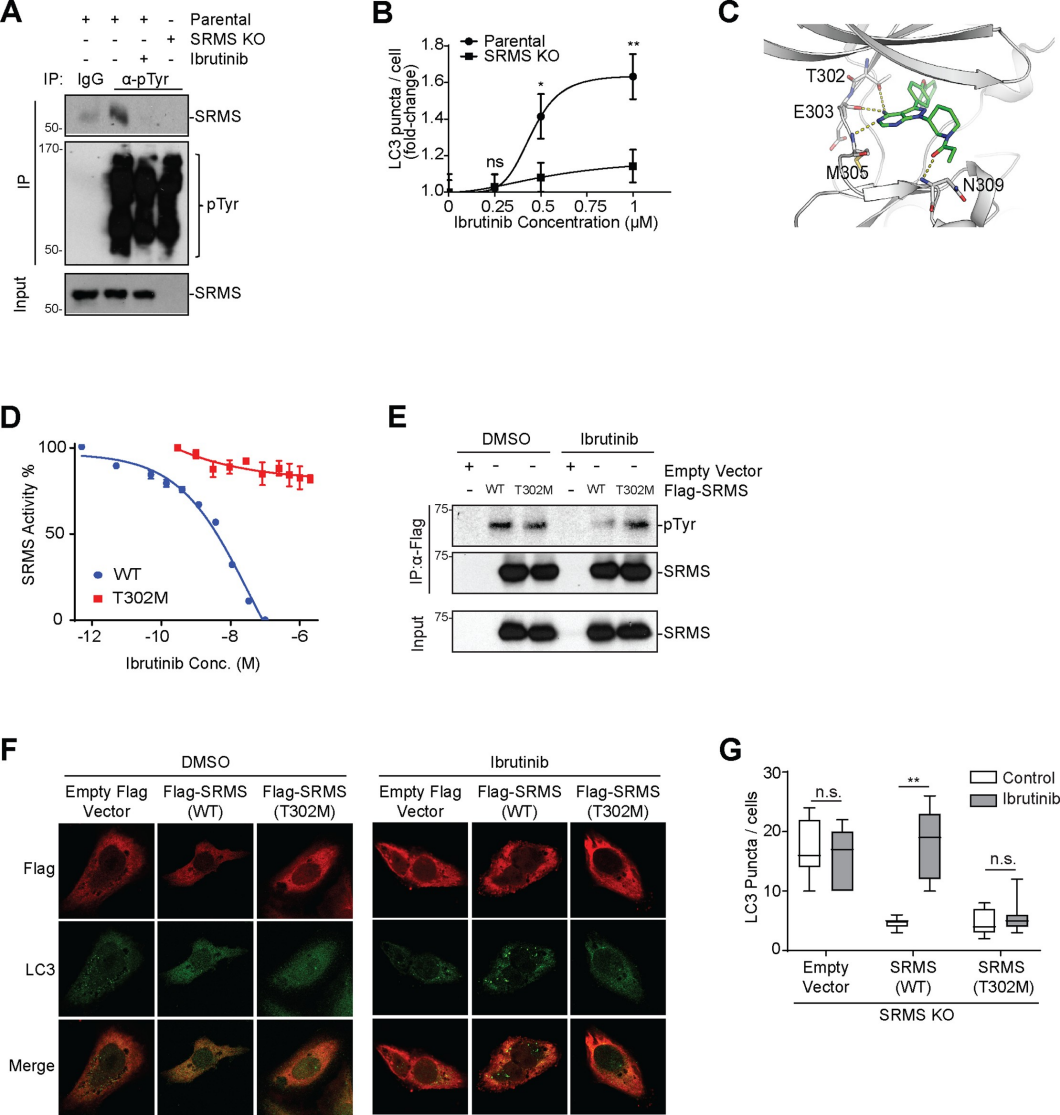
Ibrutinib activates autophagy by direct inhibition of SRMS. (**A**) Ibrutinib inhibits SRMS kinase activity as measured by autophosphorylation. Parental or SRMS KO MDA-MB-231 cells were treated with vehicle or 0.5 μM ibrutinib for 4 hours. Lysates were immunoprecipitated with anti-pTyr antibody and blotted with indicated antibodies. Results shown are representative of 3 independent experiments. (**B**) Ibrutinib induces punctate formation of endogenous LC3 in an SRMS-dependent manner. Parental U2OS cells or SRMS KO U2OS cells were treated with the indicated concentration of ibrutinib for 16 hours. Cells were fixed, stained with anti-LC3 antibody, and imaged. For parental cells, from left to right *n =* 38, *n* = 47, *n* = 49, and *n* = 40 cells were analyzed. For SRMS KO cells, from left to right *n* = 44, *n* = 45, *n* = 58, and *n* = 54 cells were analyzed. The numbers of LC3 puncta per cell were counted and displayed as fold-change vs. the mean value for DMSO-treated cells. Mean +/− SEM is shown. ns *p* > 0.05, *p < 0.05, **p < 0.01, *t* test. (**C**) The SRMS kinase domain (gray) was modeled and unbiased docking of ibrutinib (green) was performed in silico. (**D**) Mobility shift assay was used to measure the enzymatic activity of recombinant purified kinase domains of WT and T302M SRMS in the presence of the indicated concentrations of ibrutinib. (**E**) Ibrutinib inhibits autophosphorylation of WT SRMS but not SRMS(T302M) in cells. Flag-SRMS(WT) or Flag-SRMS(T302M) was expressed in SRMS KO U2OS cells. Cells were treated with DMSO or 0.5 μM ibrutinib for 4 hours. Lysates were immunoprecipitated with anti-Flag antibody and immunoblotted with indicated antibodies. Results shown are representative of 3 independent experiments. (**F, G**) SRMS kinase activity restrains autophagosome biogenesis. SRMS KO U2OS cells expressing empty vector, SRMS(WT), or SRMS(T302M) were treated with 0.5 μM ibrutinib or DMSO (Control) for 4 hours. Cells were stained with anti-LC3 and anti-Flag antibodies followed by confocal microscopy. Representative images are shown in (**F**). The number of LC3-positive puncta per cell from *n =* 25, *n* = 25, *n* = 20, *n* = 32, *n* = 17, and *n* = 20 transfected cells (left to right) are shown in (**G**). ns *p* > 0.05, **p < 0.01, *t* test. The data underlying the figure can be found in S1 Data. IgG, immunoglobulin G; IP, immunoprecipitation; KO, knockout; n.s., non-significant (*p*>0.05); SRMS, Src-related kinase lacking C-terminal regulatory tyrosine and N-terminal myristylation sites; WT, wild-type.

We next asked whether acute pharmacological inhibition of SRMS activates autophagy. The tyrosine kinase inhibitor (TKI) ibrutinib was developed as a covalent inhibitor of bruton tyrosine kinase (BTK) but has also been reported to bind SRMS [17,18]. SRMS does not contain a cysteine residue in the position corresponding to C481 in the original ibrutinib target BTK (N309 in SRMS) and so presumably binds ibrutinib reversibly. To determine whether ibrutinib inhibits SRMS in cells, we first developed an assay in which the activity of overexpressed SRMS can be detected by p-Tyr western blot of whole cell lysates (S2A Fig). In this assay, ibrutinib, but not WP1130 or mubritinib, inhibited Myc-SRMS in a dose-dependent manner (S2B Fig). We next used the human triple-negative breast cancer cell line MDA-MB-231, which robustly expresses endogenous SRMS, to assess the effect of ibrutinib on the activity of endogenous SRMS. Treatment of parental MDA-MB-231 cells with 0.5 μM ibrutinib for 4 hours reduced endogenous SRMS autophosphorylation to a similar extent as genetic knockout of the SRMS locus by CRISPR/Cas9-mediated gene editing (Fig 2A). Notably, bulk tyrosine phosphorylation was largely unaffected by ibrutinib treatment or SRMS gene inactivation (Fig 2A). Ibrutinib activated autophagy in a dose-dependent manner as evidenced by lipidation of LC3-I to form LC3-II, increased formation of LC3-positive puncta, increased formation of acidic autolysosomes, and decreased luminescence in the Autophagy LC3 HiBiT Reporter Assay (Fig 2B, S2C–S2I Fig). The distinct BTK inhibitor acalabrutinib did not activate autophagy, and ibrutinib activated autophagy in parental U2OS cells but not in SRMS knockout U2OS cells, suggesting that ibrutinib activates autophagy by inhibiting SRMS (Fig 2B, S2E, S2G, and S2I Fig).

To confirm this, we generated an ibrutinib-resistant version of SRMS for rescue experiments. We designed the mutant by homology modeling the SRMS kinase domain and using unbiased docking to propose a structural model through which ibrutinib may bind and inhibit SRMS (Fig 2C). This model predicted that T302 of SRMS may be important for ibrutinib binding and that methionine substitution would prevent binding. Therefore, we compared the ability of ibrutinib to inhibit wild-type SRMS versus SRMS(T302M). We found that ibrutinib inhibits wild-type SRMS with an IC50 of 33 nM in vitro, confirming that SRMS is a direct target of ibrutinib (Fig 2D). On the other hand, SRMS(T302M) was enzymatically active but fully resistant to inhibition by ibrutinib in vitro (Fig 2D). To test whether SRMS(T302M) resisted inhibition by ibrutinib in cells, we stably expressed Flag- or Myc-tagged SRMS and SRMS(T302M) in SRMS knockout U2OS cells, treated with ibrutinib, and probed SRMS autophosphorylation by immunoprecipitation (IP) followed by western blot (Fig 2E, S2J Fig). We found that SRMS autoinhibition was sensitive to ibrutinib, whereas SRMS(T302M) autoinhibition was insensitive (Fig 2E, S2J Fig). Together, these results show that ibrutinib directly inhibits SRMS enzymatic activity, whereas SRMS(T302M) is resistant to inhibition by ibrutinib.

Reconstitution of SRMS knockout U2OS cells with either wild-type SRMS or SRMS(T302M) reduced the number of LC3-positive puncta per cell to an equivalent degree (Fig 2F and 2G). This rescue confirms that the elevated autophagy observed in SRMS knockout cells is indeed caused by loss of SRMS. Furthermore, reconstitution of SRMS knockout U2OS cells with wild-type SRMS resensitized to ibrutinib-induced autophagy, whereas reconstitution with the ibrutinib-resistant mutant SRMS(T302M) did not (Fig 2F and 2G). Together, these results indicate that acute pharmacological inhibition of SRMS activates autophagy, which supports the genetic evidence to confirm that SRMS restrains autophagy in a kinase-dependent manner.

### SRMS phosphorylates FKBP51 on tyrosine 54

**Fig 3:**
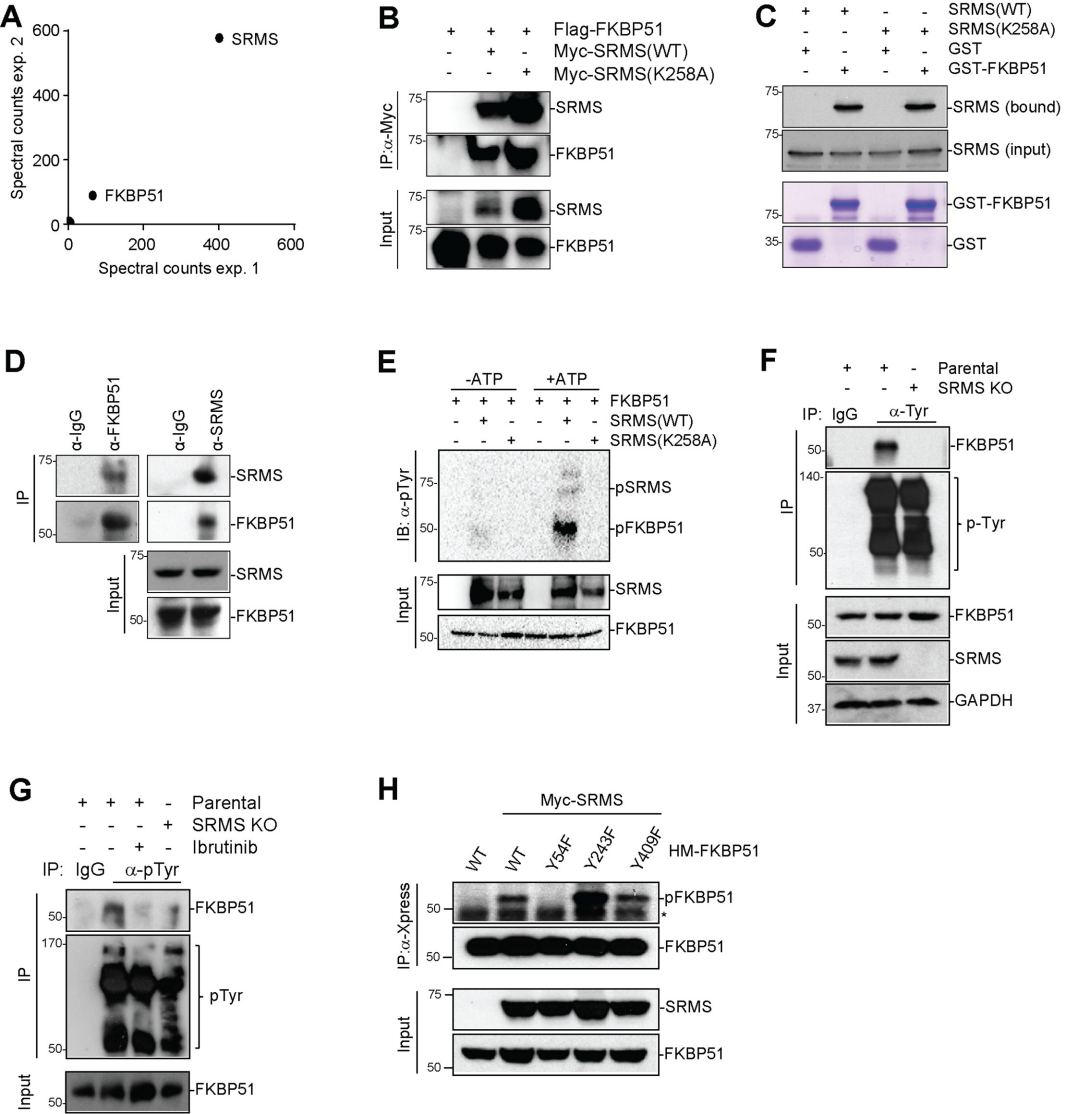
SRMS binds FKBP51 and phosphorylates tyrosine 54. (**A**) Substrate-trapping IP and proteomics were performed to identify candidate SRMS substrates. (**B**) WT and kinase-dead SRMS interact with FKBP51 in cells. Flag-FKBP51 was transfected with Myc-SRMS or Myc-SRMS K258A in HeLa cells. Cell lysates were subjected to IP by anti-Myc antibody and blotted with indicated proteins. Results shown are representative of 3 independent experiments. (**C**) SRMS directly interacts with FKBP51 in vitro. GST pull-down assay was performed with recombinant GST-FKBP51 and in vitro transcribed/translated Myc-SRMS (WT or kinase-dead, as indicated). (**D**) SRMS endogenously interacts with FKBP51. MDA-MB-231 cell lysates were subjected to IP with anti-SRMS or anti-FKBP51 and blotted with indicated antibodies. Results shown are representative of 3 independent experiments. (**E**) In vitro kinase assays were performed using purified recombinant GST-FKBP51 and in vitro transcribed/translated Myc-SRMS(WT) or Myc-SRMS(K258A) proteins in the presence or absence of ATP. Tyrosine phosphorylation of FKBP51 was probed via anti-pTyr antibody and GST-FKBP51 proteins were detected via anti-GST antibody. (**F**) Tyrosine phosphorylation of endogenous FKBP51 and global tyrosine phosphorylation were compared between parental and SRMS KO MDA-MB 231 cells. Results shown are representative of 3 independent experiments. (**G**) Ibrutinib inhibits SRMS kinase activity as measured by phosphorylation of FKBP51. Parental or SRMS KO MDA-MB-231 cells were treated with vehicle or 0.5 μM ibrutinib for 4 hours. Lysates were immunoprecipitated with anti-pTyr antibody and blotted with indicated antibodies. Results shown are representative of 3 independent experiments. (**H**) Myc-SRMS was transfected with each of HisMax-FKBP51 WT, Y54F, Y243F, and Y409F in U2OS cells. Cells were lysed, subjected to IP by anti-Xpress, and blotted with indicated antibodies. *nonspecific band. Results shown are representative of 3 independent experiments. The data underlying the figure can be found in S1 Data. FKBP51, FK506-binding protein 51; GAPDH, glyceraldehyde-3-phosphate dehydrogenase; GST, glutathione S-transferase; IB, immunoblot; IgG, immunoglobulin G; IP, immunoprecipitation; KO, knockout; SRMS, Src-related kinase lacking C-terminal regulatory tyrosine and N-terminal myristylation sites; WT, wild-type.

To identify candidate protein substrates phosphorylated by SRMS, we expressed Flag-SRMS(K258A), Flag-SRMS(WT), or empty Flag vector in HEK293 cells and identified interacting proteins using anti-Flag IP followed by mass spectrometry (Fig 3A, S1 Table). We sought proteins that stably bound to kinase-inactive SRMS(K258A) compared with wild-type SRMS, analogous to the “substrate trapping” strategy that has been useful for detecting substrates of protein tyrosine phosphatases [19]. Fifty-six proteins were identified from Flag-SRMS(K258A) IP but not from Flag-SRMS(WT) or control Flag IPs (S1 Table). The most abundantly and reproducibly detected candidate substrate was the FKBP51 protein encoded by the *FKBP5* gene (Fig 3A). We confirmed that FKBP51 could interact with SRMS by co-IP and found that mutation of the catalytic lysine was not required for detection of the interaction by western blot (Fig 3B). Direct interaction between FKBP51 and SRMS was detected in vitro by a glutathione S-transferase (GST) pulldown assay using bacterially produced GST-FKBP51 and in vitro transcribed/translated SRMS (Fig 3C). We observed physical association between endogenous SRMS and endogenous FKBP51 in MDA-MB-231 cells as evidenced by reciprocal co-IP (Fig 3D). We next mapped the interacting domains of SRMS and FKBP51. Amino acids 213–488 of SRMS, incorporating the kinase domain but lacking the N-terminal, SH3, and SH2 domains, were necessary and sufficient to bind FKBP51 (S3A and S3B Fig). The region of FKBP51 required for SRMS interaction mapped to amino acids 244–301 encompassing the TPR1 domain (S3C–S3E Fig). We found that wild-type SRMS but not kinase-inactive SRMS(K258A) could directly phosphorylate FKBP51 on tyrosine residue(s) in vitro (Fig 3E, S3F Fig). SRMS-dependent tyrosine phosphorylation of FKBP51 could be detected at the endogenous level in MDA-MB-231 cells (Fig 3F). Notably, inactivation of endogenous SRMS by gene editing or ibrutinib treatment specifically abrogated FKBP51 tyrosine phosphorylation without inducing major alterations in global tyrosine phosphorylation patterns (Fig 3F and 3G). Collectively, these results indicate that FKBP51 is a bona fide endogenous substrate of SRMS. Tyrosine phosphorylation of FKBP51 has previously been detected on 3 residues: Y54, Y243, and Y409 [20]. Mutation of FKBP51 tyrosine 54 (but not Y243 or Y409) to phenylalanine abrogated SRMS-mediated FKBP51 tyrosine phosphorylation (Fig 3H). Tyrosine 54 of FKBP51 is well conserved in birds and mammals but is not present in the closely related protein FKBP52 encoded by the *FKBP4* gene (S3G Fig). FKBP51 Y54 is directly adjacent to the V55L variant that has been identified in humans and linked to Paget’s disease of bone [21].

### SRMS disrupts the FKBP51-PHLPP phosphatase to activate AKT

**Fig 4:**
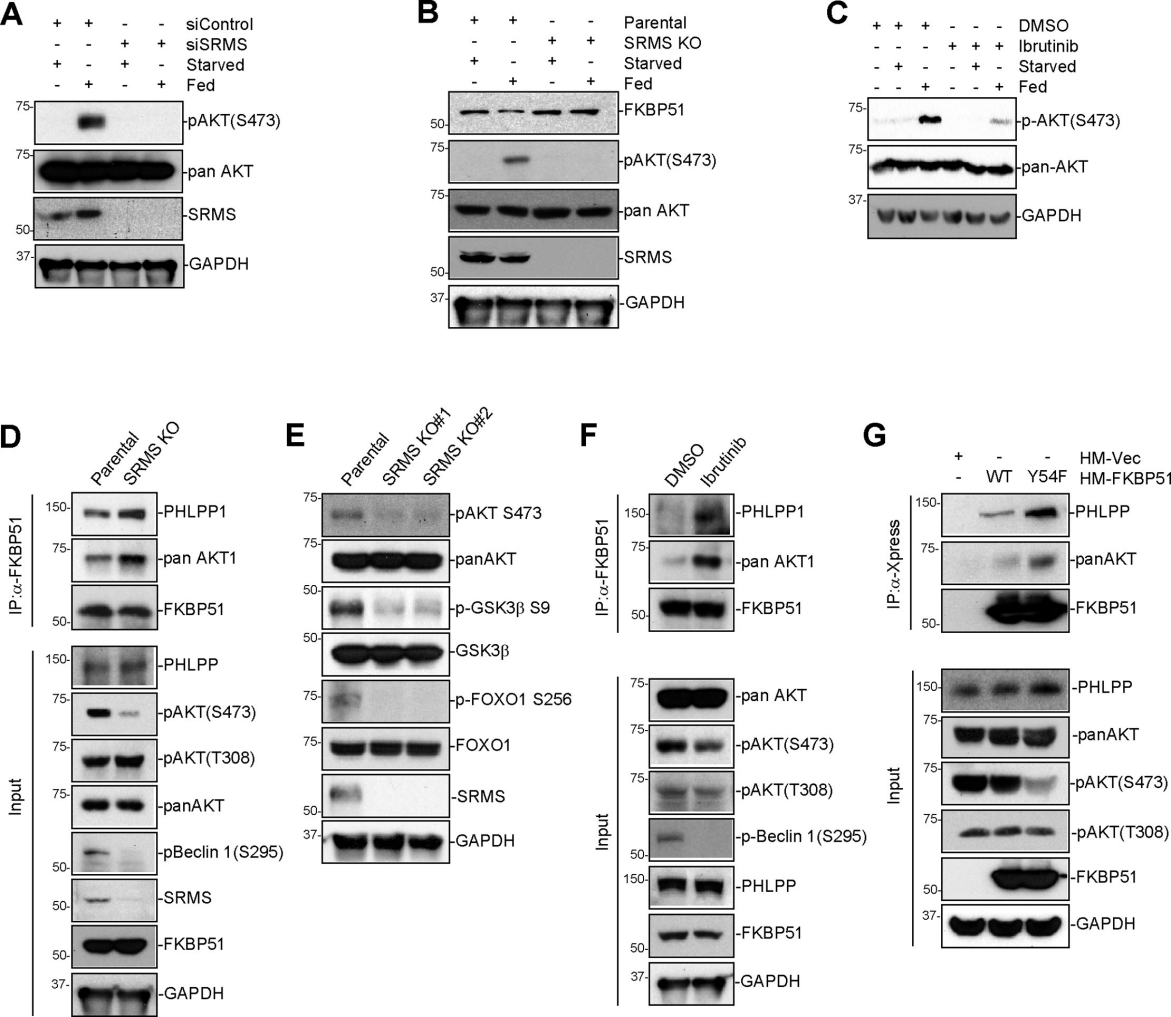
SRMS disrupts the FKBP51-PHLPP-AKT complex to sustain AKT activation. (**A**) SRMS is required for the accumulation of pAKT(S473) under fed conditions. Control or SRMS siRNA was transfected in MDA-MB-231 cells and incubated with starvation or fed condition for 4 hours. Lysates were collected and immunoblotted with the indicated antibodies. (**B**) Nutrient stimulation causes an SRMS-dependent reduction in FKBP51 abundance. Parental or SRMS KO MDA-MB-231 cells were incubated under starved or fed conditions for 4 hours. Lysates were immunoblotted with the indicated antibodies. (**C**) Ibrutinib suppresses the nutrient-dependent accumulation of pAKT(S473). MDA-MB-231 cells were treated with 0.5 μM ibrutinib for 4 hours under starvation or fed condition. Lysates were collected and immunoblotted with the indicated antibodies. (**D**) FKBP51 scaffolding activity was compared between parental and SRMS KO MDA-MB-231 cells. Cells were lysed, subjected to IP by anti-FKBP51, and blotted with indicated antibodies. Results shown are representative of 3 independent experiments. (**E**) AKT signaling pathway activity was compared between parental and SRMS KO MDA-MB-231 cells. Cell lysates were collected and immunoblotted with the indicated antibodies. (**F**) FKBP51 scaffolding activity was compared between MDA-MB-231 cells treated with DMSO vs. 0.5 μM ibrutinib for 4 hours. Cells were lysed, subjected to IP by anti-FKBP51, and blotted with indicated antibodies. Results shown are representative of 3 independent experiments. (**G**) Scaffolding activity of FKBP51(Y54F) vs. WT FKBP51 was compared after transfection in 293FT cells. Cells were lysed, subjected to IP by anti-Xpress, and blotted with indicated antibodies. Results shown are representative of 3 independent experiments. GAPDH, glyceraldehyde-3-phosphate dehydrogenase; IP, immunoprecipitation; KO, knockout; siRNA, small interfering RNA; SRMS, Src-related kinase lacking C-terminal regulatory tyrosine and N-terminal myristylation sites; FKBP51, FK506-binding protein 51; WT, wild-type.

We next asked whether SRMS controls the activity of FKBP51. One reported function of FKBP51 is to inhibit the PI3K-AKT signaling pathway, which itself plays key roles in promoting cell growth and proliferation and inhibiting apoptosis and autophagy [15,22,23]. Specifically, FKBP51 is a scaffold necessary for inactivation of AKT by the PHLPP family of phosphatases via dephosphorylation of p-AKT(S473) [12]. We found that decreasing SRMS by small interfering RNA (siRNA) inhibits phosphorylation of AKT at S473 under nutrient-replete conditions (Fig 4A). We observed the same result when SRMS was knocked out by CRISPR/Cas9-mediated genome editing (Fig 4B). In addition, ibrutinib treatment similarly reduced phosphorylated AKT protein levels (Fig 4C). Defective accumulation of phosphorylated AKT(S473) in SRMS knockout cells was rescued by reintroduction of wild-type but not kinase-inactive SRMS (S4A Fig). Therefore, SRMS kinase activity is required for the activation of AKT in response to nutrient stimulation.

We next observed that overexpression of SRMS reduced the interaction between ectopic FKBP51 and PHLPP1 (S4B Fig). Conversely, elimination of endogenous SRMS by genome editing increased the interaction between FKBP51 and its binding partners PHLPP1 and AKT1 (Fig 4D). This led to decreased phosphorylation of AKT at S473 but not T308 (Fig 4D). Reintroduction of wild-type SRMS normalized FKBP51 scaffolding activity, whereas introduction of kinase-dead SRMS(K258A) failed to do so (S4C Fig). Consistent with the decreased AKT activity in SRMS knockout cells, the downstream AKT substrates Beclin1(S295), GSK3β(S9), and FOXO1(S256) also exhibited decreased phosphorylation in SRMS knockout cells relative to parental cells (Fig 4D and 4E). The interaction between FKBP51 and its binding partners PHLPP and AKT1 similarly increased upon ibrutinib treatment in parental MDA-MB-231 cells (Fig 4F). Also, phosphorylated Beclin1 at S295 protein levels were dramatically decreased by ibrutinib (Fig 4F). Moreover, U2OS cells in which CRISPR-Cas9-mediated genome editing was used to convert the endogenous FKBP51 locus to encode the nonphosphorylation mutant FKBP51(Y54F) show decreased phosphorylation of AKT at Ser 473 but not Thr 308 (S4D Fig). Furthermore, the nonphosphorylatable mutant FKBP51(Y54F) exhibited gain-of-function scaffolding activity relative to wild-type FKBP51 in HEK293 cells (Fig 4G). Together, these data indicate that SRMS inhibits the scaffolding function of its endogenous substrate FKBP51, thus antagonizing FKBP51-dependent inactivation of AKT (S4E Fig).

We also observed an SRMS-dependent reduction in FKBP51 abundance in response to nutrient stimulation (Fig 4B, S4F Fig). Proteasome inhibition by MG132 rescued the nutrient-induced degradation of FKBP51 and enabled detection of poly-ubiquitination of FKBP51 selectively in the fed condition (S4G Fig). FKBP51 poly-ubiquitination could be detected in parental but not SRMS knockout cells (S4H Fig). Proteasome-dependent degradation of the FKBP51 protein under fed conditions was blocked by pharmacological inactivation of SRMS (S4I Fig). Thus, SRMS promotes ubiquitination and proteasome-mediated degradation of FKBP51 under nutrient-replete conditions in addition to inhibiting the scaffolding activity of FKBP51.

### SRMS inhibits Beclin1-dependent autophagy by phosphorylating FKBP51

**Fig 5:**
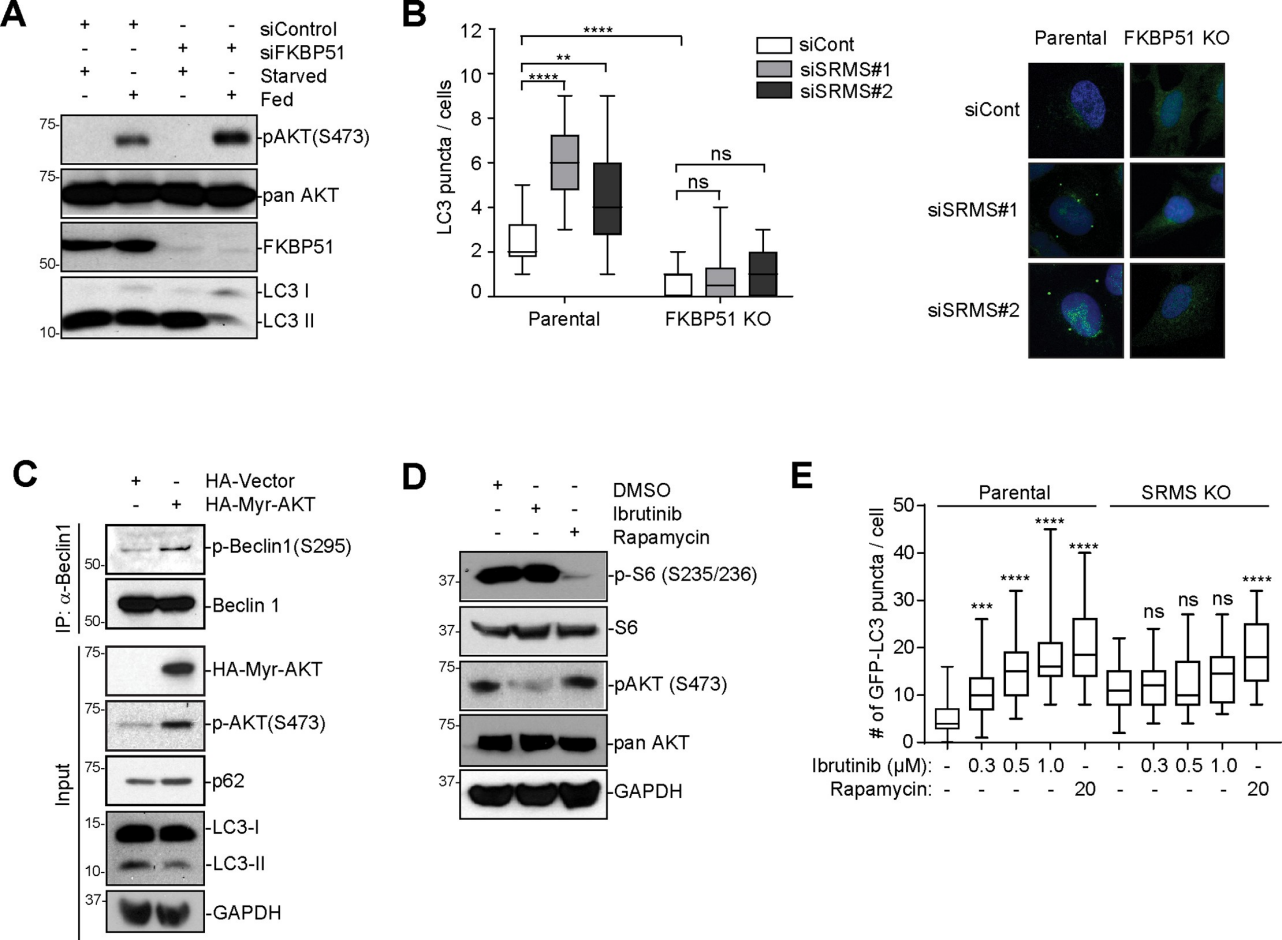
SRMS inhibits autophagy in an FKBP51-dependent manner. (**A**) Depletion of FKBP51 increases p-AKT(S473) and inhibits autophagy in fed conditions. Control siRNA or siRNA targeting FKBP51 was transfected in MDA-MB-231 cells. Seventy-two hours later, cells were fed or starved as indicated for 4 hours. Cell lysates were collected and immunoblotted with indicated antibodies. (**B**) FKBP51 is required for SRMS-mediated regulation of autophagy. SRMS siRNAs were transiently transfected in parental vs. FKBP51 KO U2OS cells. Cells were fixed and stained with anti-LC3 antibody to detect endogenous LC3 puncta. Representative images are shown. The number of puncta per cell from *n =* 18 cells per condition is shown. ns *p* > 0.05, **p < 0.01, ****p < 0.0001, *t* test. (**C**) Constitutively active AKT rescues phosphorylation of Beclin1(Ser295) and inhibits autophagy in SRMS KO cells. Myr-AKT was stably expressed in SRMS KO MDA-MB 231 cells. Beclin 1 IP was performed, and lysates were analyzed by western blot using the indicated antibodies. Results shown are representative of 3 independent experiments. (**D**) Ibrutinib does not inhibit mTORC1. MDA-MB-231 cells were treated with 0.5 μM ibrutinib or 10 nM rapamycin for 4 hours. Lysates were analyzed by western blot with the indicated antibodies. (**E**) Ibrutinib activates autophagosome formation in an SRMS-dependent manner, while rapamycin activates autophagosome formation in an SRMS-independent manner. Parental or SRMS KO U2OS GFP-LC3 cells were treated with vehicle or the indicated concentrations of ibrutinib or rapamycin for 16 hours. Cells were fixed and imaged by confocal microscopy. From left to right, *n* = 24, *n* = 33, *n* = 29, *n* = 31, *n* = 26, *n* = 23, *n* = 31, *n* = 31, *n* = 28, and *n* = 31 cells were analyzed. ns *p* > 0.05, ***p < 0.001, ****p < 0.0001, *t* test. The data underlying the figure can be found in S1 Data. FKBP51, FK506-binding protein 51; GAPDH, glyceraldehyde-3-phosphate dehydrogenase; IP, immunoprecipitation; KO, knockout; siRNA, small interfering RNA; SRMS, Src-related kinase lacking C-terminal regulatory tyrosine and N-terminal myristylation sites.

These mechanistic studies suggest a specific molecular pathway through which SRMS may inhibit autophagy (S4E Fig). Nutrient-replete conditions activate SRMS, which phosphorylates FKBP51 on Y54 inhibiting its function as a PHLPP scaffold and accelerating its degradation. This mechanism prevents inactivation of AKT by the PHLPP phosphatase, which would otherwise dephosphorylate AKT (Ser473). Persistence of active AKT results in robust phosphorylation of the core autophagy protein Beclin 1 on Ser234,295, which is an inhibitory site, thus blocking autophagy at an early step prior to LC3 lipidation [15,22]. We tested this pathway by manipulating FKBP51, AKT, and Beclin 1 and measuring the effects on autophagy. Depletion of FKBP51 resulted in increased accumulation of p-AKT(Ser473) and decreased lipidation of LC3 under nutrient-replete conditions (Fig 5A). Similarly, FKBP51 knockout cells exhibit increased phosphorylation of AKT at Ser473 and decreased basal autophagy as evidenced by a reduction in the number of LC3-positive puncta per cell under nutrient-replete conditions (Fig 5B, S5A Fig). To test whether SRMS restrains autophagy via inhibition of its endogenous substrate FKBP51, we depleted SRMS by RNAi in parental versus FKBP51 knockout cells and measured the effect on autophagosome formation by quantifying the number of LC3-positive puncta per cell (Fig 5B). SRMS depletion activated autophagy in parental cells but failed to activate autophagy in FKBP51 knockout cells (Fig 5B). These results show that FKBP51 is a major substrate through which SRMS inhibits autophagy. Introduction of constitutively active AKT into SRMS knockout cells rescued Beclin 1 (Ser295) phosphorylation and attenuated LC3 lipidation, confirming that AKT acts downstream of SRMS to regulate Beclin 1 phosphorylation and autophagy (Fig 5C). Depletion of Beclin 1 reversed the autophagy induction caused by depletion of SRMS, confirming that Beclin 1 acts downstream of SRMS to regulate autophagy (S5B and S5C Fig). Genetic or pharmacological inhibition of SRMS increased the formation of pro-autophagy Beclin 1 protein complexes as evidenced by increased association of Beclin 1 with pro-autophagy proteins VPS34, UVRAG, and ATG14 but no increase in association with the anti-autophagy protein RUBICON (S5D and S5E Fig). This indicates that SRMS signaling negatively regulates formation of the Beclin 1-Vps34 autophagy complexes. Ibrutinib versus rapamycin selectively inhibited AKT versus mTORC1, respectively, and SRMS knockout cells were resistant to ibrutinib-induced autophagy but sensitive to rapamycin-induced autophagy (Fig 5D and 5E). Collectively, these results indicate that SRMS inhibits autophagy through an FKBP51-AKT-Beclin 1 pathway. Because both SRMS and FKBP51 are evolutionarily restricted to the bony vertebrate lineage, the SRMS-FKBP51 signaling pathway represents a relatively recent evolutionarily innovation in the control of cellular metabolism [24].

### SRMS promotes tumor growth in a kinase-dependent manner

**Fig 6:**
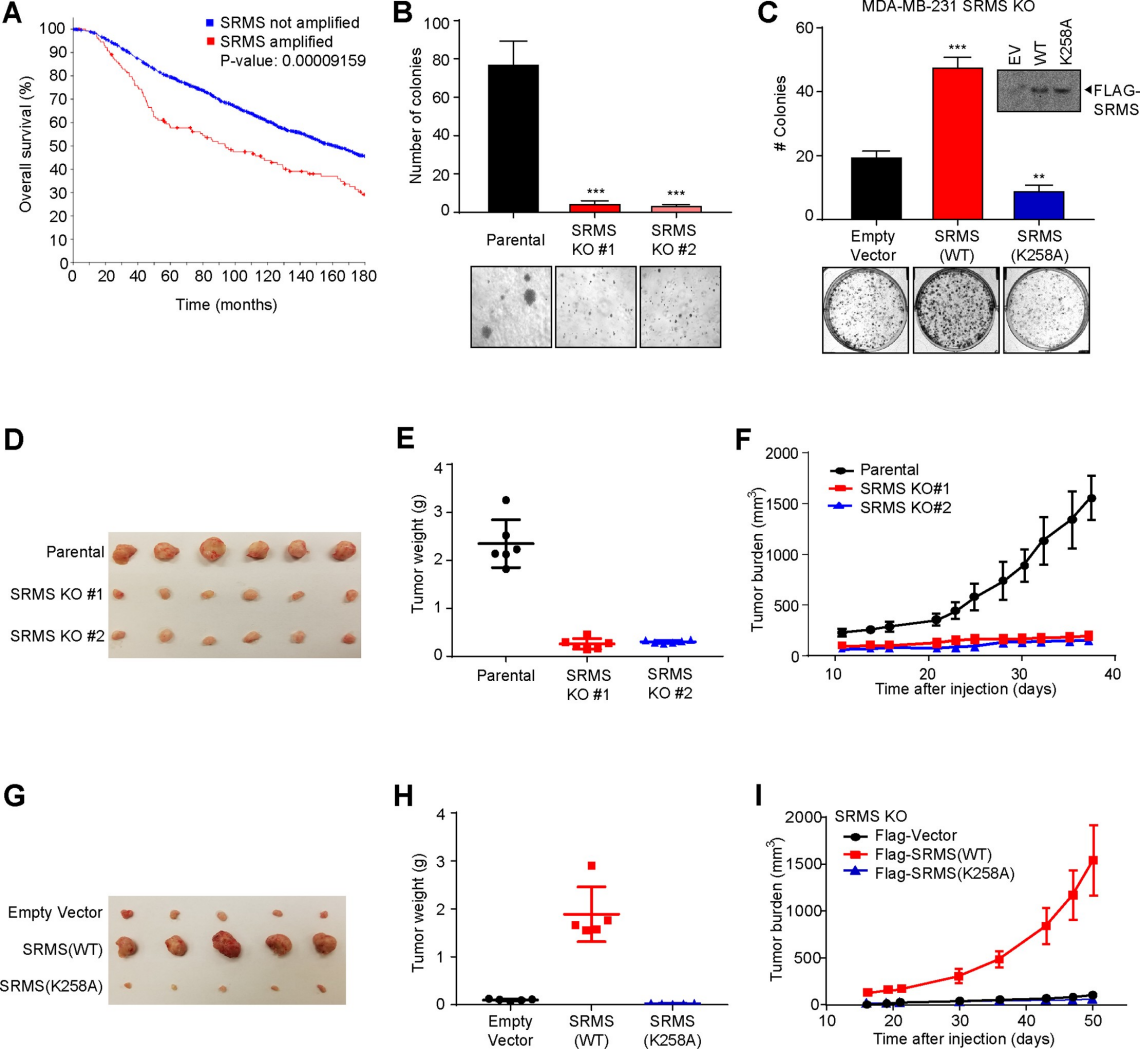
SRMS promotes tumor growth in a kinase-dependent manner. (**A**) SRMS amplification correlates with poor overall survival in breast cancer patients. The METABRIC cohort of 2,173 breast cancer patients were split into SRMS-nonamplified (blue) and SRMS-amplified (red) groups. Overall, survival of each group was plotted. (**B**) SRMS is required for anchorage-independent growth of MDA-MB-231 triple-negative breast cancer cells. Parental or SRMS KO MDA-MB-231 cells were seeded sparsely in soft agar and allowed to grow for 23 days. Mean + standard deviation for *n =* 3 replicates are shown along with representative images. ***p < 0.001. (**C**) SRMS kinase activity supports clonogenic growth of MDA-MB-231 cells. SRMS KO MDA-MB-231 cells were transfected with empty vector, SRMS WT, or SRMS K258A kinase-dead mutant and plated sparsely. After 20 days of clonogenic growth, colonies were stained with crystal violet, imaged, and counted. Mean + standard deviation of *n* = 3 replicates is shown along with representative images. **p < 0.01, ***p < 0.001, *t* test. (Relates to S6D Fig). (**D–F**) Xenograft tumor growth was compared between parental vs. SRMS KO MDA-MB-231 cells injected subcutaneously into Nod-Scid gamma mice. Relative tumor burden (**D**) and tumor weights (**E**) corresponding with the endpoint of the growth curve (**F**) are shown. Mean +/− standard deviation of *n* = 6 mice per condition. (**G–I**) SRMS KO MDA-MB-231 cells were reconstituted with the indicated constructs and injected subcutaneously into Nod-Scid gamma mice. Relative tumor burden (**G**) and tumor weights (**H**) corresponding with the endpoint of the growth curve (**I**) are shown. Mean +/− standard deviation of *n* = 5 mice per condition. The data underlying the figure can be found in S1 Data. EV, empty vector; KO, knockout; SRMS, Src-related kinase lacking C-terminal regulatory tyrosine and N-terminal myristylation sites; WT. wild-type.

Human cancer cells from diverse tissues of origin commonly harbor mutations and copy number variations that drive unrestrained PI3K-AKT signaling through enhanced activation or impaired inactivation of the pathway [15,25]. We used gene expression profiling interactive analysis to compare SRMS expression in human tumors versus corresponding normal tissues [26]. We found that SRMS expression is broadly elevated in human breast, colon, and rectal tumors relative to corresponding normal tissues (S6A Fig). We analyzed data from the METABRIC cohort of 2,173 breast cancer patients and found that approximately 7% (145 patients) exhibited amplification of the SRMS locus within their tumors [27–30]. This suggests that copy number variation is one potential driver of SRMS overexpression in a small subset of patients, although SRMS hypomethylation in metastatic breast cancer has also been reported [31]. Within the METABRIC cohort, overall survival was significantly shorter in patients with SRMS amplifications than in those without (Fig 6A). Median survival in patients with SRMS amplification was 94.0 months versus 160.4 months otherwise, a difference of over 5 years (*p* < 0.0001). The most common oncogenic driver in the METABRIC cohort was activation of the PIK3CA gene by mutation and/or amplification, which occurred in 42% of patients. Co-occurrence between SRMS amplification and PIK3CA activation was significantly less than would be expected by chance (log odds ratio −0.445, *p* = 0.009). These data are consistent with the possibility that SRMS amplification and overexpression may be previously unrecognized drivers of unrestrained PI3K-AKT signaling in human cancers.

To investigate whether SRMS signaling contributes to cancer growth, we inactivated SRMS in human cancer cells and measured the effects on growth in culture and in vivo. SRMS knockout MDA-MB-231 cells were profoundly impaired in anchorage-independent growth (Fig 6B). SRMS knockout MDA-MB-231 and U2OS cells were also profoundly impaired in two-dimensional clonogenic growth (S6B and S6C Fig). Impaired growth of SRMS knockout U2OS and MDA-MB-231 cells was rescued by reintroduction of wild-type SRMS but not enzymatically inactive SRMS(K258A) (Fig 6C, S6D Fig). Moreover, U2OS cells in which endogenous FKBP51 was edited to the nonphosphorylatable mutant FKBP51(Y54F) demonstrated reduced two-dimensional clonogenic growth (S6E Fig). We next asked whether SRMS supports tumor growth in vivo. While parental MDA-MB-231 cells readily formed tumors when injected subcutaneously into NSG recipient mice, SRMS knockout MDA-MB-231 cells failed to do so (Fig 6D–6F). Xenograft tumor growth was rescued in the SRMS knockout cells by reintroduction of wild-type SRMS but not the enzymatically inactive SRMS(K258A) mutant (Fig 6G–6I). Collectively, these results indicate that SRMS kinase activity is necessary for aggressive growth of diverse human cancer cells in culture and in vivo.

### SRMS-AKT signaling inhibits the tumor suppressor Beclin 1

Constitutively active AKT rescued anchorage-independent growth of SRMS knockout MDA-MB-231 cells, indicating that AKT acts downstream of SRMS to promote growth (S6F Fig). To investigate whether phosphorylation of the AKT substrate Beclin 1 contributes to SRMS-dependent tumor growth, we used MCF7 cells, which do not robustly express endogenous Beclin 1 [32]. We introduced wild-type Beclin 1 or a mutant version of Beclin 1 (S234A, S295A) that is resistant to inhibitory phosphorylation by AKT (S6G Fig) [15]. We then measured the effects of SRMS inactivation on anchorage-independent growth. Compared to introduction of wild-type Beclin 1, introduction of Beclin 1 (S234A, S295A) impaired anchorage-independent growth to a similar degree as RNAi-mediated depletion or pharmacological inhibition of SRMS (S6H and S6I Fig). The combination of Beclin 1 (S234A, S295A) expression with SRMS inactivation did not further exacerbate the phenotype relative to either treatment alone (S6H and S6I Fig). These results are consistent with the interpretation that SRMS antagonizes the tumor-suppressive activity of Beclin 1 by promoting AKT-dependent phosphorylation on S234 and/or S295.

### Autophagy inhibition by SRMS prevents widespread cancer cell senescence

**Fig 7:**
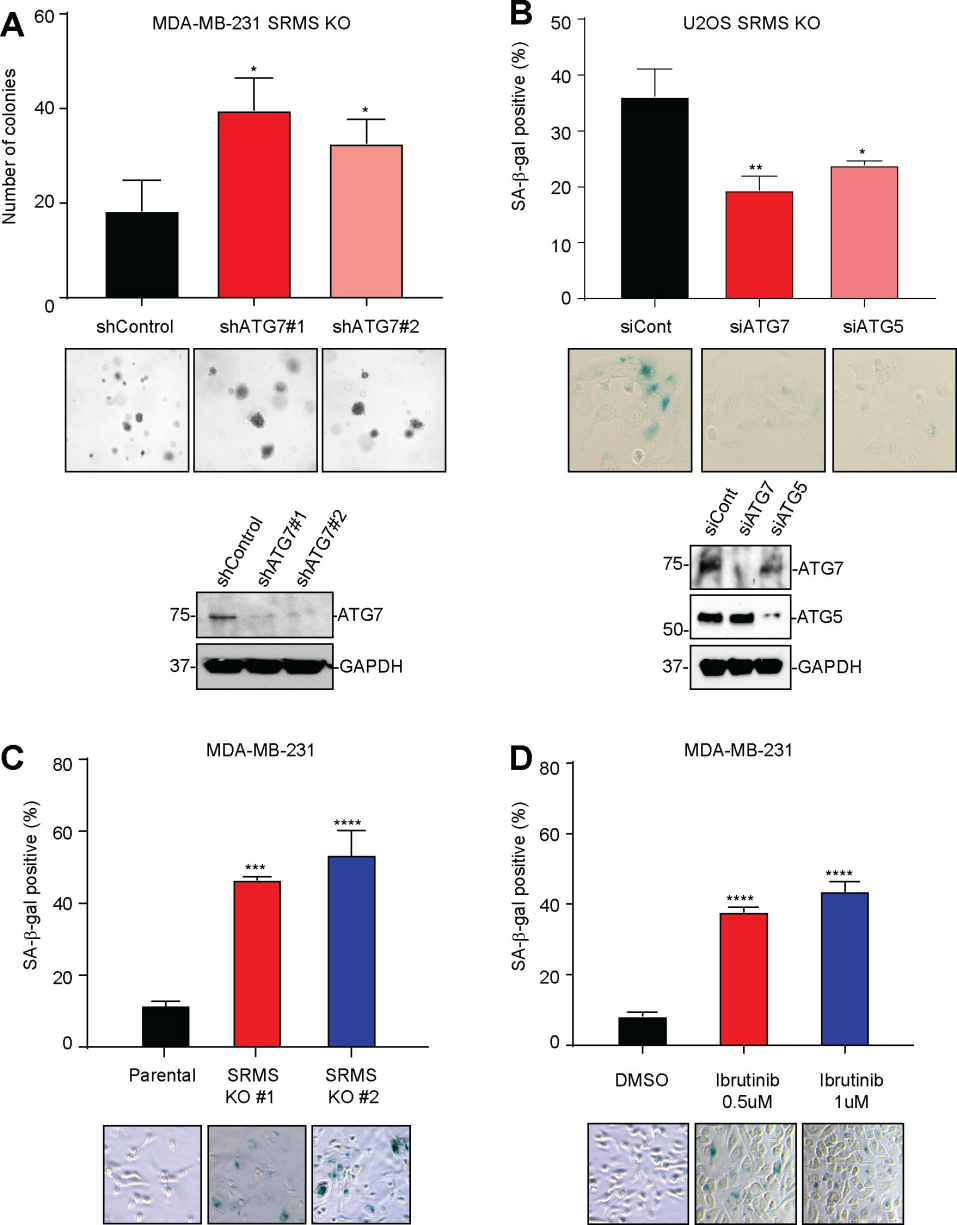
Autophagy inhibition by SRMS prevents widespread cancer cell senescence. (**A**) Soft agar colony formation assay was performed in SRMS KO MDA-MB-231 cells after introduction of the indicated shRNA constructs. Mean + SEM of *n =* 3 replicates are shown (top) along with representative images (middle) and western blot evaluating knockdown efficiency (bottom). (**B**) SRMS KO U2OS cells were transfected with the indicated siRNA oligonucleotides. Seventy-two hours later, senescence-associated beta-galactosidase staining was performed in one set, while lysates were collected from a parallel set of cells. Mean + SEM of *n =* 3 replicates are shown (top) along with representative images (middle) and western blot analysis of knockdown efficiency (bottom). (**C**) Senescence-associated beta-galatosidase staining was performed in parental MDA-MB-231 cells and 2 SRMS KO MDA-MB-231 clones. Mean + SEM of *n =* 3 replicates are shown along with representative images. (**D**) Senescence-associated beta-galactosidase staining was performed in parental MDA-MB-231 cells treated with DMSO or the indicated concentration of ibrutinib. Mean + SEM of *n* = 3 experiments are shown along with representative images. The data underlying the figure can be found in S1 Data. GAPDH, glyceraldehyde-3-phosphate dehydrogenase; KO, knockout; shRNA, short hairpin RNA; siRNA, small interfering RNA; SRMS, Src-related kinase lacking C-terminal regulatory tyrosine and N-terminal myristylation sites.

PI3K-AKT signaling promotes tumor growth through phosphorylation of myriad substrates that control diverse metabolic and cell survival pathways. Whether autophagy inhibition per se plays any role in promotion of tumor growth by SRMS-FKBP51-AKT signaling is an important question. To address this, we measured the growth of human breast cancer cells after depletion of SRMS alone versus co-depletion of SRMS and core autophagy genes. We found that depletion of SRMS impaired short-term two-dimensional growth in an autophagy-independent manner (S7 Fig), whereas depletion of SRMS impaired long-term three-dimensional anchorage-independent growth in a manner that required core autophagy genes (Fig 7A). Mechanistically, genetic or pharmacological inhibition of SRMS induced cancer cell senescence in an ATG5- and ATG7-dependent manner (Fig 7B–7D). These results indicate that the SRMS-FKBP51-PHLPP-AKT signaling axis promotes tumor growth through a combination of autophagy-dependent mechanisms (such as preventing senescence) and autophagy-independent mechanisms (such as increasing short-term two-dimensional growth rate).

### SRMS is a targetable vulnerability in cancer

**Fig 8:**
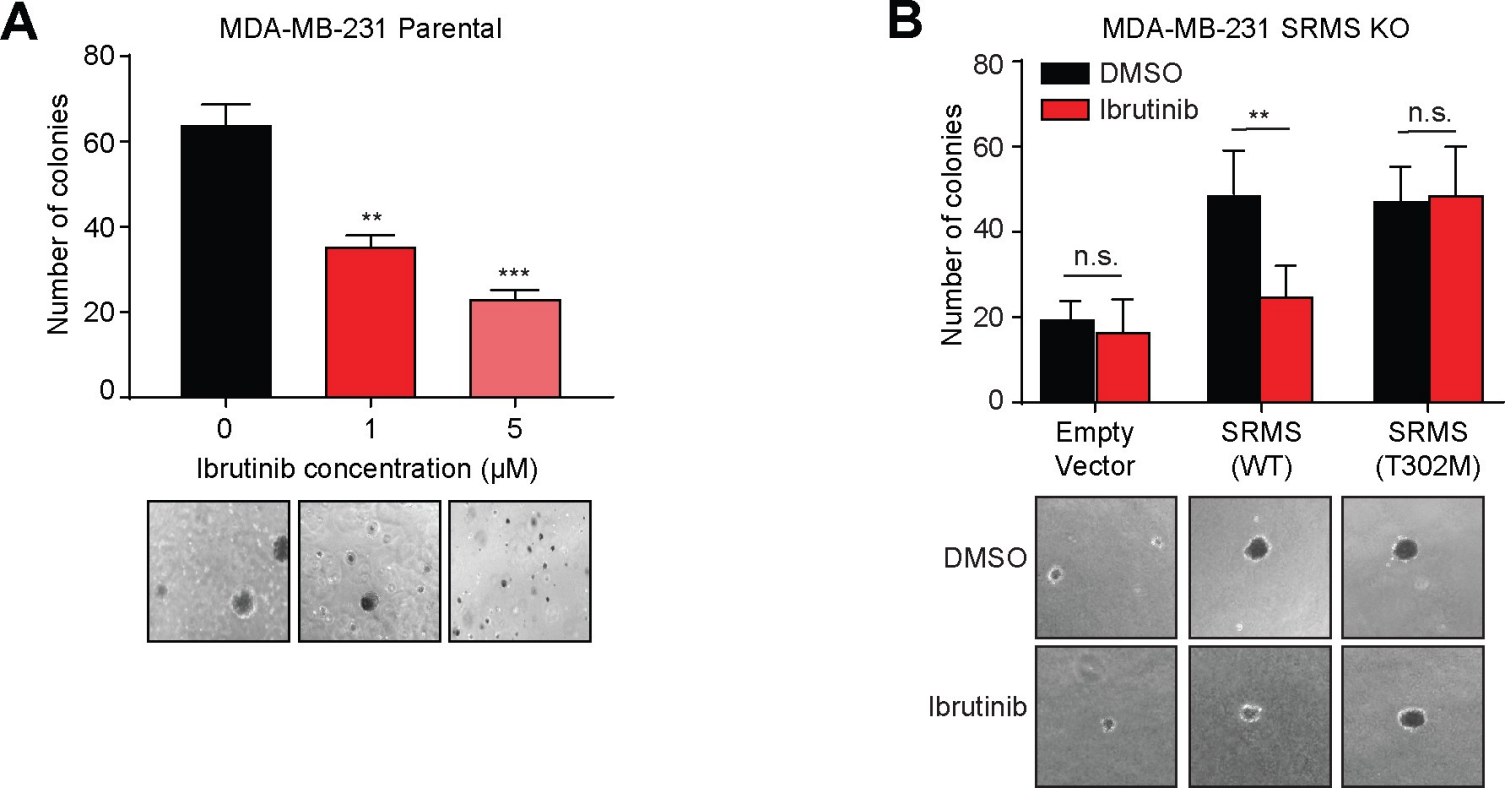
Ibrutinib inhibits cancer cell growth through direct inhibition of SRMS. (**A**) Ibrutinib suppresses anchorage-independent growth. MDA-MB-231 cells were sparsely seeded in soft agar and treated with DMSO or the indicated concentration of ibrutinib for 23 days. Mean + standard deviation of *n* = 3 replicates is shown along with representative images. **p < 0.01, ***p < 0.001. (**B**) SRMS kinase activity promotes anchorage-independent growth. The indicated constructs were expressed in SRMS KO MDA-MB-231 cells. The cells were sparsely seeded in soft agar and treated for 20 days with DMSO or 0.5 μM ibrutinib. Mean + standard deviation of *n* = 3 replicates is shown along with representative images. ns *p* > 0.05, **p < 0.01. The data underlying the figure can be found in S1 Data. KO, knockout; n.s., non-significant (*p*>0.05); SRMS, Src-related kinase lacking C-terminal regulatory tyrosine and N-terminal myristylation sites; WT, wild-type.

Ibrutinib inhibited anchorage-independent growth in parental MDA-MB-231 cells in a dose-dependent manner (Fig 8A). In contrast, SRMS knockout cells were insensitive to ibrutinib (Fig 8B, S8A and S8B Fig). Reintroduction of wild-type SRMS rescued growth in SRMS knockout U2OS and MDA-MB-231 cells and resensitized the cells to ibrutinib (Fig 8B, S8A and S8B Fig). On the other hand, introduction of ibrutinib-resistant mutant SRMS(T302M) or constitutively active AKT rescued growth but did not resensitize to ibrutinib (Fig 8B, S6F, S8A, and S8B Figs). Thus, ibrutinib inhibits growth of human breast cancer and osteosarcoma cell lines via direct inhibition of the SRMS kinase. Taken together, our data indicate that SRMS is a genetic vulnerability that deserves further exploration as a potential therapeutic target in diverse oncological settings.

## Discussion

SRMS is a nonreceptor tyrosine kinase of the BRK family and is structurally similar to BRK and FRK. It is expressed in multiple tissues and at multiple developmental stages within the vertebrate lineage [3,33]. Analyses of SRMS mRNA expression and protein abundance have hinted that SRMS may serve as a biomarker for human diseases such as metastatic breast cancer and Parkinson’s disease [7,31,34]. Although several SRMS substrates have been reported, SRMS kinase activity has not previously been demonstrated to play a physiologically important role in any particular aspect of cellular or organismal biology [3,6,7,10,33–35]. In our prior work aimed at developing high-throughput methodologies for identifying mechanisms of action of bioactive natural products, we serendipitously identified SRMS as a negative regulator of autophagy in human cancer cells [8]. Here, we report the identification of FKBP51 as a bona fide endogenous substrate of SRMS and map the downstream consequences of SRMS-mediated FKBP51 phosphorylation. Importantly, we demonstrate that SRMS directly inhibits the FKBP51-PHLPP phosphatase complex, which otherwise would inactivate the PI3K-AKT signaling pathway. Thus, SRMS promotes sustained PI3K-AKT signaling, which in turn plays very well-established roles in positively regulating cell growth and survival while inhibiting cell death and autophagy [15,23]. We illuminate SRMS overexpression as a previously unappreciated driver of aberrant PI3K-AKT signaling in human cancer and demonstrate that SRMS is a targetable vulnerability worth further investigation in diverse oncological settings. Interestingly, the human SRMS gene is located on chromosome 20q, which is a very common site of copy number gain in pediatric and adult solid tumors from diverse tissues of origin [31,36–43]. The most common 20q gains are not focal but instead encompass nearly the entire chromosome arm, suggesting that multiple genes on 20q may contribute to tumorigenesis. Our work here nominates SRMS as a candidate gene that may help to explain why 20q copy number gains support tumor growth. Future studies should investigate whether 20q gain predicts sensitivity to SRMS inhibition.

Both SRMS and FKBP51 are evolutionarily restricted to the bony vertebrate lineage [24]. Mice lacking either SRMS or FKBP51 remain viable and fertile [3,44]. Thus, the SRMS-FKBP51 signaling pathway is relatively recent from an evolutionary standpoint and is not essential at the cellular or organismal level. Despite its nonessentiality, this pathway does appear to play major roles in diverse human diseases and disorders. For example, FKBP51 expression varies from individual to individual based on genetic polymorphisms and environmental factors, and this variation correlates with sensitivity to trauma and to diverse pharmacological agents ranging from chemotherapies to anti-depressants [11,12,22,45–47]. A protein-coding polymorphism in FKBP51 has been identified in patients with Paget’s disease of bone [21], and the location within the protein’s primary structure (V55L) is directly adjacent to the SRMS phosphorylation site we have identified and reported here (Y54). Interestingly, the reported human V55L polymorphism leads to loss-of-function, whereas we report here that mutation of the adjacent SRMS phosphorylation site (Y54F) leads to gain-of-function, with respect to the FKBP51-PHLPP phosphatase complex. How these mutations affect posttranslational modifications, prolyl isomerase activity, structure, and molecular interactions of FKBP51 warrants further in-depth investigation.

Whereas the role of FKBP51 in preventing Paget’s disease of bone appears to be independent of autophagy regulation, the ability of the FKBP51-PHLPP-AKT-BECN1 complex to induce autophagy is determinant of outcome in other settings including antidepressant treatment efficacy and coronavirus infection [21,22,48]. Members of this protein complex have been investigated as potential targets for novel therapeutic development, as have enzymatic regulators of this core complex including SIRT7, USP49, PINK1, and SKP2 [13,45,46,48–52]. Here, we identify SRMS as a previously unreported negative regulator of FKBP51 that is readily amenable to pharmacological inhibition. We demonstrate that SRMS inhibition increases the activity of the FKBP51-PHLPP complex to inactivate AKT and induce Beclin 1-dependent autophagy. This suggests that SRMS inhibition might be therapeutically useful in settings where FKBP51 activity or Beclin 1-dependent autophagy has been reported to modulate disease progression or therapeutic response. Further investigations are needed to test these predictions and to investigate how the various posttranslational modifications of FKBP51 are coordinated to regulate FKBP51 in human health and disease.

We have shown here that ibrutinib (PCI-32765) can inhibit SRMS in vitro and in cells. Ibrutinib is an inhibitor of BTK, which is in the TEC family of nonreceptor tyrosine kinases. Since 2013, ibrutinib was approved by the FDA for treating B cell malignancies. Genetic ablation or knockdown of BTK significantly reduces tumor growth in mouse model of chronic lymphocytic leukemia (CLL) or lymphoma cells [53,54]. Ibrutinib blocks progression of CLL by targeting of BCR pathway through regulation of downstream kinases such as SYK, BTK, or PI3K [54–56]. Interestingly, BTK is a major kinase expressed in lymph node, spleen, tonsil, lung, and bone marrow, but not expressed in breast. Also, pathological BTK is especially up-regulated in CLL and myeloma but not in other types of cancer [57,58]. However, it has been reported that an isoform of BTK (BTK-C) is expressed in breast cancer cells and enhances cancer cell proliferation. Ibrutinib also induces G1-S cell cycle arrest and apoptosis in HER2^+^ breast cancer cells [59]. Moreover, tumor growth of the Her2+ breast cancer cell line SKBR3 in a xenograft mouse model was inhibited by the injection of ibrutinib [59]. Interestingly, SKBR3 breast cancer cells have high expression of SRMS protein levels [7]. Taken together, ibrutinib has potential as an anticancer drug in the setting of breast cancer and may act through direct inhibition of multiple kinases including BTK-C, Her2, SRMS, and others depending on the specific context. Importantly, we have used genetic manipulation of SRMS alone and in combination with ibrutinib treatment to determine which effects of ibrutinib are due specifically to inhibition of SRMS as opposed to other known or unknown targets of ibrutinib. Therefore, we can unequivocally report that ibrutinib attenuates growth of MDA-MB-231 triple-negative breast cancer cells and U2OS osteosarcoma cells by directly inhibiting SRMS kinase activity. Similarly, although ibrutinib has been previously reported to induce autophagy in diverse contexts [60–62], we carefully demonstrate here that ibrutinib activates autophagy via direct inhibition of the SRMS kinase. It is interesting to note that induction of autophagy is sufficient to extend life span in mice and other animals, and ibrutinib has recently been reported to extend maximal life span in a progeroid mouse model [63]. Whether this particular effect of ibrutinib involves SRMS inhibition remains to be tested. It should be noted that while ibrutinib does inhibit SRMS, it was not optimized for that purpose and is a much more effective inhibitor of its originally intended target, BTK. Therefore, it will be critical to develop more potent and selective inhibitors of SRMS in order to provide tool compounds for investigative purposes and, if warranted, SRMS inhibitors for therapeutic use in the clinic.

In conclusion, the vertebrate tyrosine kinase SRMS restrains autophagy and drives cell growth by phosphorylating FKBP51 to prevent PHLPP-mediated inactivation of AKT. SRMS is amenable to pharmacological inhibition and may represent a previously unrecognized target for manipulating cell growth and autophagy for therapeutic benefit.

## Method details

### Cell culture, transfections, and nutrient modulation

SRMS knockout and wild-type MEFs were isolated from embryonic offspring of *Srms* heterozygous mice [3] (RIKEN, Accession No. CDB0008K, http://www.clst.riken.jp/arg/mutant%20mice%20list.html). MEFs, MDA-MB-231, HeLa, HEK293FT, and U2OS cells were cultured at 37°C in a 5% CO_2_ atmosphere in DMEM high-glucose GlutaMAX supplemented with 10% FBS, 100 units/mL penicillin, 100 units/mL streptomycin. MDA-MB-435 and MCF7 cells were grown in RPMI supplemented with 10% FBS, 100 units/mL penicillin, 100 units/mL streptomycin. siRNA and plasmid transfections were performed using Lipofectamine RNAiMAX, Lipofectamine 3000 (Invitrogen, Carlsbad, California, USA) and Metafectene (Biontex, Cambridge, Massachusetts, USA) according to the manufacturers’ protocol. Starvation was performed by rinsing the cells and replacing the media with EBSS (Invitrogen) for 4 hours. Feeding was performed by rinsing the cells and replacing the media with fresh complete growth media for 4 hours.

### Generation of stable cell lines

MDA-MB-231 SRMS KO and U2OS SRMS KO cells were transfected with either FLAG-vector, FLAG-SRMS, Flag-SRMS K258A, or Flag-SRMS T302M using Lipofectamine 3000 according to the manufacturer’s protocol in 10 cm^2^ plates. After 48 hours, cells were selected with 1 μg/mL of puromycin over 2 weeks. MCF7 cells were transfected with HisMax-Xpress-vector, HisMax-Xpress-Beclin1, or HisMax-Xpress-Beclin1S234/295A using Lipofectamine 3000 according to the manufacturer’s protocol in 10 cm^2^ plates. After 48 hours, cells were selected with 300 μg/mL of Zeocin over 2 weeks.

### Purification of recombinant SRMS

Codon optimized sequence for expression of human SRMS kinase domain (residues 221 to 486) in *Spodoptera frugiperda* was cloned into pDEST20. For the T302M mutant, the following primers were used to perform site-directed mutagenesis: T302M.FOR: CGAACCGGTTTATATTGTGATGGAACTGATGCGCAAAGGCA, T302M.REV: TGCCTTTGCGCATCAGTTCCATCACAATATAAACCGGTTCG. Recombinant bacmid DNA was generated using DH-10 Bac cells, purified, and transfected into SF9 cells using Cellfectin II (Thermo Fisher, Waltham, Massachusetts, USA). Cells were incubated at 27°C for 5 to 7 days, and virus was collected and further amplified to achieve a high multiplicity of infection. For protein production, virus was added to Hi5 cells and the cells harvested after 72 hours. Cells were lysed in buffer containing 50 mM HEPES (pH 7.5 to 8.0), 200 mM NaCl, 5 mM DTT, 10% Glycerol and protease inhibitor cocktail. Purification was by GST affinity chromatography followed by size-exclusion chromatography in 50 mM HEPES (pH 7.5), 100 mM NaCl, 2 mM DTT, 5% Glycerol. The purified SRMS protein was activated by addition of 100 μM ATP and 10 mM magnesium and incubation at 4°C overnight.

### SRMS substrate mobility shift assay

SRMS activity was measured using a peptide substrate mobility shift assay assembled from purified components. The SRMS substrate was 5-FAM-EFPIYDFLPAKKK-CONH2. Final reaction conditions were 25 nM SRMS protein, 100 mM HEPES (pH 7.5), 0.015% Brij-35, 5.0% DMSO, 0.004% Tween 20, 10 mM MgCl2 and 2 mM DTT, 1 uM peptide 27, and 100 uM ATP. If inhibitors or DMSO were used, they were allowed to incubate with SRMS for an hour at room temperature prior to initiation of the assay by adding peptide and ATP. Endpoint assays were run for 3 hours at which time the reaction was quenched with 40 mM EDTA. Data were collected using a LabChip EZ Reader (PerkinElmer, Waltham, Massachusetts, USA) and analyzed using GraphPad (V8.2.1).

### Modeling of SRMS:ibrutinib binding

A homology model of SRMS kinase domain was generated using SWISS-MODEL [64] using the crystal structure of BTK kinase (PDB ID: 2NRU) as a template. The enzyme structures were prepared using the Schrodinger Protein Preparation Wizard software package [65]. Missing amino acid side chains and hydrogen atoms were added. The partial charges for each of the amino acid atoms were assigned using the OPLS_2005 force field. Ibrutinib docking was done using the Glide application in the Schrodinger suite (Version: Release 2019–3) [65], using the default parameters. The binding pose shown had the highest Glide docking score. The pose was validated by superposition with the co-crystal structure of ibrutinib with BTK (5P9J), which showed a highly similar binding mode.

### 3xFLAG-tagged pull-down

HEK293 cells stably expressing 3xFLAG-SRMS K258A or 3xFLAG-vector were cultured in ten 15-cm plates. Cells were washed in cold PBS and scraped in cold PBS, spun down, and resuspended in FLAG Buffer (50 mM Tris (pH 7.4), 150 mM NaCl, 1 mM EDTA, 1% TritonX-100, 1X protease inhibitor cocktail) for 15 minutes on ice. Lysate were spun down and filtered through a 0.45-μm filter and incubated with anti-FLAG beads (Sigma-Aldrich A2220) for 4 hours rotating at 4°C. And then, beads were washed by 1xTBS 5 times and eluted for 4 fractions by elution buffer (200 pg/ul FLAG peptide in 1xTBS) at 4°C rotator, 30 minutess each fraction. Eluted proteins were prepared with SDS sample buffer and run on SDS-PAGE gel to submit entire sample for mass-spec analysis.

### Immunoprecipitation and NTA pull-down analysis

Cells were lysed in 50 mM Tris-HCl (pH 8.0) containing 150 mM NaCl, 0.5% NP-40, 1 mM PMSF, tyrosine protein phosphatase inhibitor (Sigma St. Louis, Missouri, USA), and 1x protease inhibitor cocktail (Sigma). Cell lysates were incubated with indicated antibodies for 2 hours at 4°C and then with 30 μl of 50% slurry of protein A-conjugated Sepharose for the next 1 hour. For pull-down analysis, cell lysates were prepared as above and treated with NTA resins (Qiagen Hilden, Germany). Cell lysates were incubated with indicated antibodies for 2 hours at 4°C and then with 50 μl of 50% slurry of protein A/G-conjugated dynabeads for the next 10 minutes.

### Purification of FKBP51 and GST pull-down assay

GST and GST-FKBP51 constructs were expressed in *Escherichia coli* (BL21 strain) and cultured in YT media. Protein induction was initiated with 1 mM isopropylthio-β-_D_-galactoside to bacterial cultures at *D*_0.6_. Cell extracts were subjected to column chromatography using glutathione resins. Proteins bound to the columns were eluted as recommended by the manufacturers. GST pull-down assay was performed using purified GST and GST-FKBP51 protein incubating with purified Myc-SRMS in vitro transcribed and translated protein (TnT Quick Coupled Transcription/Translation Systems from Promega). In vitro binding assay was performed using purified GST-FKBP51 bound to glutathione Sepharose beads for 1 hour in binding buffer (25 mM Tris (pH 8.0), 2.7 mM KCl, 137 mM NaCl, 0.05% v/v Tween-20, and 10 mM 2-mercaptoethanol) and then blocked for 1 hour in binding buffer containing 5% (w/v) milk powder. In vitro translated Myc-SRMS or Myc-SRMS K258A proteins were then incubated with the bound beads for 1 hour, washed, and the proteins were eluted in SDS-sample buffer and subjected to SDS-PAGE and immunoblotting.

### Image-based analysis of autophagosomes and autolysosomes

Autophagosomes were visualized using either U2OS cells stably expressing the GFP-LC3 reporter or immunocytochemistry to detect endogenous LC3. In both cases, cells were seeded on coverslips and treated as indicated. Cells were then fixed by incubation with 3.7% paraformaldehyde in PBS for 10 to 15 minutes, washed 3 times with 0.1% (v/v) Triton X-100 or PBS, permeabilized with 0.5% Triton X-100 for 5 minutes or 100 μg/ml digitonin for 10 minutes, and treated with 3% BSA in PBS. For endogenous LC3 detection, primary antibody incubation was carried out at 1:200 dilution for 1 hour, and secondary antibody incubation was carried out 1:500 dilution for 30 minutes (with 3 washes after each incubation). Cells were then mounted, observed, and imaged using a confocal laser scanning microscope (Leica SP8 Confocal). Images were either blinded and the number of LC3-positive puncta per cell counted manually, or LC3-positive puncta were identified and counted in an automated fashion using Photoshop (Adobe). Autolysosomes were visualized using either acridine orange or the tandem monomeric RFP-GFP-LC3 reporter. For acridine orange, cells were seeded on coverslips and stained with 1 μg/mL acridine orange for 20 minutes. Cells were then observed and imaged using a confocal laser scanning microscope (Leica SP8 Confocal) at 609 to 695 nm to detect acidic vesicles and at 511 to 566 nm to detect the nonacidic acridine orange signal. Image analysis was performed using Cell Profiler. For the tandem monomeric RFP-GFP-LC3 reporter, U2OS cells stably expressing RFP-GFP-LC3 were plated at 2 × 10^5^ cells per well in 12-well plates (Corning #3513) and cultured at 37°C in a 5% CO_2_ atmosphere in DMEM high-glucose GlutaMAX supplemented with 10% FBS, 100 units/mL penicillin, and 100 units/mL streptomycin for 24 hours. Cells were treated as indicated and transferred to an IncuCyte S3 (Sartorius, Gottingen, Germany). The cells were imaged using the 20x objective at 440 to 480 nm to detect GFP and at 565 to 605 nm to detect RFP signal. Image analysis was performed using IncuCyte S3 software, and puncta were counted by Image J.

### Autophagic flux assay

Autophagic flux was measured using the Autophagy LC3 HiBiT Reporter Assay System from Promega. U2OS HiBiT-HaloTag-LC3 cells (Promega #GA1050) were cultured at 37°C in a 5% CO_2_ atmosphere in DMEM high glucose GlutaMAX supplemented with 10% FBS, 100 units/mL penicillin, 100 units/mL streptomycin, and 250 g/mL G418 (ThermoFisher #10131). Cells were plated in G418-free media at 8 × 10^3^ cells per 80 μl into each well of clear-bottom 96-well white plates (Costar #3903). Twenty-four hours later, cells were transfected with the indicated siRNA oligonucleotides using RNAiMax (Invitrogen #13778) or treated with DMSO, ibrutinib, or acalabrutinib as indicated. Seventy-two hours after transfection or 24 hours after compound treatment, the plate was equilibrated to room temperature for 15 minutes. A volume of Nano-Glo HiBiT Lytic Reagent (Promega #N3030) equal to the volume in each well was added and mixed by orbital shaker (300 rpm) for 5 minutes. Luminescence was measured using a PerkinElmer plate reader and normalized to either siControl-transfected wells or DMSO-treated wells as appropriate.

### In vitro kinase assay

Myc-SRMS was produced using TNT coupled reticulocyte lysate system (Promega). In vitro kinase assay was performed using GST-FKBP51 and Myc-SRMS in a reaction volume 50 μl compromising 20 μl kinase buffer (Cell Signaling, Danvers, Massachusetts, USA) with or without 200 μM ATP. The reaction mixture was incubated at 30°C for 30 minutes. To terminate the reaction, 2X SDS sample buffer was added, and the samples were boiled at 100°C. The samples were then resolved via SDS/PAGE.

### Crisper/Cas9 knockouts

For the generation of U2OS or MDA-MB-231 SRMS knockout cells, gRNA of SRMS-CACCGTATGACTTCACGGCGCGGTG was designed using the CRISPR design tool at http://crispor.tefor.net and was cloned in pSpCas9(BB)-2A-Pro(PX495) V2.0 (Addgene, Cat#62988). We transfected 2 μg of gRNA using Effectene (Qiagen) for 24 hours, changed to fresh media, and incubated for an additional 24 hours. Cells were then treated with 1 μg/ml of puromycin for 3 days. Single cells were sorted by flow cytometry into 96-well plates, clonally expanded, and verified for the desired modification via targeted next-generation sequencing and analysis with *CRIS*.*py* [66]. Briefly, the target region was amplified with locus-specific primers SRMS.DS.F– 5′ CCTGCCAGTTCCTCTTCCG 3′ and SRMS.DS.R– 5′CTGCGTGCGAAGATGTAGC 3′ containing universal 5′ tails on the forward (5′-CACTCTTTCCCTACACGACGCTCTTCCGATCT-3′) and reverse (5′-GTGACTGGAGTTCAGACGTGTGCTCTTCCGATCT-3′) primers. PCR amplifications were performed with Mytaq DNA Polymerase (Bioline, London, United Kingdom) according to the manufacturer’s protocol. A secondary indexing PCR was performed in order to maintain sample identity after pooling. Next-generation sequencing was performed using the Illumina Miseq platform, and 2 × 250 bp reads were obtained and analyzed to identify knockout clones.

### Subcellular fractionation

Subcellular fractionation was performed as described with minor modifications [67]. Briefly, cells were suspended in 10 mM HEPES (pH 7.8), 10 mM KCl, 1.5 mM MgCl_2_, 0.34 M sucrose, 10% (v/v) glycerol, 1 mM DTT, 0.1% (v/v) Triton X-100, and 1x protease inhibitor cocktail. After incubation for 5 minutes on ice, the samples were subjected to centrifugation for 5 minutes at 3,000*g*. The supernatants were again centrifuged at 15,000*g* for 15 minutes, and the soluble fractions were used as the cytosolic fractions. The pellets from the low-speed centrifugation were resuspended in 3 mM EDTA, 0.2 mM EGTA, 1 mM DTT, and 1x protease inhibitor cocktail, incubated on ice for 30 minutes, and centrifuged for 5 minutes at 3,000*g*. The insoluble fractions were used as the nuclear fractions.

### Xenograft assays

MDA-MB-231 cells and MDA-MB-231 SRMS KO cells were subcutaneously injected into the upper thighs of NOD.*Cg-Prkdc*^*scid*^*Il2rg*^*tm1Wjl*^/SzJ (NOD *scid* gamma) mice. MDA-MB-231 SRMS KO cells (clone G10) that stably express Flag empty vector, Flag-SRMS wild-type, or Flag-SRMS K258A (kinase-dead) mutant were subcutaneously injected into the upper thigh of NOD.*Cg-Prkdc*^*scid*^*Il2rg*^*tm1Wjl*^/SzJ (NOD *scid* gamma) mice. Tumor volumes were calculated as (*a*x*a*x*b*)/2, in which *a* is the smallest diameter and *b* the largest. Animal studies were approved by the St. Jude Institutional Animal Care and Use Committee (IACUC) under approval number 599-100565-10/18.

### Clonogenic growth and anchorage-independent growth assays

For clonogenic growth assays on plates, wild-type, knockout, or rescued cells were plated in 6-well plates in triplicate. After 2 to 3 weeks, cells were fixed and stained with 0.05% (w/v) crystal violet and counted. For anchorage-independent growth soft agar assays, cells were suspended in 0.375% (w/v) Noble agar (Difco, San Jose, California, USA) supplemented with standard growth medium and overlaid on 0.5% (w/v) Noble agar. Cells were incubated for 3 to 4 weeks before colonies ≥100 μm in size were counted.

### RNA interference

siRNA transfections were performed using Lipofectamine RNAiMAX or Dharmafect 1 according to the manufacturer’s protocol. The siRNA oligonucleotides were purchased from Dharmacon (Fig 1) or Sigma (all other figures). The Dharmacon siRNA oligonucleotide sequences were: siSRMS #1, 5′-UCACUGACCUCGCCAAGGA; siSRMS#2, 5′-GCAGAAGGGACGGCUCUUU; siSRMS #3, 5′-GAUCAAGGUCAUCAAGUCA. The Sigma siRNA oligonucleotide sequences were: siRNA Universal Negative Control (Sigma, SIC001), siSRMS #1, 5′-GCUCCAAGAUCCCGGUCAA; siSRMS #2, 5′-GCUCUUCCUUGCGCUCUAU; siSRMS #3, 5′-CUGUGUACAUCGUCACGGA; siSRMS #4, 5′-GGCUACAUCUUCGCACGCA; siFKBP51 #1, 5′-GCCUAAAUUUGGCAUUGAA; siFKBP51 #2, 5′-GAGACAAAGUUUAUGUCCA; siFKBP51 #3, 5′-GGAUAUACGCCAACAUGUU; siFKBP51 #4 5′-CAGUGUUUAAUGUAAAGUU.

### Antibodies

Commercial antibodies used: anti-SRMS (Santa Cruz, sc-374524), anti-Xpress (Invitrogen, 46–0528), anti-GAPDH (Cell Signaling Technology, 2118S), anti-HA (Santa Cruz, sc-805), anti-FLAG (Sigma, F3165), anti-Myc (Santa Cruz, sc-40), anti-β-Actin (Sigma, A1978), anti-Tubulin (Cell Signaling Technology, 3873S), anti-FKBP51 (Cell Signaling Technology, 8245S), anti-GSK3β (Cell Signaling Technology, 12456S), anti-phospho-GSK3β (Cell Signaling Technology, 5558S), anti-Akt (Cell Signaling Technology, 4691S), anti-phospho-Akt S473 (Cell Signaling Technology, 4060S), anti-phospho-Akt T308 (Cell Signaling Technology, 13038S), anti-SQSTM1/p62 (Cell Signaling Technology, 5114S), anti-LC3 (MBL, PM036), anti-LC3 (Cell Signaling Technology, 3868S), anti-p-Tyrosine (Cell Signaling Technology, 9411S), anti-p-Y-1000 Multimab (Cell Signaling Technology, 8954S), anti-Beclin-1 (Cell Signaling Technology, 3495S), anti-Fox01 (Cell Signaling Technology, 97635S), anti-p-Fox01 S256 (Cell Signaling Technology, 84192S), anti-mCherry (Abcam, ab167453), anti-TFIIH p89 (Santa Cruz, s-19).

## Supporting information

S1 Fig SRMS restrains autophagy through its kinase activity. (**A**) The efficiency of depletion of endogenous SRMS protein by RNAi vs. CRISPR/Cas9-mediated gene editing was compared in U2OS cells by western blot. (**B**) Autophagosome biogenesis and autophagic flux were compared between SRMS KO and WT MEFs using LC3 immunocytochemistry. Representative images are shown. (**C**) Number of LC3-positive puncta (i.e., autophagosomes) per cell was counted from *n =* 5 images (205–276 MEF cells) per condition. **p < 0.01, *t* test. (**D**) Autophagic flux was compared between SRMS WT and KO MEFs. The average number of LC3-positive puncta degraded per hour per cell was calculated from the data presented in S1B and S1C Fig; *n* = 5 images (>200 cells) per condition. **p < 0.01, *t* test. (**E**) Parental and SRMS KO MDA-MB-231 cells were treated with DMSO or the indicated concentration of Bafilomycin A1 for 2 hours. Lysates were collected and analyzed by western blot. LC3 accumulation is heightened in SRMS KO cells relative to parental controls. (**F**) SRMS(K258A) is enzymatically inactive. Myc-SRMS(WT) and Myc-SRMS(K258A) were expressed in SRMS KO U2OS cells. Cell lysates were immunoprecipitated with anti-Myc antibody and blotted with anti-p-Tyr antibody to detect SRMS autophosphorylation. (**G**) Myc-SRMS(WT) and Myc-SRMS(K258A) were transiently expressed in U2OS cells stably expressing GFP-LC3. Cells were fixed and stained with anti-Myc antibody to detect transfected cells. Representative images are shown. For quantitation, see Fig 1E. (**H**) MS/MS fragmentation data for human SRMS AA 374–387 sequence LLKDDIY(+79.97)SPSSSK, M/z 810.3703, z2, showing b/y ions. MS/MS fragment ions at M/z 941.42 (b7) and M/z 922.34 (y8) represent characteristic ions that unambiguously identify Y380 phosphorylation. The data underlying the figure can be found in S1 Data. IP, immunoprecipitation; KO, knockout; MEF, mouse embryonic fibroblast; MS/MS, tandem mass spectrometry; RNAi, RNA interference; SRMS, Src-related kinase lacking C-terminal regulatory tyrosine and N-terminal myristylation sites; WT, wild-type.
(PDF)

S2 Fig Ibrutinib blocks SRMS kinase activity and increases autophagy. (**A**) SRMS overexpression increases p-Tyr immunoreactivity. HeLa cells were transiently transfected with the indicated constructs. Twenty-four hours later, lysates were collected and analyzed by western blot using the indicated antibodies. (**B**) Ibrutinib inhibits SRMS activity in a dose-dependent manner. HEK293 cells stably expressing Myc-SRMS(WT) were treated with the indicated compounds at the indicated concentrations for 2 hours. Cell lysates were subjected to immunoblotting with indicated antibodies. (**C**) Ibrutinib activates LC3 lipidation in a dose-dependent manner. Parental MDA-MB-231 cells were treated with ibrutinib at the indicated concentrations for 4 hours. Cell lysates were immunoblotted with anti-LC3 antibody. (**D**) Ibrutinib activates autophagosome formation in a dose-dependent manner. U2OS cells stably expressing GFP-LC3 were treated with ibrutinib at the indicated concentrations for 4 hours. GFP-LC3 puncta were detected by confocal microscopy. Representative images are shown. (**E–G**) Ibrutinib activates autophagy in an SRMS-dependent manner as measured by acridine orange. Parental or SRMS KO U2OS cells were treated with DMSO or ibrutinib (0.5 μM or as indicated) for 8 hours. Cells were then stained with 1 μg/mL acridine orange for 20 minutes and imaged at the indicated wavelengths. Representative images are shown (**E**) along with quantitation (**F, G**). For panel F, *n =* 10 images (123 cells) for parental and *n* = 8 images (130 cells) for SRMS KO. G shows mean +/− standard deviation of *n* = 10 images (123 cells), *n* = 11 images (131 cells), *n* = 8 images (100 cells), and *n* = 9 images (79 cells) for parental and *n* = 8 images (130 cells), *n* = 8 images (139 cells), *n* = 8 images (128 cells), and *n* = 9 images (181 cells) for SRMS KO (left to right). *p < 0.05, **p < 0.01, ***p < 0.001, ****p < 0.0001, *t* test. (**H**) Ibrutinib induces autophagosome biogenesis and autophagosome–lysosome fusion. U2OS cells stably expressing RFP-GFP-LC3 were treated with 1 μM ibrutinib for 12 hours. Cells were imaged by IncuCyte. Cells having more than 3 puncta were counted. Mean + SD of *n* = 15 cells per condition is shown. **p < 0.01, ***p < 0.001, ****p < 0.0001, *t* test. (**I**) Ibrutinib induces autophagic flux, but acalabrutinib does not. U2OS Autophagy LC3 HiBiT Reporter cells were plated at 8,000 cells per well. (**J**) SRMS kinase activity restrains LC3 lipidation. Myc-SRMS(WT) and Myc-SRMS(T302M) were expressed in SRMS KO U2OS cells and treated with 0.5 μM ibrutinib for 4 hours. Cell lysates were immunoprecipitated with anti-Myc antibody and blotted with indicated antibodies. The data underlying the figure can be found in S1 Data. IP, immunoprecipitation; KO, knockout; SRMS, Src-related kinase lacking C-terminal regulatory tyrosine and N-terminal myristylation sites; WT, wild-type.
(TIF)

S3 Fig SRMS interacts with FKBP51 through its kinase domain. (**A, B**) SRMS interacts with FKBP51 through its kinase domain. SRMS truncated constructs (schematized in A) were transfected with Flag-FKBP51 in HEK293FT cells. Cell lysates were subjected to IP with anti-Myc and blotted with indicated antibodies (**B**). (**C**) FKBP51 interacts with SRMS through its TPR1 domain. Schematic representation of the 9 truncated FKBP51 constructs. (**D, E**) Each FKBP51 truncated construct was transfected with Myc-SRMS in HEK293FT cells. Cell lysates were subjected to anti-Flag IP and blotted with indicated antibodies. (**F**) SRMS directly phosphorylates FKBP51. An in vitro kinase assay was performed using GST-FKBP51 and in vitro transcribed/translated Myc-SRMS(WT) or Myc-SRMS(K258A) proteins, in presence or absence of λ-phosphatase as indicated. Tyrosine phosphorylation of FKBP51 was probed via anti-pTyr antibody, and GST-FKBP51 proteins were detected via anti-GST antibody. (**G**) Tyrosine 54 of FKBP5/FKBP51 is evolutionarily conserved, while the paralog FKBP4/FKBP52 has phenylalanine or threonine at the corresponding residue. FKBP51, FK506-binding protein 51; GST, glutathione S-transferase; IB, immunoblot; IP, immunoprecipitation; SRMS, Src-related kinase lacking C-terminal regulatory tyrosine and N-terminal myristylation sites; WT, wild-type.
(TIF)

S4 Fig SRMS accelerates ubiquitination of FKBP51 under nutrient-replete condition. (**A**) SRMS promotes accumulation of cytosolic p-AKT(S473) in a kinase-dependent manner. SRMS KO U2OS cells were reconstituted with empty vector, Flag-SRMS(WT), or Flag-SRMS(K258A) as indicated. Lysates were collected, subjected to subcellular fractionation, and blotted with the indicated antibodies. Lamin B was used as a marker for nuclear fraction and α-tubulin for cytosolic fraction. (**B**) SRMS blocks the interaction between FKBP51 and PHLPP. FKBP51 was transfected with PHLPP and SRMS in 293T cells. Cell lysates were subjected to IP by anti-Flag antibody and blotted with indicated proteins. *nonspecific band. (**C**) WT SRMS or SRMS(K258A) mutant was expressed in SRMS KO U2OS cells. Lysates were collected and subjected to IP with anti-FKBP51 antibody or IgG control as indicated. Input (whole cell lysates) and immunoprecipitates were analyzed by western blot with the indicated antibodies. Note that FKBP51 interacts more strongly with AKT and PHLPP in cells expressing SRMS(K258A) compared to cells expressing WT SRMS. Also note that dephosphorylation of p-AKT(S473) is more prevalent in the whole cell lysate of cells expressing SRMS(K258A) mutant than WT SRMS. (**D**) U2OS cells in which all endogenous FKBP51 alleles were converted to the nonphosphorylatable FKBP51 Y54F mutant by CRISPR/Cas9 exhibit decreased accumulation of p-AKT at Ser 473 and increased LC3 lipidation relative to parental U2OS cells. Cell lysates were immunoblotted with the indicated antibodies. (**E**) The proposed signaling pathway through which SRMS promotes AKT signaling and restrains autophagy. (**F**) Nutrient-dependent FKBP51 reduction is not unique to MDA-MB-231 cells. MDA-MB-435 cells were incubated under starved or fed conditions for 4 hours. Cell lysates were immunoblotted with indicated antibodies. (**G**) FKBP51 is poly-ubiquitinated under fed condition. MDA-MB-231 cells were incubated under starved or fed conditions and treated with 10 nM MG132 for 4 hours. Cell lysates were subjected to IP by anti-FKBP51 antibody and blotted with indicated antibodies. (**H**) Poly-ubiquitination of FKBP51 requires SRMS. Parental or SRMS KO MDA-MB 231 cells (clone 15) were treated with 10 nM MG132 for 4 hours. Cell lysates were subjected to IP with anti-FKBP51 antibody and blotted with indicated antibodies. (**I**) FKBP51 protein stability increases with treatment of MG132 or ibrutinib. MDA-MB-231 cells were treated with 100 μg/mL cycloheximide in the presence of DMSO, 10 nM MG132, or 0.5 μM of ibrutinib for the indicated times. Lysates were immunoblotted with the indicated antibodies. FKBP51, FK506-binding protein 51; GAPDH, glyceraldehyde-3-phosphate dehydrogenase; IgG, immunoglobulin G; IP, immunoprecipitation; KO, knockout; SRMS, Src-related kinase lacking C-terminal regulatory tyrosine and N-terminal myristylation sites; WT, wild-type.
(TIF)

S5 Fig SRMS inhibits the tumor-suppressive activity of Beclin 1 by enhancing AKT-dependent phosphorylation on S295. (**A**) KO of FKBP51 increases abundance of p-AKT at Ser 473. Lysates were analyzed by western blot with the indicated antibodies. (**B, C**) SRMS restrains Beclin 1-dependent autophagy. Parental U2OS cells were transfected with the indicated siRNA oligonucleotides alone or in combination, as indicated. Seventy-two hours later, the cells were fixed and stained with anti-LC3 antibody for imaging (**B**) or lysed and processed for western blot with the indicated antibodies (**C**). From left to right, mean +/− standard deviation of *n =* 3 images (60 cells), *n* = 3 images (48 cells), and *n* = 4 images (113 cells) are shown. *p < 0.1, ***p < 0.001, *t* test. (**D**) Parental and SRMS KO MDA-MB-231 cells were lysed and subjected to IP with anti-Beclin 1 antibody or control anti-IgG as indicated. Input (whole cell lysates) and immunoprecipitates were subjected to western blot analysis with the indicated antibodies. Note that Beclin 1 interacts more strongly with VPS34, UVRAG, and ATG14 (but not Rubicon) in the SRMS KO cells relative to parental control cells. (**E**) Parental MDA-MB-231 cells were treated with DMSO or ibrutinib (0.5 μM concentration for 4 hours). Anti-Beclin 1 IP was performed, and both input (whole cell lysates) and immunoprecipitates were analyzed by western blot with the indicated antibodies. Note that Beclin 1 interacts more strongly with VPS34, UVRAG, and ATG14 (but not Rubicon) in cells treated with ibrutinib relative to DMSO control. The data underlying the figure can be found in S1 Data. FKBP51, FK506-binding protein 51; GAPDH, glyceraldehyde-3-phosphate dehydrogenase; IgG, immunoglobulin G; IP, immunoprecipitation; KO, knockout; siRNA, small interfering RNA; SRMS, Src-related kinase lacking C-terminal regulatory tyrosine and N-terminal myristylation sites.
(TIF)

S6 Fig SRMS kinase activity plays a key role in tumor growth. (**A**) SRMS expression is elevated in human breast and colorectal tumors (red) relative to corresponding normal tissues (gray). *p < 0.05. (**B**) SRMS supports clonogenic growth of U2OS osteosarcoma cells. Parental and SRMS KO U2OS cells were plated sparsely and allowed to grow for 12 days. Colonies were stained with crystal violet, imaged, and counted. Mean + standard deviation of *n =* 3 replicates is shown along with representative images. **p < 0.01, *t* test. (**C**) SRMS supports clonogenic growth of MDA-MB-231 triple-negative breast cancer cells. Parental or SRMS KO MDA-MB-231 cells were seeded sparsely in 6-well plates and allowed to grow for 17 days. Colonies were stained with crystal violet, imaged, and counted. Mean + standard deviation of *n =* 3 replicates is shown along with representative images. **p < 0.01, *t* test. (**D**) The ability of WT SRMS vs. kinase-dead SRMS to rescue the clonogenic growth defect of SRMS KO U2OS cells were compared. Mean + standard deviation of *n* = 3 replicates is shown along with representative images. ns *p* > 0.05, ***p < 0.001, *t* test. (Relates to Fig 6C). (**E**) CRISPR-Cas9-mediated mutation of all FKBP51 alleles to nonphosphorylatable FKBP51 Y54F mutant into U2OS cells impairs growth. Clonogenic growth was compared between parental and FKBP51 Y54F edited cells. Mean + standard deviation of *n =* 3 replicates is shown along with representative images. *p < 0.05. (**F**) Constitutively active AKT rescues the growth defect of SRMS KO cells without conferring sensitivity to ibrutinib. EV or Myr-AKT was transfected in SRMS KO MDA-MB-231 cells. Cells were sparsely seeded in soft agar and treated with DMSO or 0.5 μM ibrutinib for 20 days. Mean + standard deviation of *n* = 3 replicates is shown along with representative images. ns *p* > 0.05, **p < 0.01, *t* test. (**G–I**) SRMS antagonizes the tumor suppressive function of WT Beclin 1 but not of Beclin 1 (S243A, S295A), which is not phosphorylated by AKT [15]. EV, Beclin 1 (WT), or Beclin 1 (S234A, S295A) were expressed in ER+ MCF7 breast cancer cells. SRMS was depleted by RNAi (**G, H**) or inhibited by 0.5 μM ibrutinib (**I**). Lysates were analyzed by western blot using the indicated antibodies (**G**), or cells were plated sparsely in soft agar and allowed to grow for 17 days before being imaged and counted (**H, I**). Mean + standard deviation of *n* = 3 replicates is shown along with representative images (**H, I**). ns *p* > 0.05, *p < 0.05, **p < 0.01, ***p < 0.001, *t* test. The data underlying the figure can be found in S1 Data. EV, empty vector; FKBP51, FK506-binding protein 51; KO, knockout; n.s., non-significant (*p*>0.05); RNAi, RNA interference; SRMS, Src-related kinase lacking C-terminal regulatory tyrosine and N-terminal myristylation sites; WT, wild-type.
(TIF)

S7 Fig Autophagy inhibition by SRMS prevents widespread cancer cell senescence. (**A**) MCF7 breast cancer cells were transfected with the indicated siRNA oligonucleotides alone or in combination in a standard 96-well proliferation assay. siLONRF1 served as a negative control. Seventy-two hours later, growth was measured by Cell Titer Glo assay and normalized to siLONRF1 control (top), and efficiency of knockdown was measured by western blot (bottom). (**B**) The same experiment as described in (**A**) was performed using T47D breast cancer cells. Means + standard deviation of *n* = 3 replicates are shown; ns *p* > 0.05, *p < 0.05, **p < 0.01, *t* test. The data underlying the figure can be found in S1 Data. GAPDH, glyceraldehyde-3-phosphate dehydrogenase; ns, non-significant (*p*>0.05); siRNA, small interfering RNA; SRMS, Src-related kinase lacking C-terminal regulatory tyrosine and N-terminal myristylation sites.
(JPG)

S8 Fig Ibrutinib suppresses tumor cell proliferation through direct inhibition of SRMS. (**A**) The ability of WT SRMS vs. SRMS(T302M) to rescue the clonogenic growth defect and ibrutinib resistance of SRMS KO U2OS cells was compared. Mean + standard deviation of *n* = 3 replicates is shown along with representative images. ***p < 0.001, *t* test. (**B**) The ability of WT SRMS vs. SRMS(T302M) to rescue the clonogenic growth defect and ibrutinib resistance of SRMS KO MDA-MB-231 cells was compared. Mean + standard deviation of *n* = 3 replicates is shown along with representative images. n.s, ***p < 0.001, *t* test. The data underlying the figure can be found in S1 Data. KO, knockout; n.s, non-significant (*p*>0.05); SRMS, Src-related kinase lacking C-terminal regulatory tyrosine and N-terminal myristylation sites; WT, wild-type.
(TIF)

S1 Table Candidate SRMS substrates identified by substrate-trap IP and mass spectrometry. Spectral counts from each experiment are given.
(XLSX)

S1 Data Numerical data underlying all figures. (XLSX)

S1 Raw Images Raw blots and gels underlying all figures. (PDF)

## Abbreviations

BRK: breast tumor kinase
BTK: bruton tyrosine kinase
CLL: chronic lymphocytic leukemia
EBSS: Earle’s balanced salt solution
FKBP51: FK506-binding protein 51
FRK: Fyn-related kinase
GST: glutathione S-transferase
IP: immunoprecipitation
MEF: mouse embryonic fibroblast
RNAi: RNA interference
SH2: Src-homology 2
SH3: Src-homology 3
siRNA: small interfering RNA
SRMS: Src-related kinase lacking C-terminal regulatory tyrosine and N-terminal myristylation sites
TKI: tyrosine kinase inhibitor
