# Supplementary figures and images for: The nonreceptor tyrosine kinase SRMS inhibits autophagy and promotes tumor growth by phosphorylating the scaffolding protein FKBP51
Source: PLoS Biol. 2021 Jun 2;19(6):e3001281. doi: 10.1371/journal.pbio.3001281 (PMC8202955; doi:10.1371/journal.pbio.3001281)

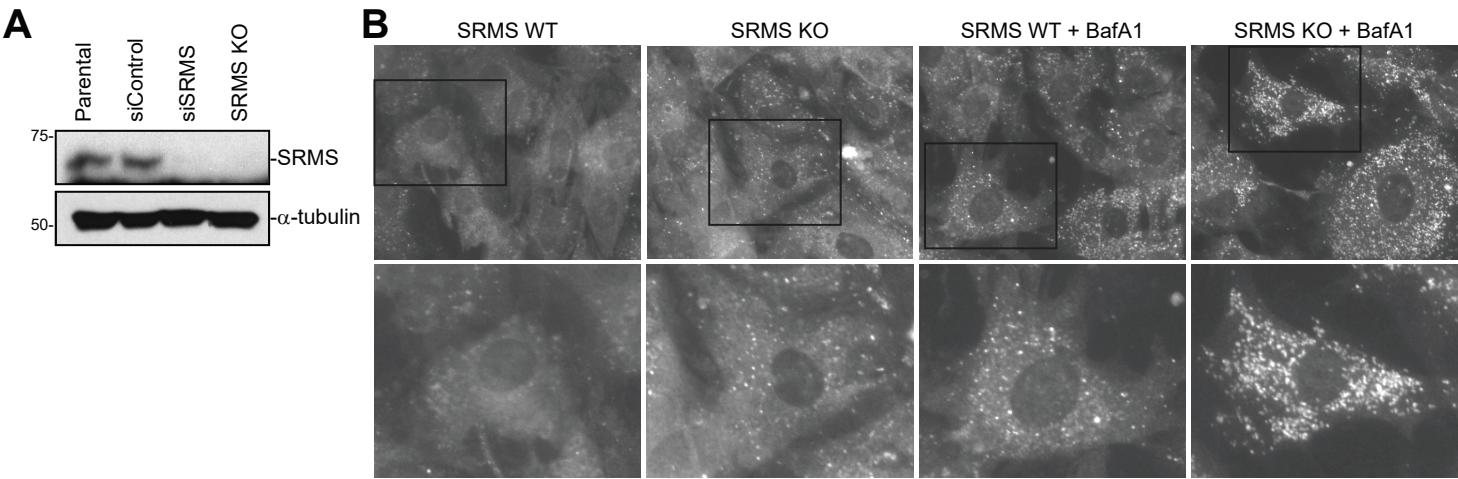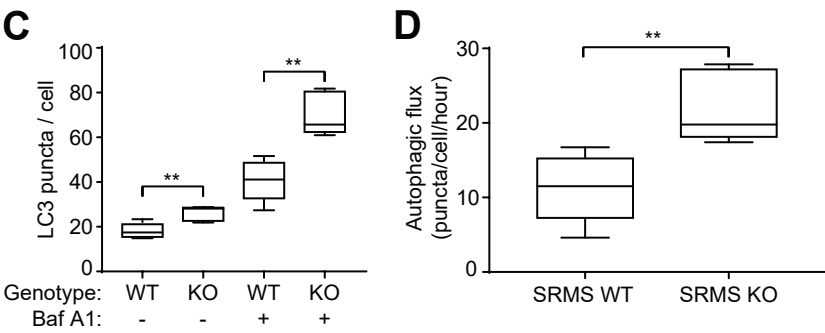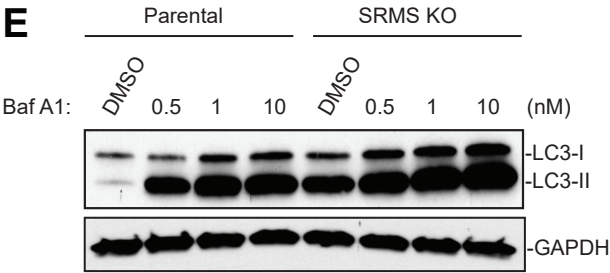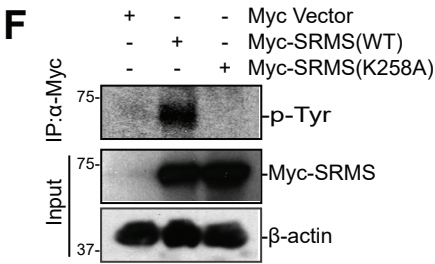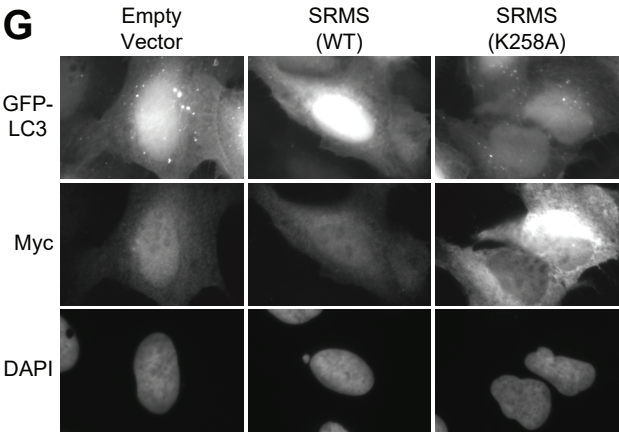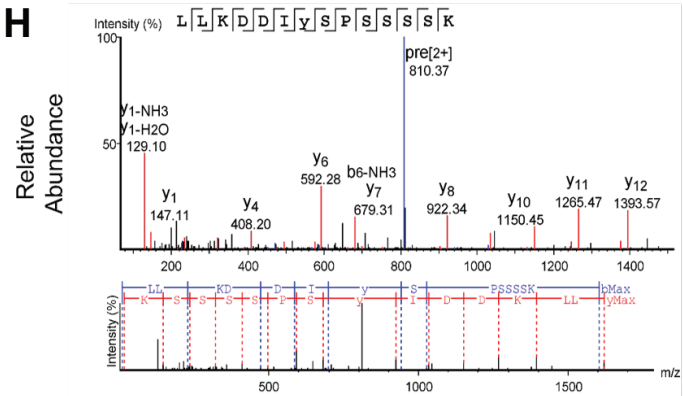

Ion Table & Error Map

| #  | b       | b-H2O   | b-NH3   | b (2+) | Seq       | y       | y-H2O   | y-NH3   | y (2+) | #  |
|----|---------|---------|---------|--------|-----------|---------|---------|---------|--------|----|
| 1  | 114.09  | 96.08   | 97.06   | 57.55  | L         | 1506.67 | 1488.66 | 1489.65 | 753.83 | 14 |
| 2  | 227.17  | 209.17  | 210.15  | 114.09 | L         | 1393.57 | 1375.68 | 1376.56 | 697.79 | 13 |
| 3  | 355.27  | 337.26  | 338.24  | 178.14 | K         | 1265.47 | 1247.46 | 1248.47 | 633.25 | 11 |
| 4  | 470.29  | 452.28  | 453.27  | 235.65 | D         | 1150.45 | 1132.44 | 1133.44 | 575.73 | 10 |
| 5  | 585.32  | 567.31  | 568.30  | 293.16 | D         | 1035.42 | 1017.43 | 1018.41 | 518.22 | 9  |
| 6  | 698.40  | 680.40  | 681.28  | 349.70 | I         | 922.34  | 904.33  | 905.33  | 461.68 | 8  |
| 7  | 941.42  | 923.43  | 924.41  | 471.22 | Y(+79.97) | 810.37  | 792.36  | 793.36  | 340.16 | 7  |
| 8  | 1028.45 | 1010.46 | 1011.44 | 514.74 | S         | 679.31  | 661.31  | 662.30  | 340.16 | 7  |
| 9  | 1125.52 | 1107.51 | 1108.50 | 563.26 | P         | 592.28  | 574.27  | 575.27  | 296.65 | 6  |
| 10 | 1212.56 | 1194.54 | 1195.53 | 607.24 | S         | 495.23  | 477.22  | 478.21  | 248.12 | 5  |
| 11 | 1299.59 | 1281.58 | 1282.56 | 650.29 | S         | 408.20  | 390.19  | 391.18  | 204.60 | 4  |
| 12 | 1386.62 | 1368.61 | 1369.59 | 693.81 | S         | 321.17  | 303.17  | 304.15  | 161.09 | 3  |
| 13 | 1473.65 | 1455.64 | 1456.62 | 737.33 | S         | 234.14  | 216.04  | 217.12  | 117.57 | 2  |
| 14 |         |         |         |        | K         | 147.11  | 129.10  | 130.08  | 74.06  | 1  |

Supplement: S1 Fig — (A) The efficiency of depletion of endogenous SRMS protein by RNAi vs. CRISPR/Cas9-mediated gene editing was compared in U2OS cells by western blot. (B) Autophagosome biogenesis and autophagic flux were compared between SRMS KO and WT MEFs using LC3 immunocytochemistry. Representative images are shown. (C) Number of LC3-positive puncta (i.e., autophagosomes) per cell was counted from n = 5 images (205–276 MEF cells) per condition. **p < 0.01, t test. (D) Autophagic flux was compared between SRMS WT and KO MEFs. The average number of LC3-positive puncta degraded per hour per cell was calculated from the data presented in S1B and S1C Fig; n = 5 images (>200 cells) per condition. **p < 0.01, t test. (E) Parental and SRMS KO MDA-MB-231 cells were treated with DMSO or the indicated concentration of Bafilomycin A1 for 2 hours. Lysates were collected and analyzed by western blot. LC3 accumulation is heightened in SRMS KO cells relative to parental controls. (F) SRMS(K258A) is enzymatically inactive. Myc-SRMS(WT) and Myc-SRMS(K258A) were expressed in SRMS KO U2OS cells. Cell lysates were immunoprecipitated with anti-Myc antibody and blotted with anti-p-Tyr antibody to detect SRMS autophosphorylation. (G) Myc-SRMS(WT) and Myc-SRMS(K258A) were transiently expressed in U2OS cells stably expressing GFP-LC3. Cells were fixed and stained with anti-Myc antibody to detect transfected cells. Representative images are shown. For quantitation, see Fig 1E. (H) MS/MS fragmentation data for human SRMS AA 374–387 sequence LLKDDIY(+79.97)SPSSSK, M/z 810.3703, z2, showing b/y ions. MS/MS fragment ions at M/z 941.42 (b7) and M/z 922.34 (y8) represent characteristic ions that unambiguously identify Y380 phosphorylation. The data underlying the figure can be found in S1 Data. IP, immunoprecipitation; KO, knockout; MEF, mouse embryonic fibroblast; MS/MS, tandem mass spectrometry; RNAi, RNA interference; SRMS, Src-related kinase lacking C-terminal regulatory tyrosine and N-terminal myristylation [file pbio.3001281.s001.pdf]

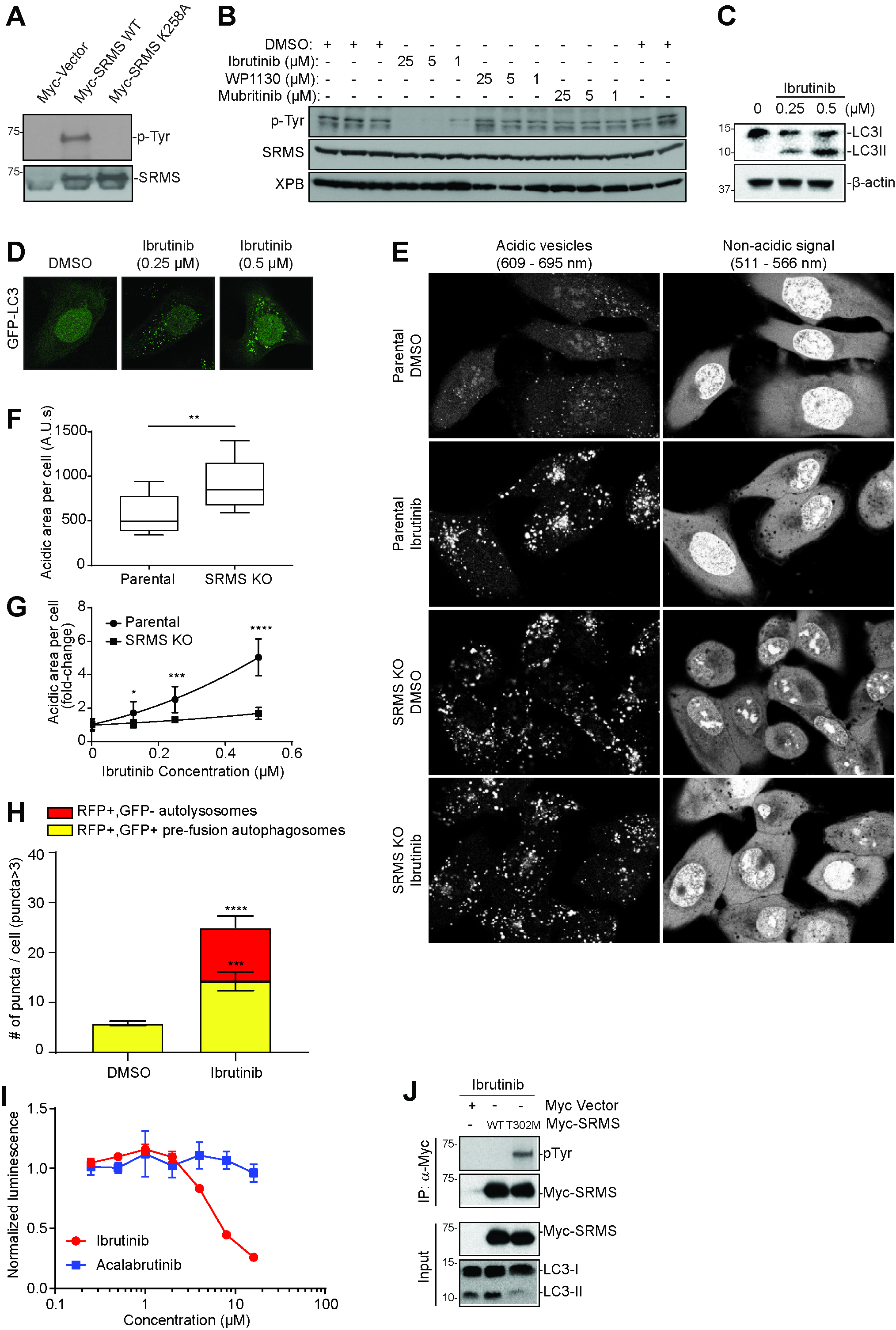

Supplement: S2 Fig — (A) SRMS overexpression increases p-Tyr immunoreactivity. HeLa cells were transiently transfected with the indicated constructs. Twenty-four hours later, lysates were collected and analyzed by western blot using the indicated antibodies. (B) Ibrutinib inhibits SRMS activity in a dose-dependent manner. HEK293 cells stably expressing Myc-SRMS(WT) were treated with the indicated compounds at the indicated concentrations for 2 hours. Cell lysates were subjected to immunoblotting with indicated antibodies. (C) Ibrutinib activates LC3 lipidation in a dose-dependent manner. Parental MDA-MB-231 cells were treated with ibrutinib at the indicated concentrations for 4 hours. Cell lysates were immunoblotted with anti-LC3 antibody. (D) Ibrutinib activates autophagosome formation in a dose-dependent manner. U2OS cells stably expressing GFP-LC3 were treated with ibrutinib at the indicated concentrations for 4 hours. GFP-LC3 puncta were detected by confocal microscopy. Representative images are shown. (E–G) Ibrutinib activates autophagy in an SRMS-dependent manner as measured by acridine orange. Parental or SRMS KO U2OS cells were treated with DMSO or ibrutinib (0.5 μM or as indicated) for 8 hours. Cells were then stained with 1 μg/mL acridine orange for 20 minutes and imaged at the indicated wavelengths. Representative images are shown (E) along with quantitation (F, G). For panel F, n = 10 images (123 cells) for parental and n = 8 images (130 cells) for SRMS KO. G shows mean +/− standard deviation of n = 10 images (123 cells), n = 11 images (131 cells), n = 8 images (100 cells), and n = 9 images (79 cells) for parental and n = 8 images (130 cells), n = 8 images (139 cells), n = 8 images (128 cells), and n = 9 images (181 cells) for SRMS KO (left to right). *p < 0.05, **p < 0.01, ***p < 0.001, ****p < 0.0001, t test. (H) Ibrutinib induces autophagosome biogenesis and autophagosome–lysosome fusion. U2OS cells stably expressing RFP-GFP-LC3 were treated with 1 μM ibrutinib for 12 hou [file pbio.3001281.s002.tif]

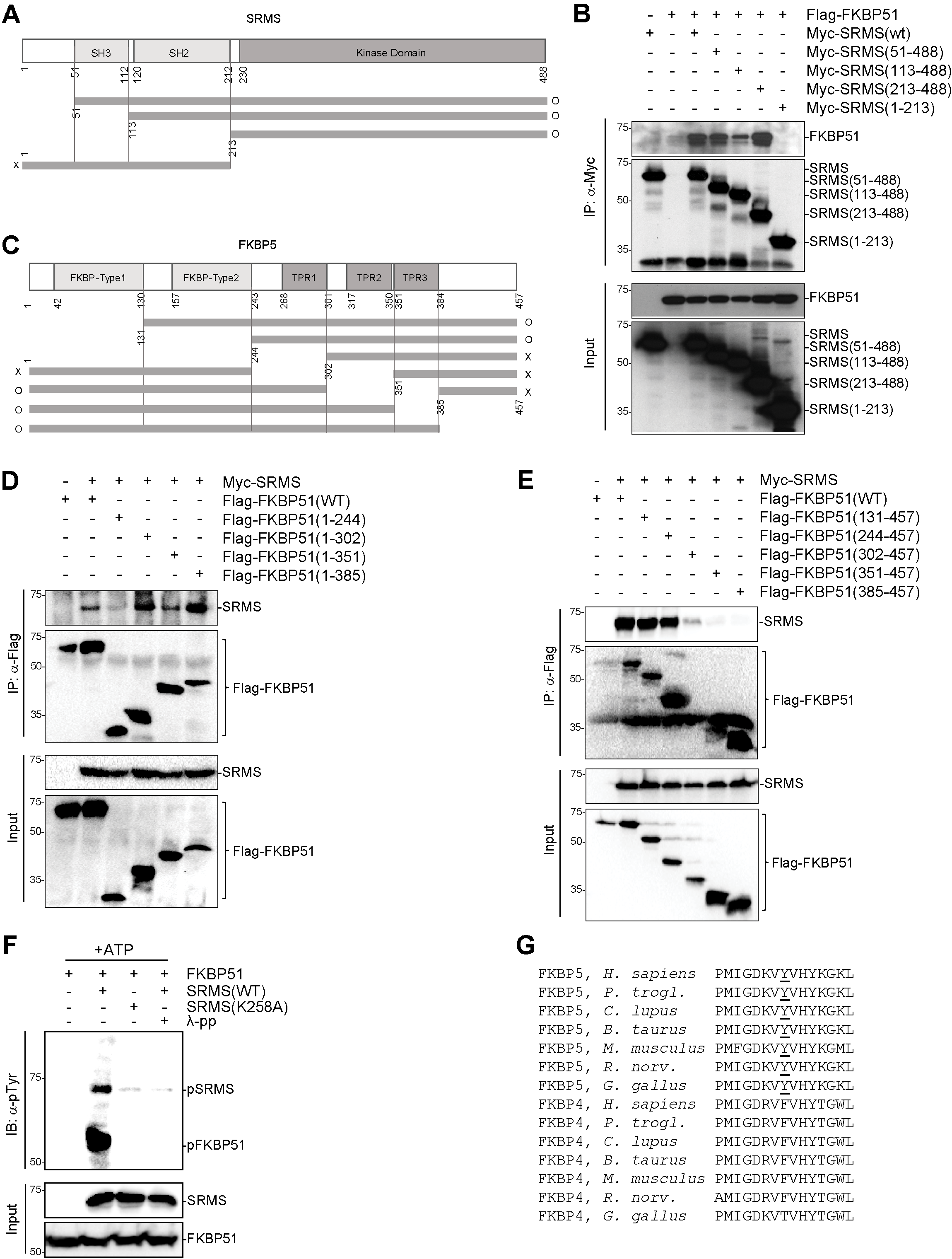

Supplement: S3 Fig — (A, B) SRMS interacts with FKBP51 through its kinase domain. SRMS truncated constructs (schematized in A) were transfected with Flag-FKBP51 in HEK293FT cells. Cell lysates were subjected to IP with anti-Myc and blotted with indicated antibodies (B). (C) FKBP51 interacts with SRMS through its TPR1 domain. Schematic representation of the 9 truncated FKBP51 constructs. (D, E) Each FKBP51 truncated construct was transfected with Myc-SRMS in HEK293FT cells. Cell lysates were subjected to anti-Flag IP and blotted with indicated antibodies. (F) SRMS directly phosphorylates FKBP51. An in vitro kinase assay was performed using GST-FKBP51 and in vitro transcribed/translated Myc-SRMS(WT) or Myc-SRMS(K258A) proteins, in presence or absence of λ-phosphatase as indicated. Tyrosine phosphorylation of FKBP51 was probed via anti-pTyr antibody, and GST-FKBP51 proteins were detected via anti-GST antibody. (G) Tyrosine 54 of FKBP5/FKBP51 is evolutionarily conserved, while the paralog FKBP4/FKBP52 has phenylalanine or threonine at the corresponding residue. FKBP51, FK506-binding protein 51; GST, glutathione S-transferase; IB, immunoblot; IP, immunoprecipitation; SRMS, Src-related kinase lacking C-terminal regulatory tyrosine and N-terminal myristylation sites; WT, wild-type. (TIF) [file pbio.3001281.s003.tif]

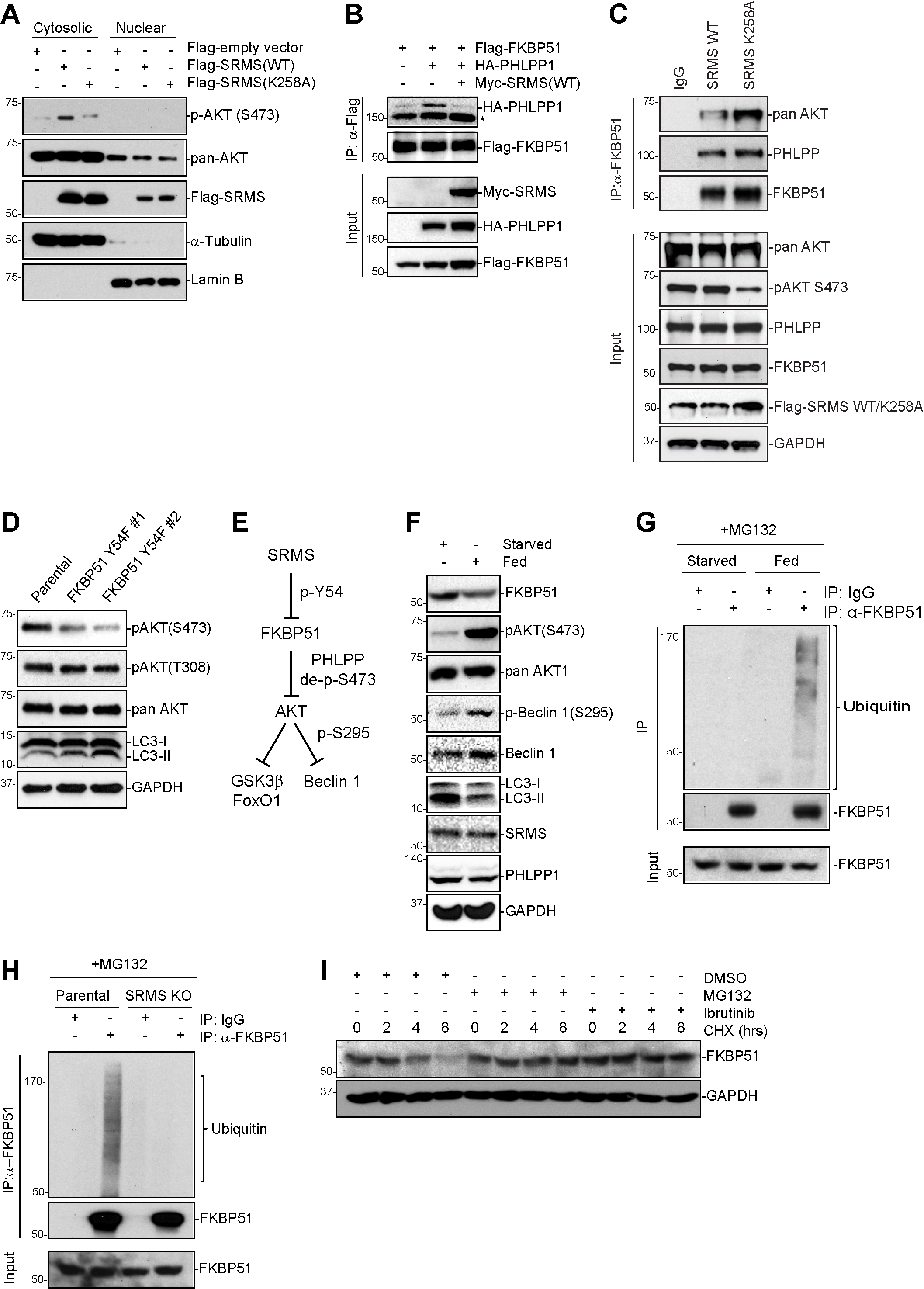

Supplement: S4 Fig — (A) SRMS promotes accumulation of cytosolic p-AKT(S473) in a kinase-dependent manner. SRMS KO U2OS cells were reconstituted with empty vector, Flag-SRMS(WT), or Flag-SRMS(K258A) as indicated. Lysates were collected, subjected to subcellular fractionation, and blotted with the indicated antibodies. Lamin B was used as a marker for nuclear fraction and α-tubulin for cytosolic fraction. (B) SRMS blocks the interaction between FKBP51 and PHLPP. FKBP51 was transfected with PHLPP and SRMS in 293T cells. Cell lysates were subjected to IP by anti-Flag antibody and blotted with indicated proteins. *nonspecific band. (C) WT SRMS or SRMS(K258A) mutant was expressed in SRMS KO U2OS cells. Lysates were collected and subjected to IP with anti-FKBP51 antibody or IgG control as indicated. Input (whole cell lysates) and immunoprecipitates were analyzed by western blot with the indicated antibodies. Note that FKBP51 interacts more strongly with AKT and PHLPP in cells expressing SRMS(K258A) compared to cells expressing WT SRMS. Also note that dephosphorylation of p-AKT(S473) is more prevalent in the whole cell lysate of cells expressing SRMS(K258A) mutant than WT SRMS. (D) U2OS cells in which all endogenous FKBP51 alleles were converted to the nonphosphorylatable FKBP51 Y54F mutant by CRISPR/Cas9 exhibit decreased accumulation of p-AKT at Ser 473 and increased LC3 lipidation relative to parental U2OS cells. Cell lysates were immunoblotted with the indicated antibodies. (E) The proposed signaling pathway through which SRMS promotes AKT signaling and restrains autophagy. (F) Nutrient-dependent FKBP51 reduction is not unique to MDA-MB-231 cells. MDA-MB-435 cells were incubated under starved or fed conditions for 4 hours. Cell lysates were immunoblotted with indicated antibodies. (G) FKBP51 is poly-ubiquitinated under fed condition. MDA-MB-231 cells were incubated under starved or fed conditions and treated with 10 nM MG132 for 4 hours. Cell lysates were subjected to IP by anti-FKBP51 ant [file pbio.3001281.s004.tif]

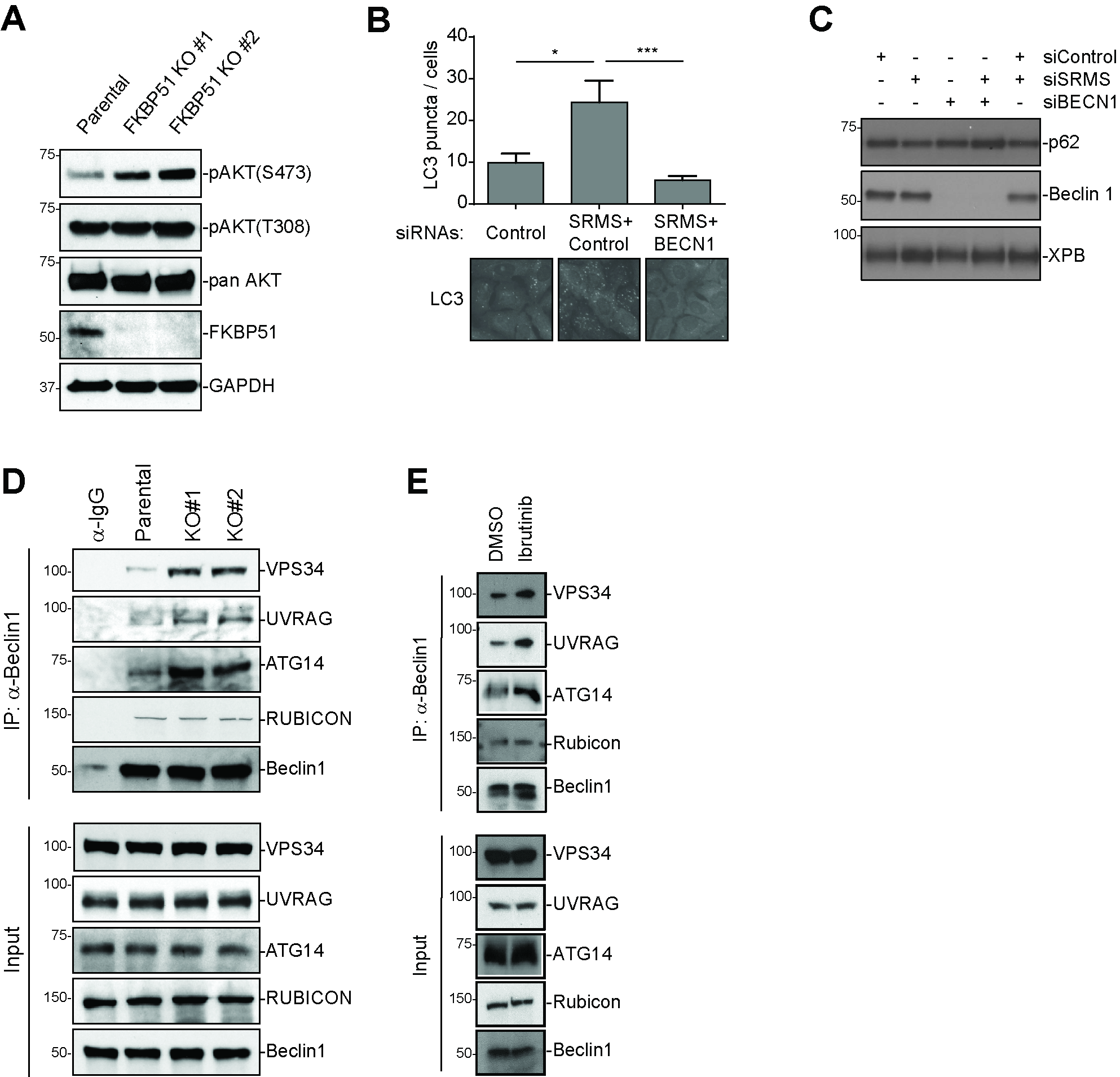

Supplement: S5 Fig — (A) KO of FKBP51 increases abundance of p-AKT at Ser 473. Lysates were analyzed by western blot with the indicated antibodies. (B, C) SRMS restrains Beclin 1-dependent autophagy. Parental U2OS cells were transfected with the indicated siRNA oligonucleotides alone or in combination, as indicated. Seventy-two hours later, the cells were fixed and stained with anti-LC3 antibody for imaging (B) or lysed and processed for western blot with the indicated antibodies (C). From left to right, mean +/− standard deviation of n = 3 images (60 cells), n = 3 images (48 cells), and n = 4 images (113 cells) are shown. *p < 0.1, ***p < 0.001, t test. (D) Parental and SRMS KO MDA-MB-231 cells were lysed and subjected to IP with anti-Beclin 1 antibody or control anti-IgG as indicated. Input (whole cell lysates) and immunoprecipitates were subjected to western blot analysis with the indicated antibodies. Note that Beclin 1 interacts more strongly with VPS34, UVRAG, and ATG14 (but not Rubicon) in the SRMS KO cells relative to parental control cells. (E) Parental MDA-MB-231 cells were treated with DMSO or ibrutinib (0.5 μM concentration for 4 hours). Anti-Beclin 1 IP was performed, and both input (whole cell lysates) and immunoprecipitates were analyzed by western blot with the indicated antibodies. Note that Beclin 1 interacts more strongly with VPS34, UVRAG, and ATG14 (but not Rubicon) in cells treated with ibrutinib relative to DMSO control. The data underlying the figure can be found in S1 Data. FKBP51, FK506-binding protein 51; GAPDH, glyceraldehyde-3-phosphate dehydrogenase; IgG, immunoglobulin G; IP, immunoprecipitation; KO, knockout; siRNA, small interfering RNA; SRMS, Src-related kinase lacking C-terminal regulatory tyrosine and N-terminal myristylation sites. (TIF) [file pbio.3001281.s005.tif]

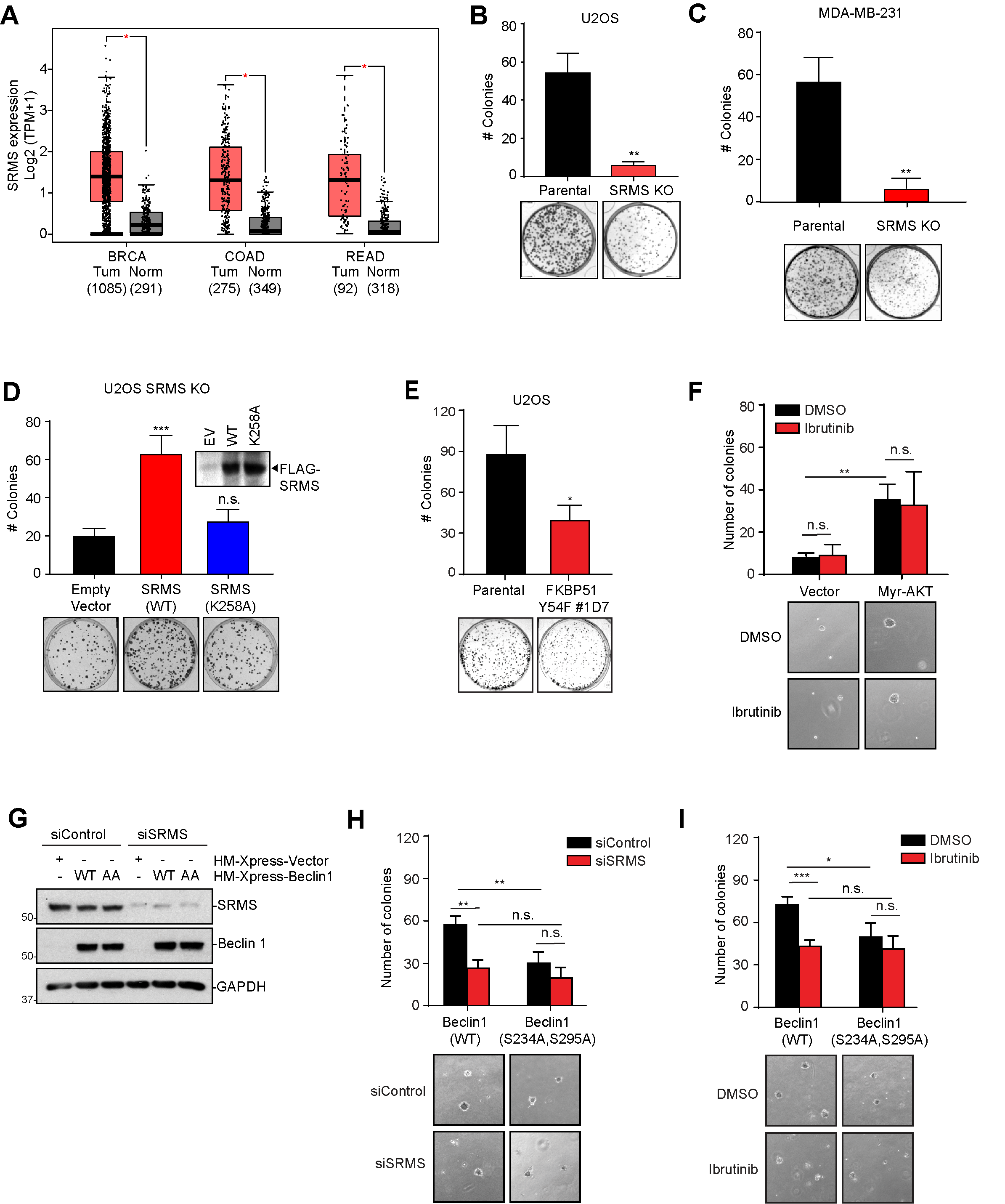

Supplement: S6 Fig — (A) SRMS expression is elevated in human breast and colorectal tumors (red) relative to corresponding normal tissues (gray). *p < 0.05. (B) SRMS supports clonogenic growth of U2OS osteosarcoma cells. Parental and SRMS KO U2OS cells were plated sparsely and allowed to grow for 12 days. Colonies were stained with crystal violet, imaged, and counted. Mean + standard deviation of n = 3 replicates is shown along with representative images. **p < 0.01, t test. (C) SRMS supports clonogenic growth of MDA-MB-231 triple-negative breast cancer cells. Parental or SRMS KO MDA-MB-231 cells were seeded sparsely in 6-well plates and allowed to grow for 17 days. Colonies were stained with crystal violet, imaged, and counted. Mean + standard deviation of n = 3 replicates is shown along with representative images. **p < 0.01, t test. (D) The ability of WT SRMS vs. kinase-dead SRMS to rescue the clonogenic growth defect of SRMS KO U2OS cells were compared. Mean + standard deviation of n = 3 replicates is shown along with representative images. ns p > 0.05, ***p < 0.001, t test. (Relates to Fig 6C). (E) CRISPR-Cas9-mediated mutation of all FKBP51 alleles to nonphosphorylatable FKBP51 Y54F mutant into U2OS cells impairs growth. Clonogenic growth was compared between parental and FKBP51 Y54F edited cells. Mean + standard deviation of n = 3 replicates is shown along with representative images. *p < 0.05. (F) Constitutively active AKT rescues the growth defect of SRMS KO cells without conferring sensitivity to ibrutinib. EV or Myr-AKT was transfected in SRMS KO MDA-MB-231 cells. Cells were sparsely seeded in soft agar and treated with DMSO or 0.5 μM ibrutinib for 20 days. Mean + standard deviation of n = 3 replicates is shown along with representative images. ns p > 0.05, **p < 0.01, t test. (G–I) SRMS antagonizes the tumor suppressive function of WT Beclin 1 but not of Beclin 1 (S243A, S295A), which is not phosphorylated by AKT [15]. EV, Beclin 1 (WT), or Beclin 1 (S234A, S295A) were expre [file pbio.3001281.s006.tif]

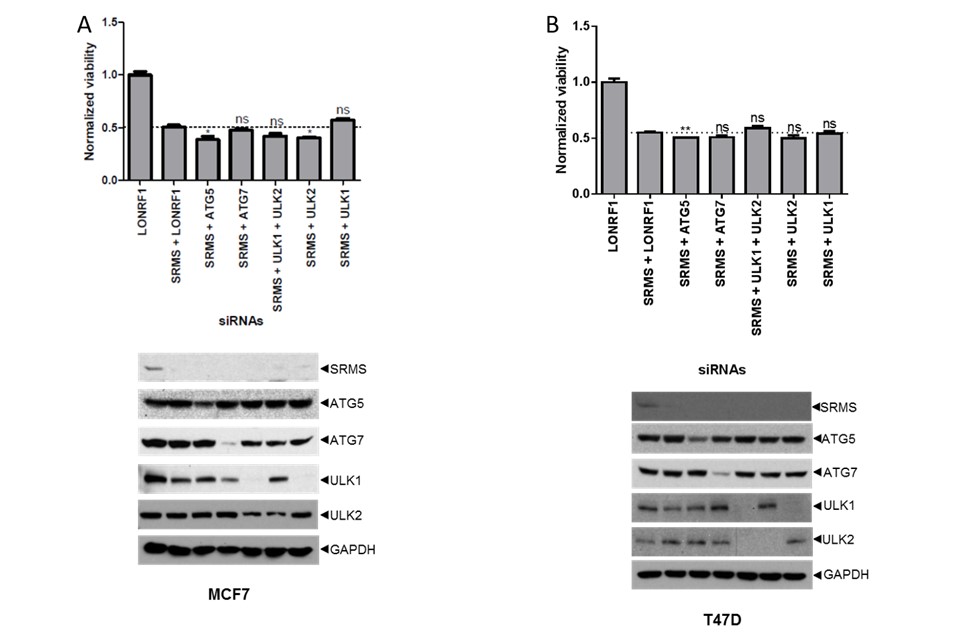

Supplement: S7 Fig — (A) MCF7 breast cancer cells were transfected with the indicated siRNA oligonucleotides alone or in combination in a standard 96-well proliferation assay. siLONRF1 served as a negative control. Seventy-two hours later, growth was measured by Cell Titer Glo assay and normalized to siLONRF1 control (top), and efficiency of knockdown was measured by western blot (bottom). (B) The same experiment as described in (A) was performed using T47D breast cancer cells. Means + standard deviation of n = 3 replicates are shown; ns p > 0.05, *p < 0.05, **p < 0.01, t test. The data underlying the figure can be found in S1 Data. GAPDH, glyceraldehyde-3-phosphate dehydrogenase; ns, non-significant (p>0.05); siRNA, small interfering RNA; SRMS, Src-related kinase lacking C-terminal regulatory tyrosine and N-terminal myristylation sites. (JPG) [file pbio.3001281.s007.jpg]

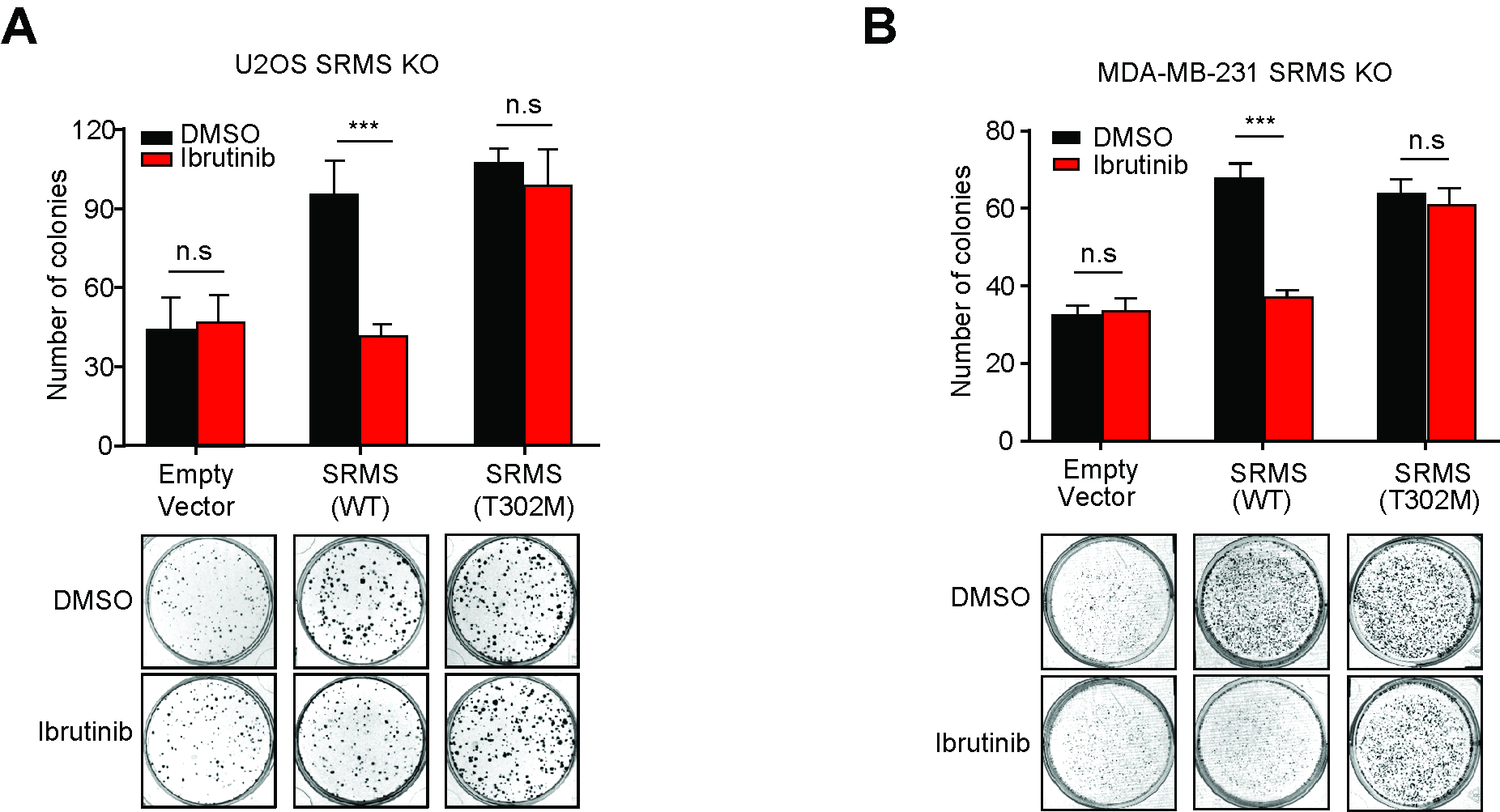

Supplement: S8 Fig — (A) The ability of WT SRMS vs. SRMS(T302M) to rescue the clonogenic growth defect and ibrutinib resistance of SRMS KO U2OS cells was compared. Mean + standard deviation of n = 3 replicates is shown along with representative images. ***p < 0.001, t test. (B) The ability of WT SRMS vs. SRMS(T302M) to rescue the clonogenic growth defect and ibrutinib resistance of SRMS KO MDA-MB-231 cells was compared. Mean + standard deviation of n = 3 replicates is shown along with representative images. n.s, ***p < 0.001, t test. The data underlying the figure can be found in S1 Data. KO, knockout; n.s, non-significant (p>0.05); SRMS, Src-related kinase lacking C-terminal regulatory tyrosine and N-terminal myristylation sites; WT, wild-type. (TIF) [file pbio.3001281.s008.tif]
