# Supplementary material for: The nonreceptor tyrosine kinase SRMS inhibits autophagy and promotes tumor growth by phosphorylating the scaffolding protein FKBP51
Source: PLoS Biol. 2021 Jun 2;19(6):e3001281. doi: 10.1371/journal.pbio.3001281 (PMC8202955; doi:10.1371/journal.pbio.3001281)
Supplement: S1 Raw Images — (PDF) [file pbio.3001281.s011.pdf]

Fig1F

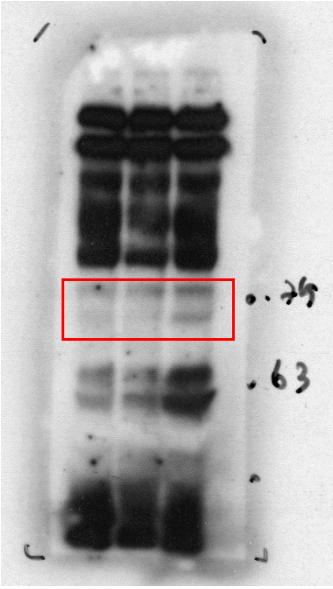

p-Tyr

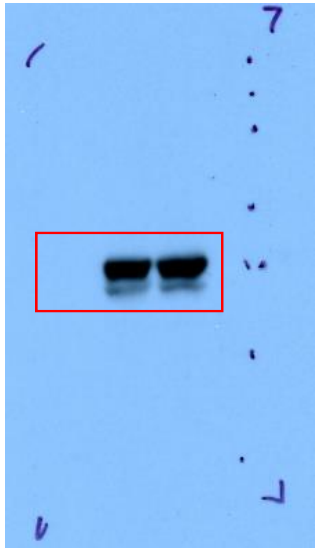

SRMS

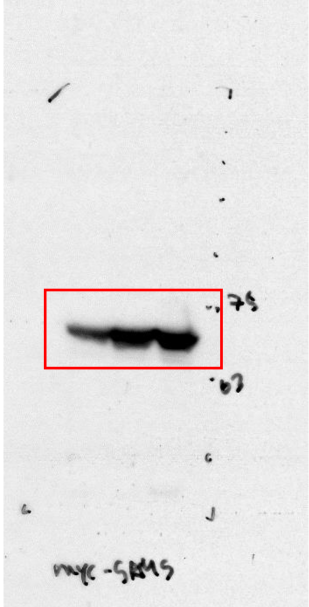

SRMS

Fig2A

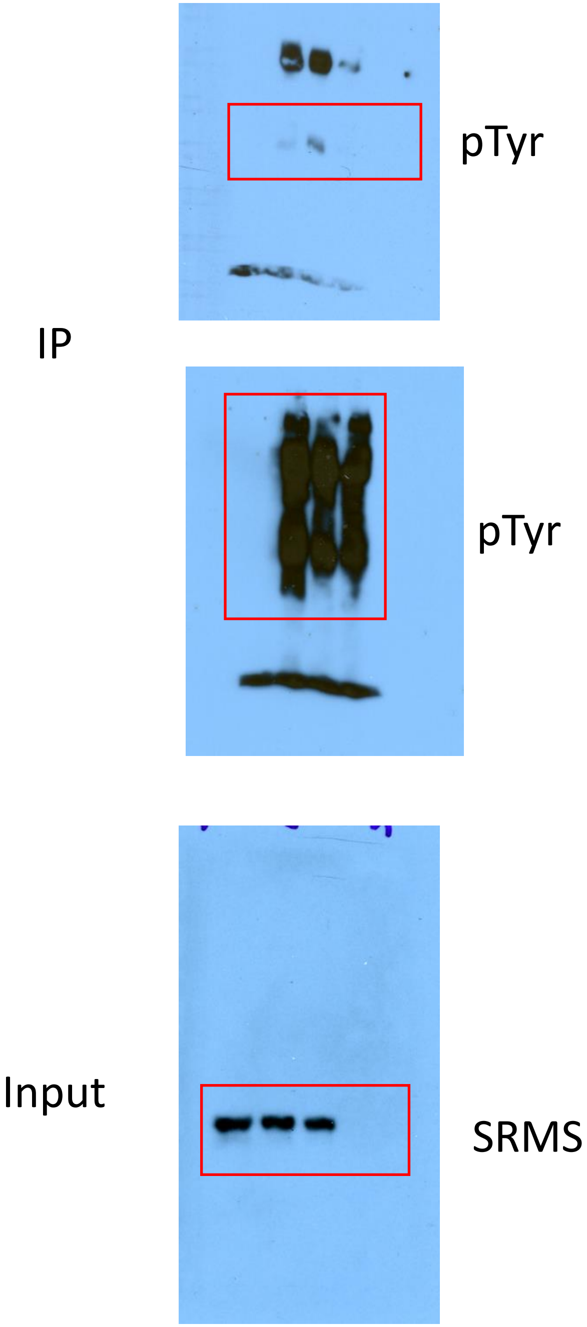

Fig2E

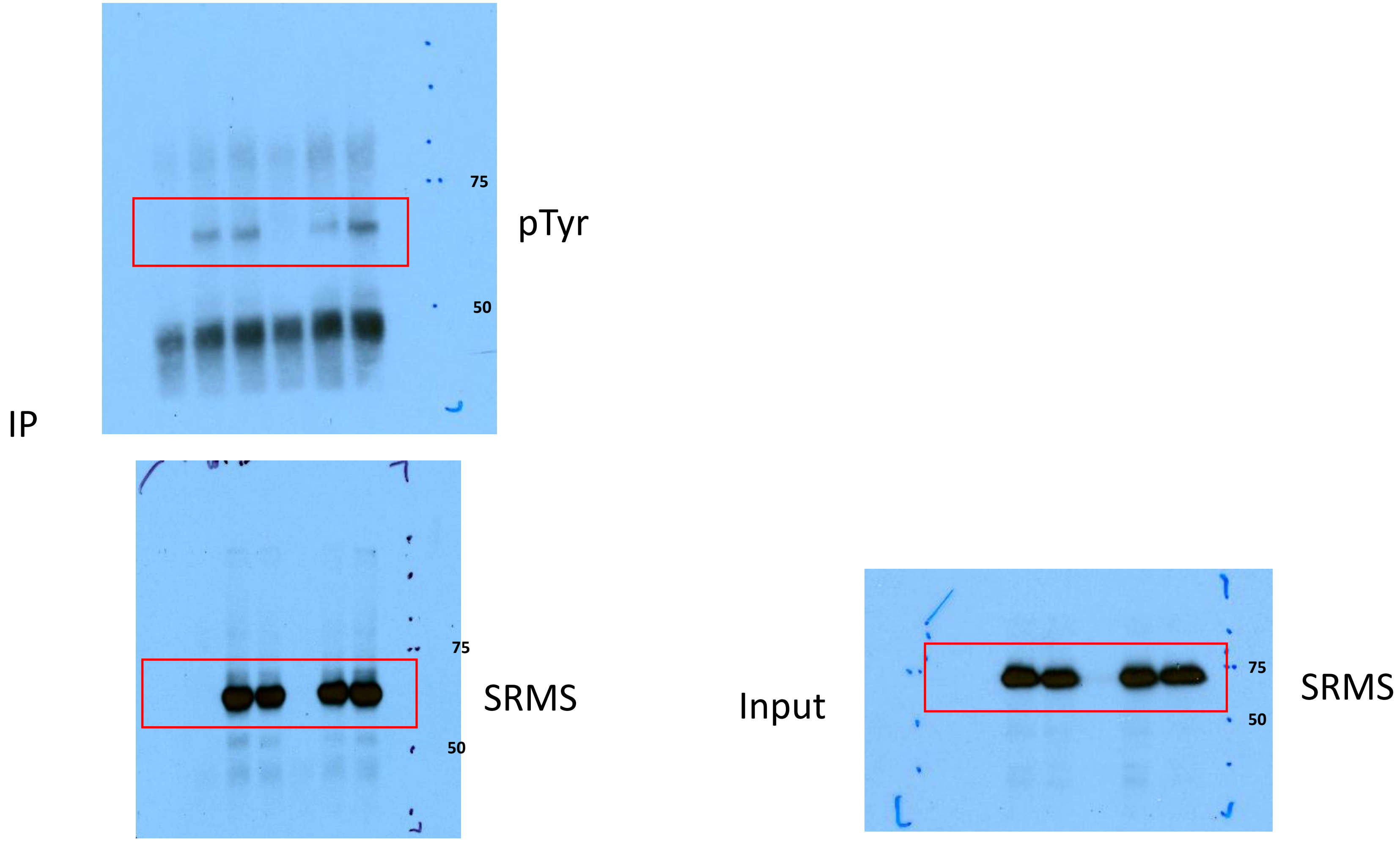

Fig3B

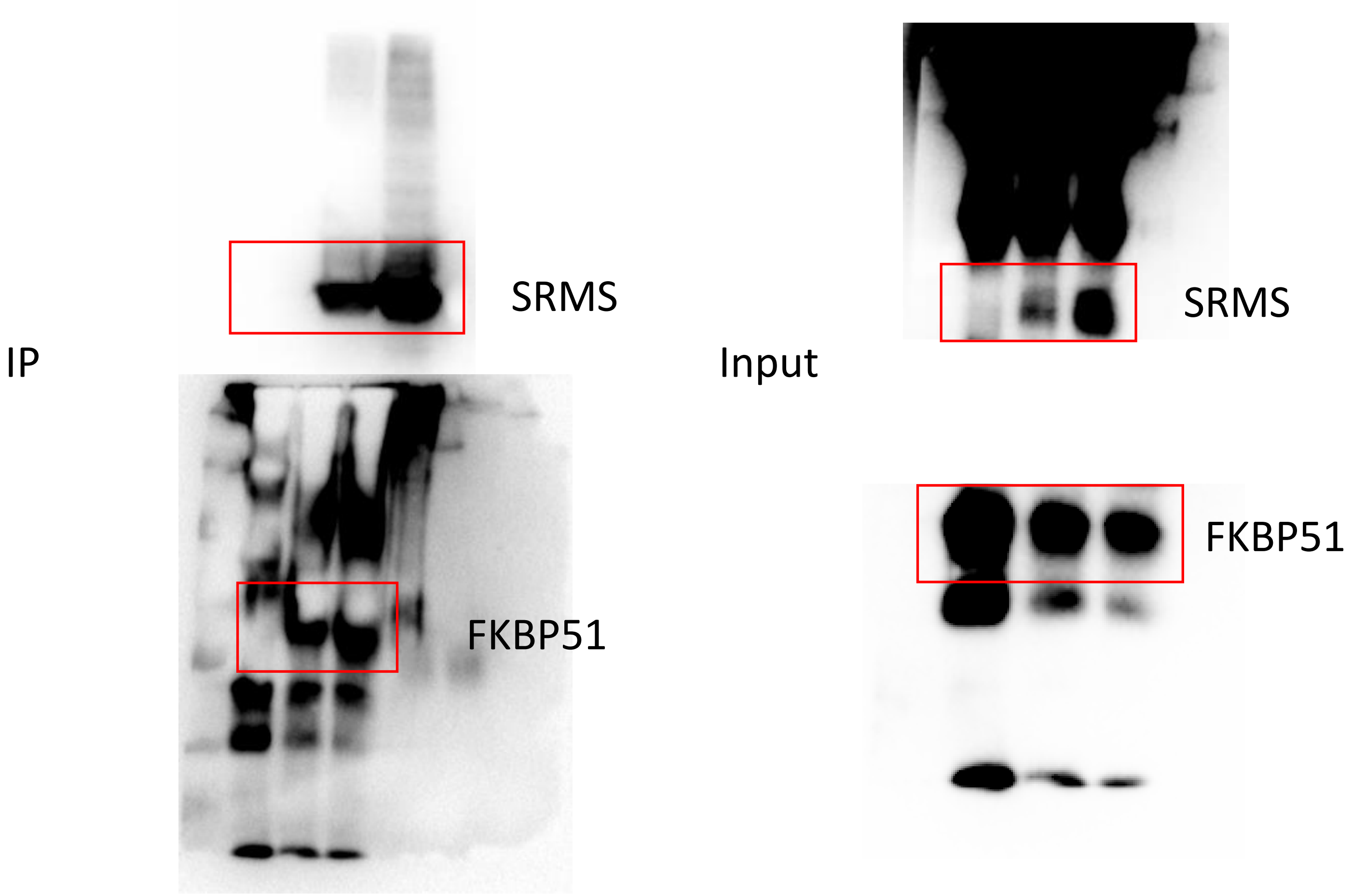

Fig3C

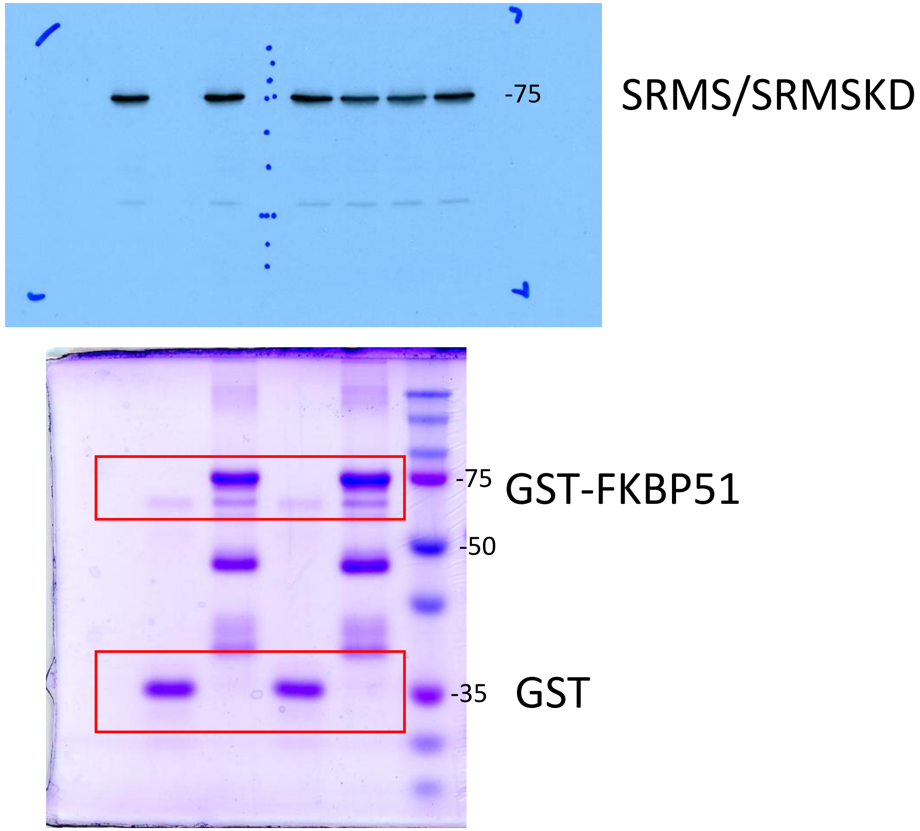

Fig3D

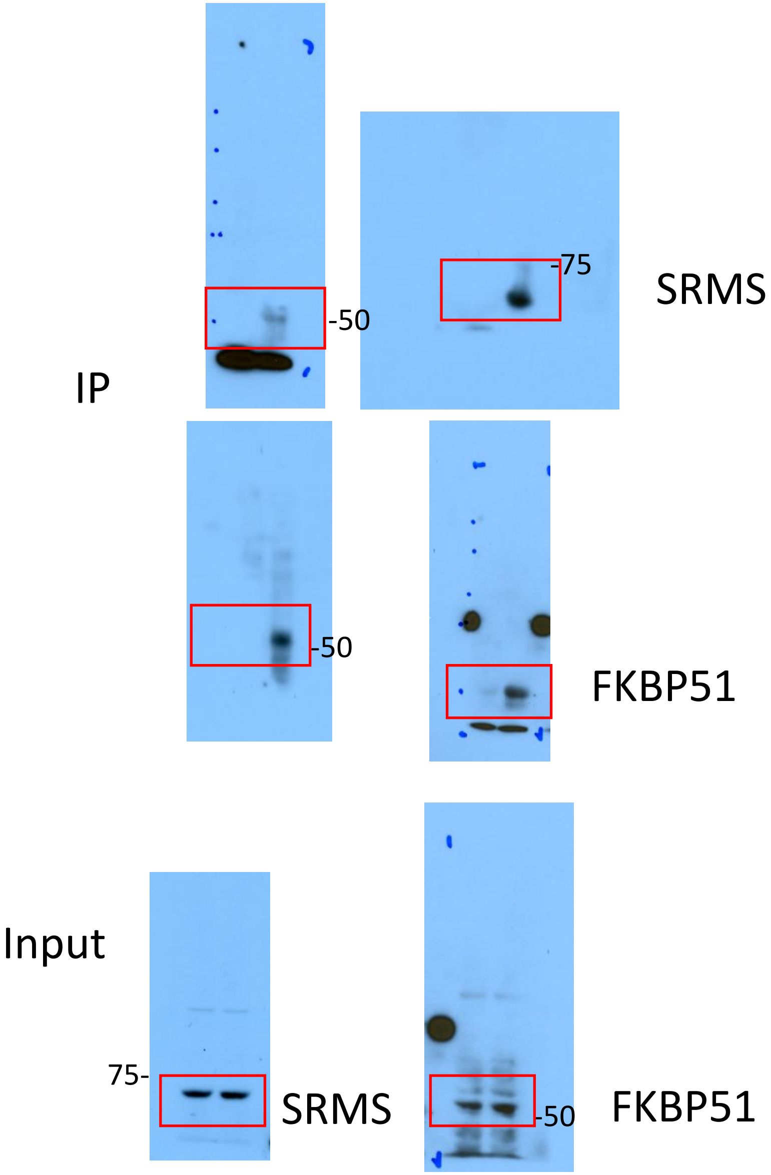

Fig3E

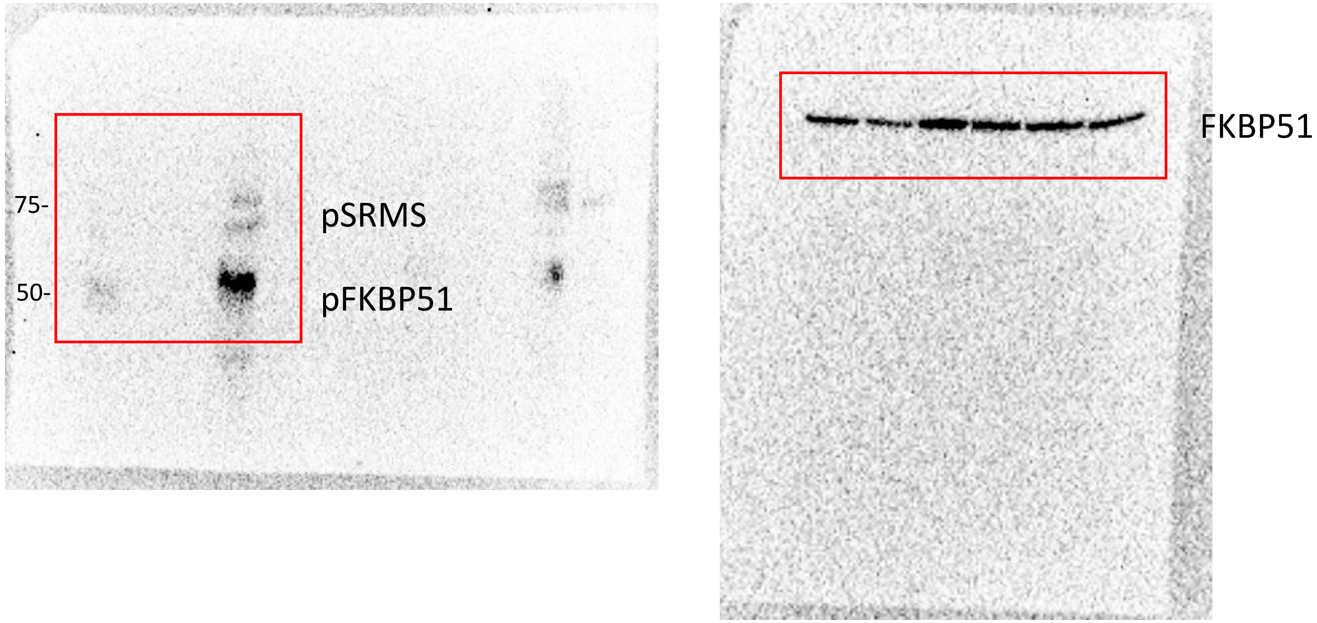

Fig3F

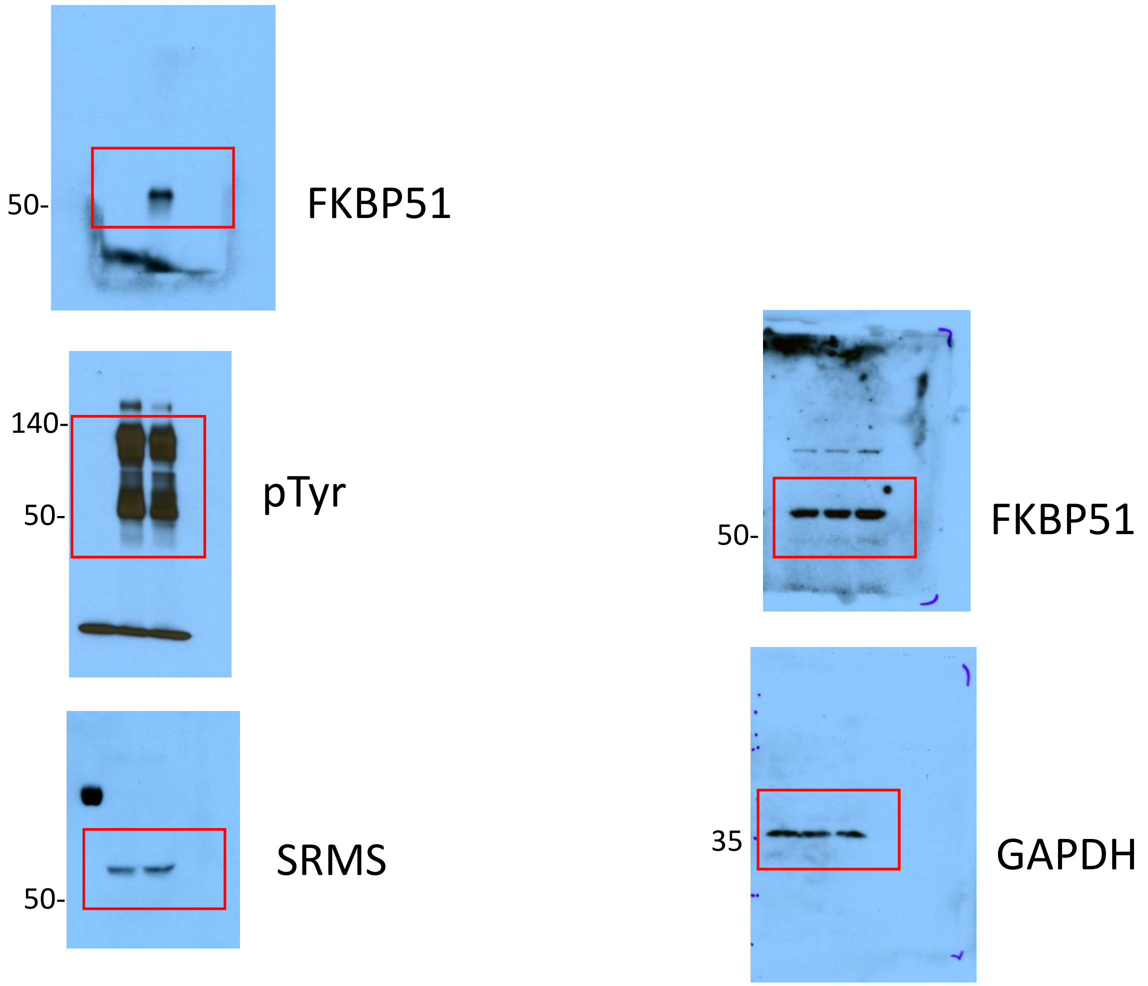

Fig3G

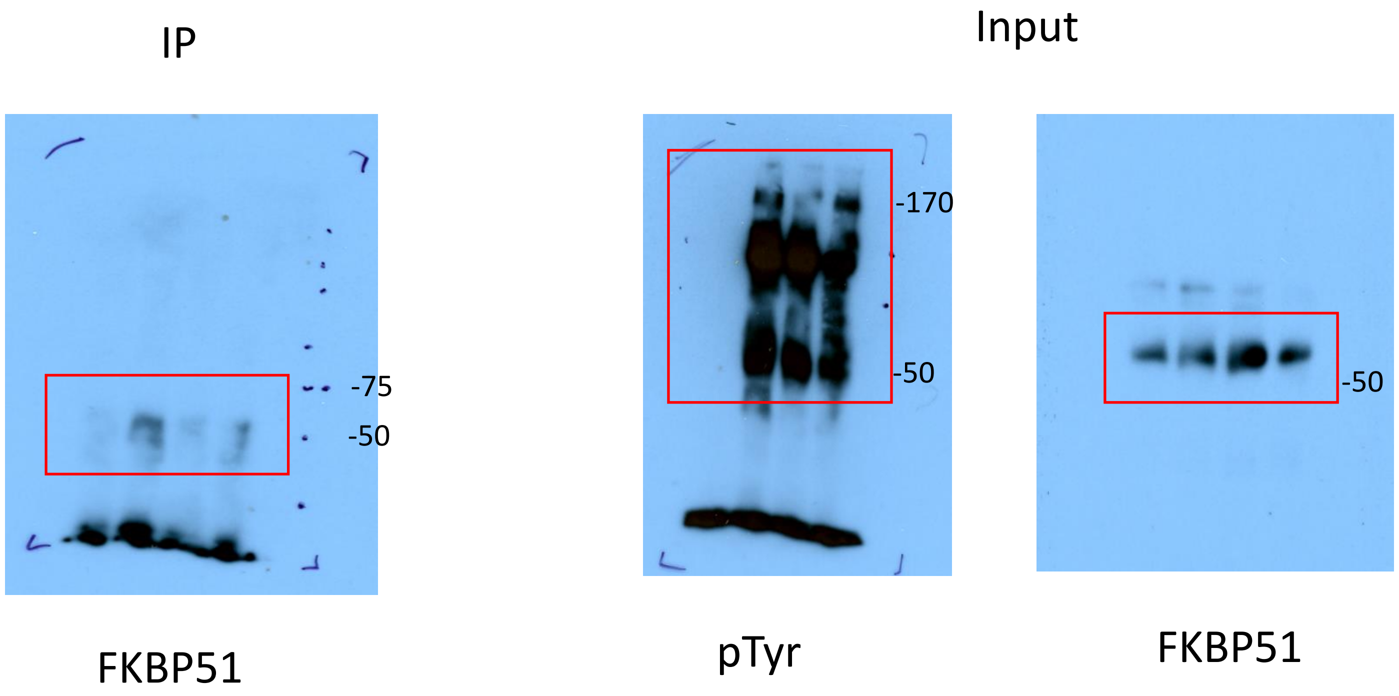

Fig3H

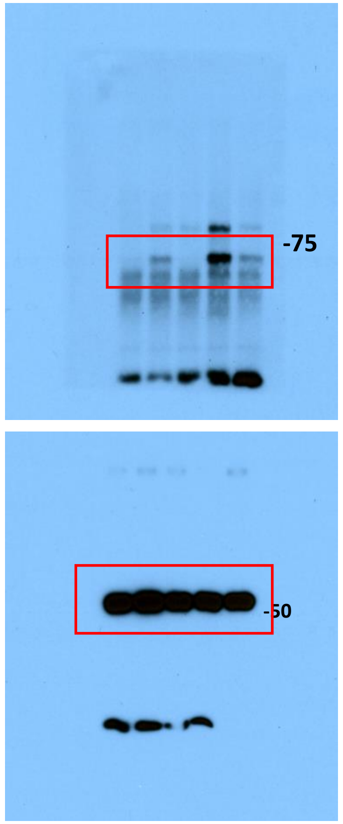

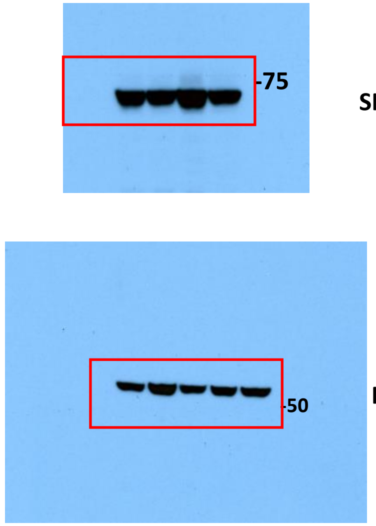

Fig4A

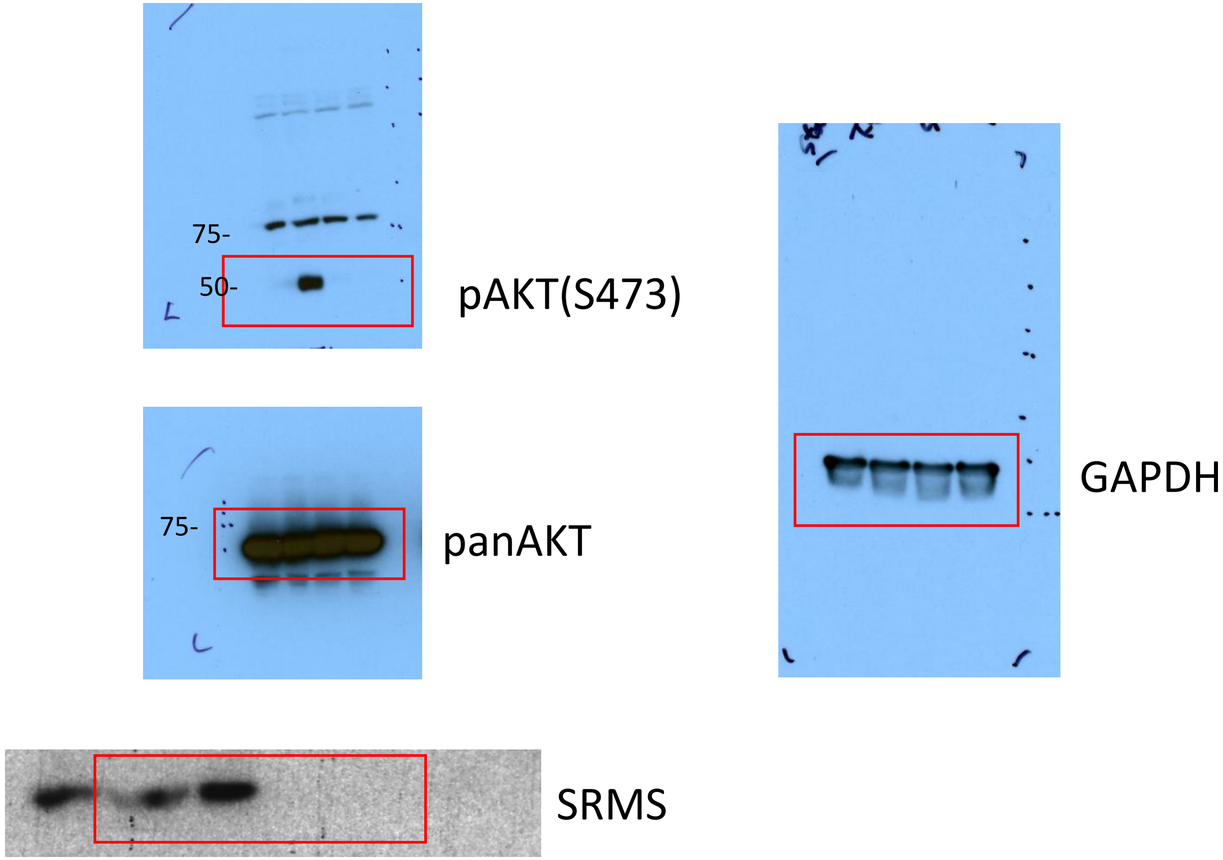

Fig4B

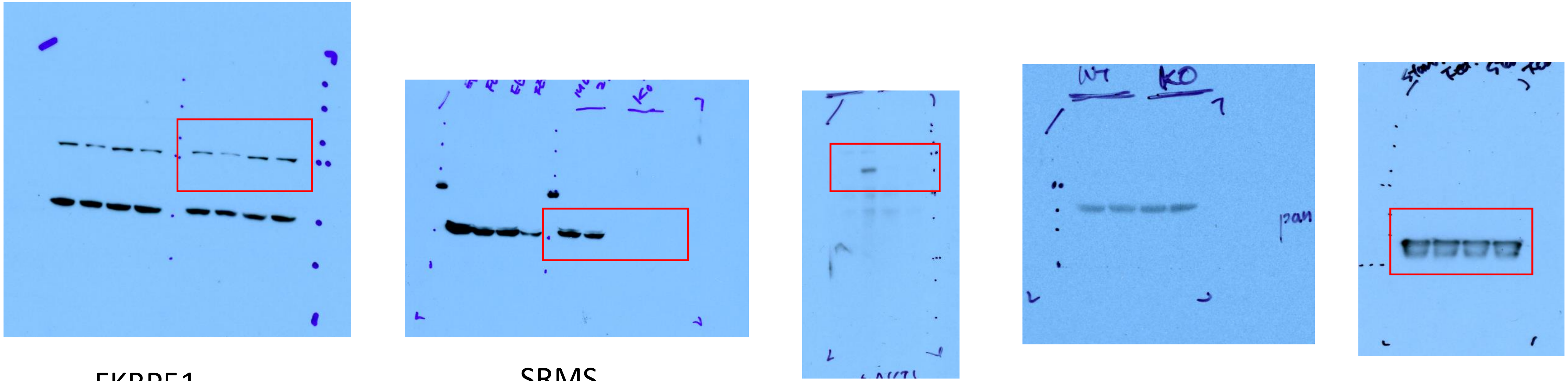

FKBP51 SRMS p-AKT(S473) pan AKT GAPDH

Fig4C

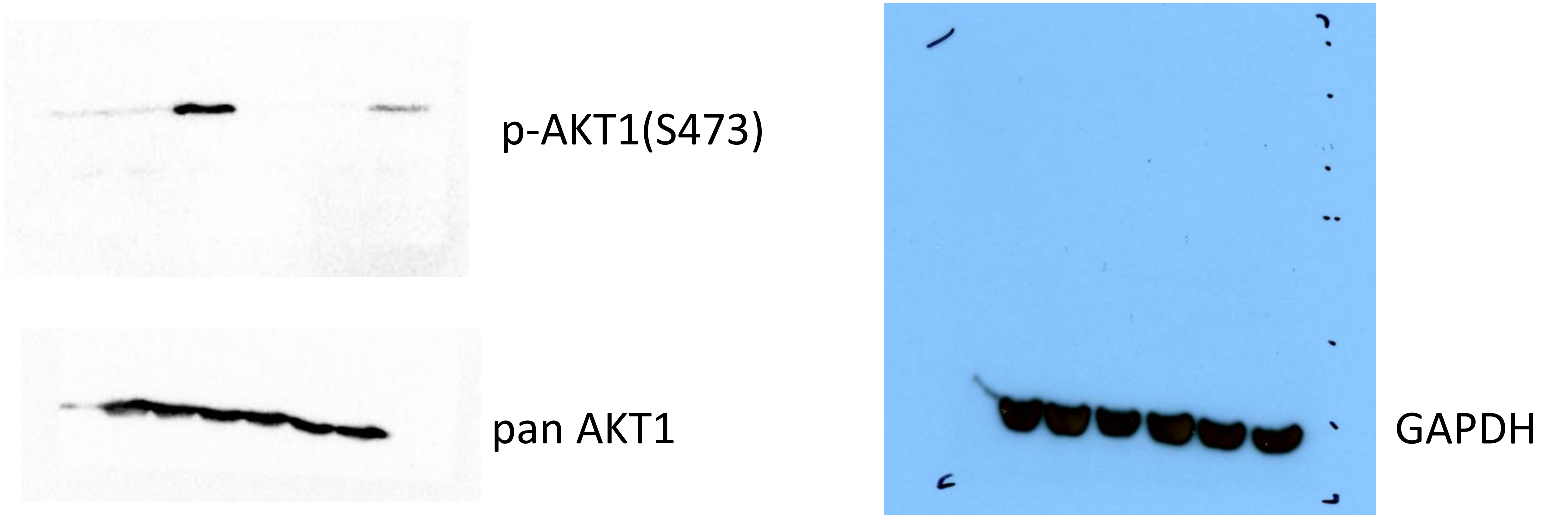

Fig4D

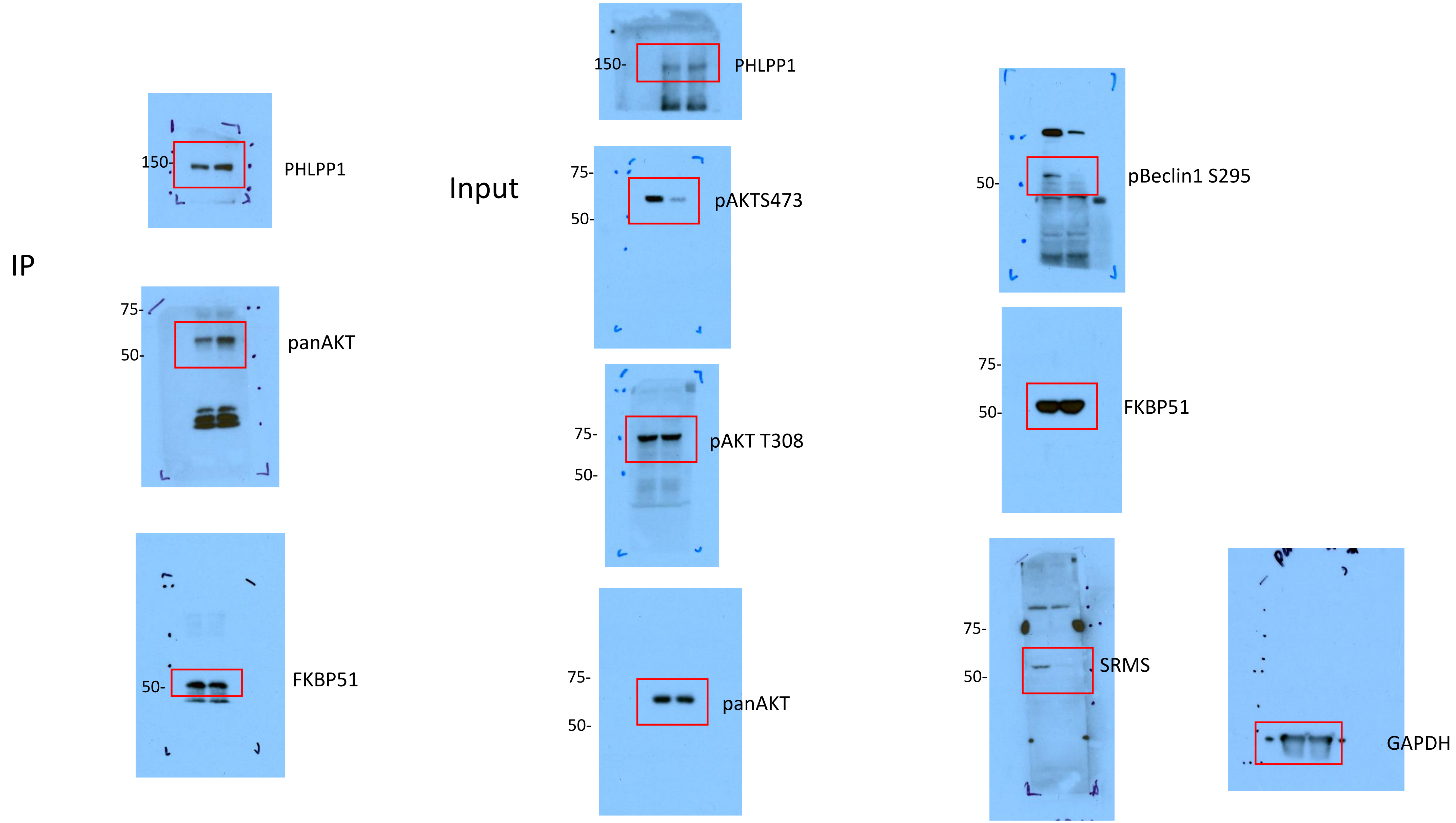
IP
150-
PHLPP1
75-
50-
panAKT
50-
FKBP51
Input
150-
PHLPP1
75-
50-
pAKTS473
75-
50-
pAKT T308
75-
50-
panAKT
50-
pBeclin1 S295
75-
50-
FKBP51
75-
50-
SRMS
GAPDH

Fig4E

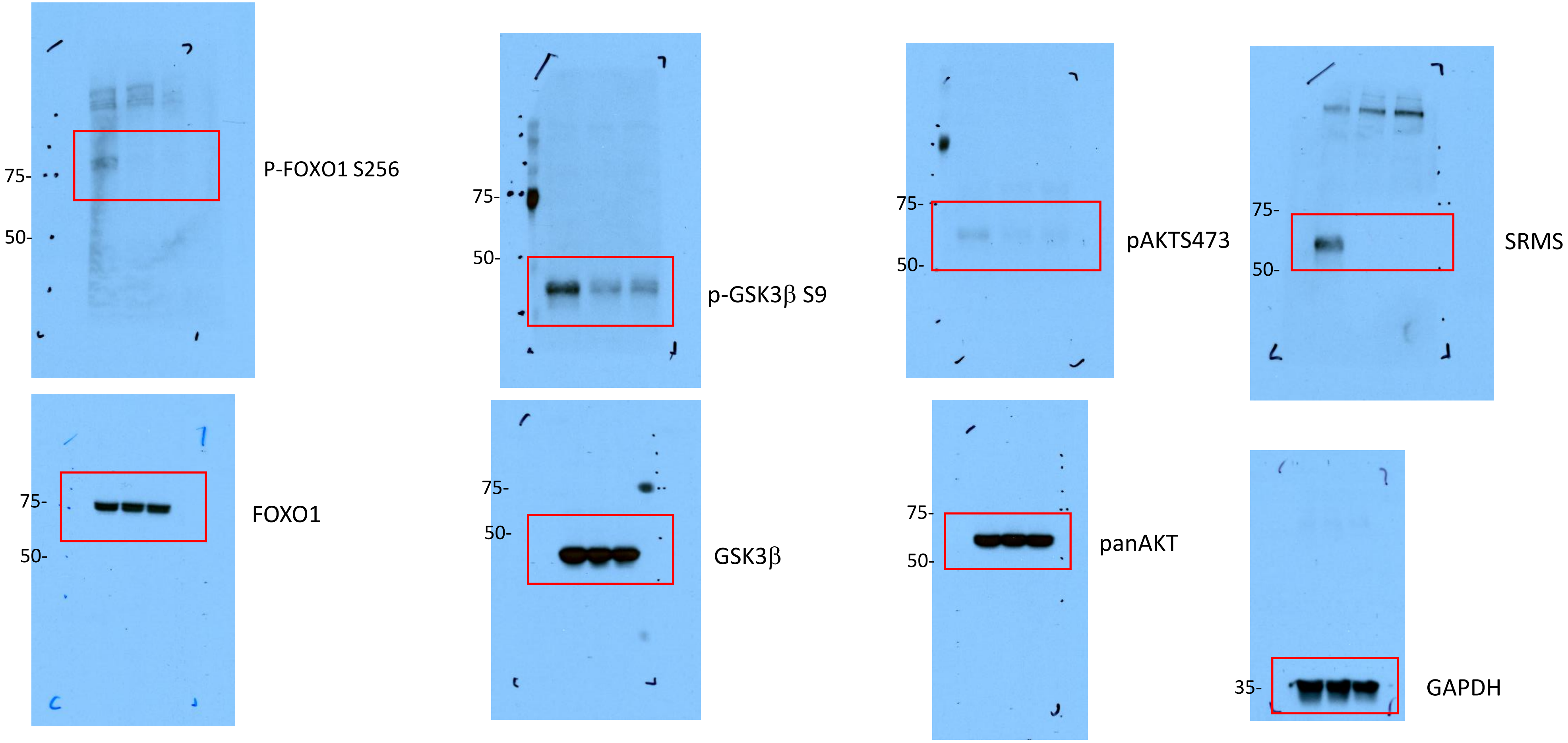
P-FOXO1 S256
75-
50-
p-GSK3β S9
75-
50-
pAKTS473
75-
50-
SRMS
75-
50-
FOXO1
75-
50-
GSK3β
75-
50-
panAKT
75-
50-
GAPDH
35-

Fig4F

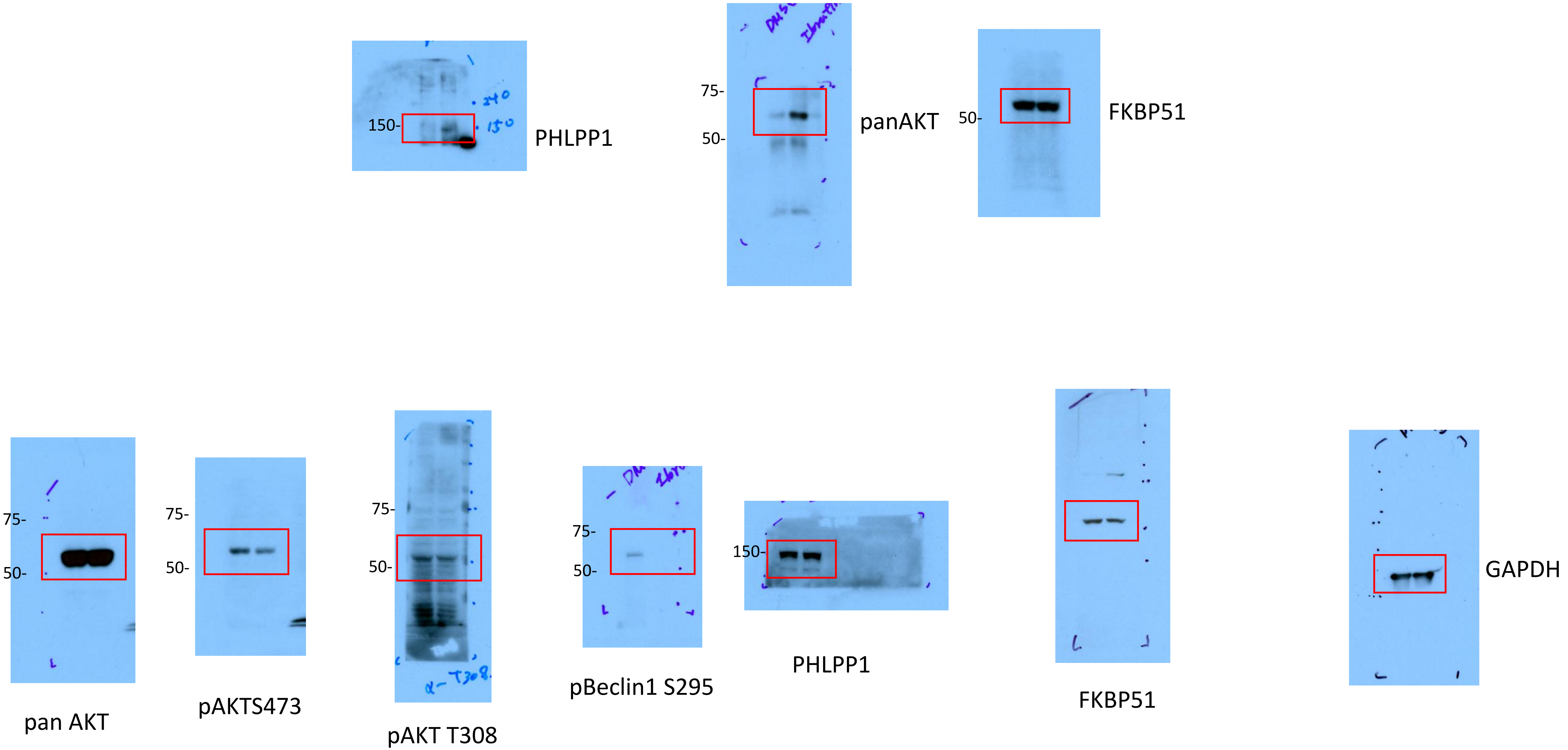
150-
PHLPP1
75-
50-
panAKT
50-
FKBP51
75-
50-
pan AKT
75-
50-
pAKTS473
75-
50-
pAKT T308
75-
50-
pBeclin1 S295
150-
PHLPP1
FKBP51
GAPDH

Fig4G

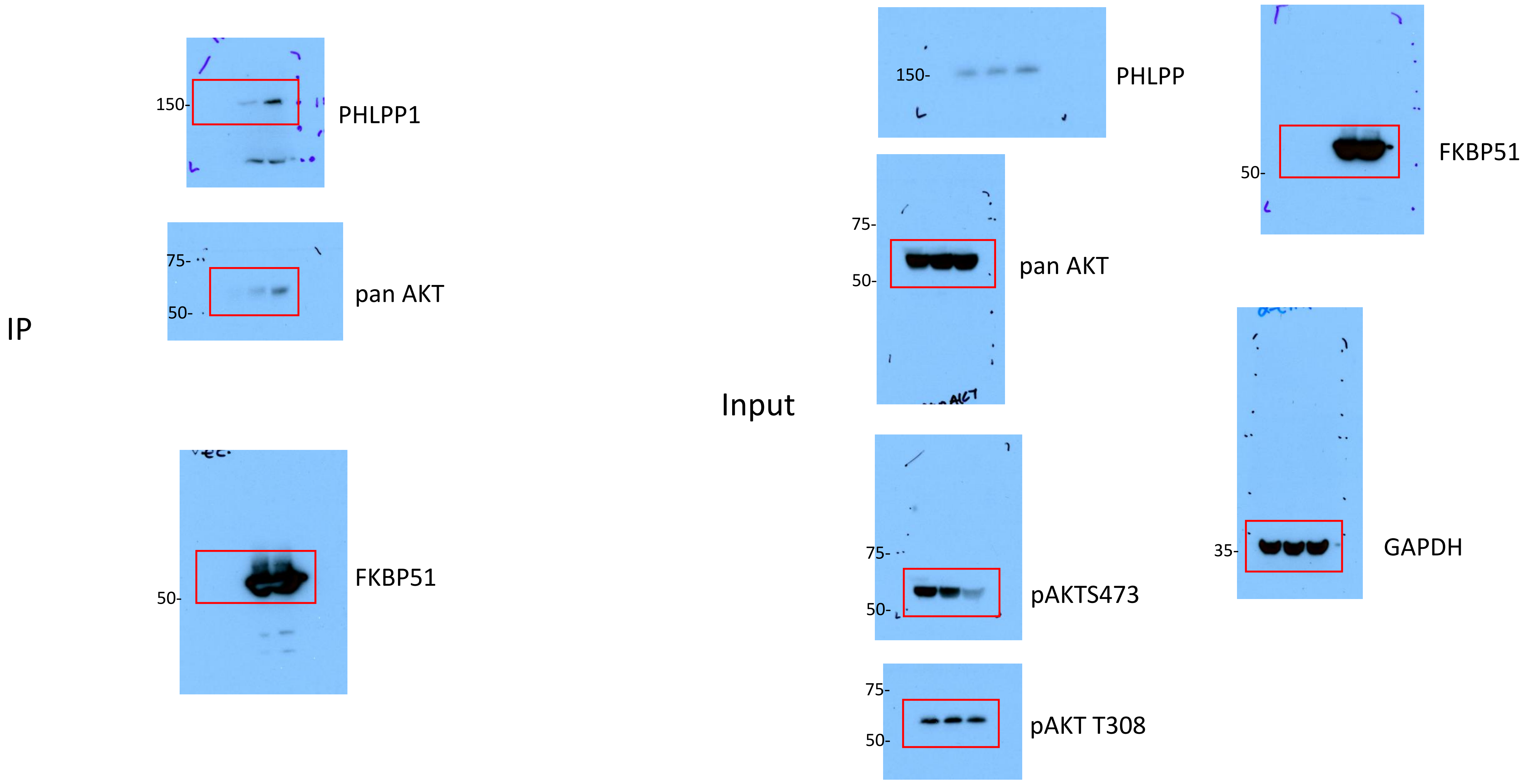

IP
150-
PHLPP1
75-
50-
pan AKT
50-
FKBP51
Input
150-
PHLPP
75-
50-
pan AKT
75-
50-
pAKTS473
75-
50-
pAKT T308
50-
FKBP51
35-
GAPDH

Fig5A

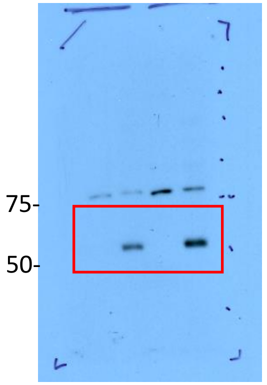

pAKTS473

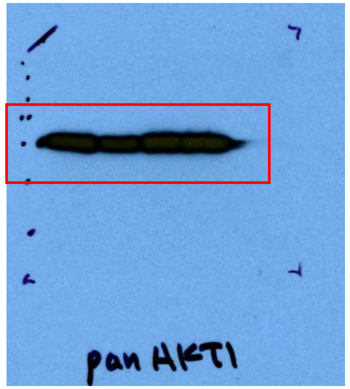

panAKT

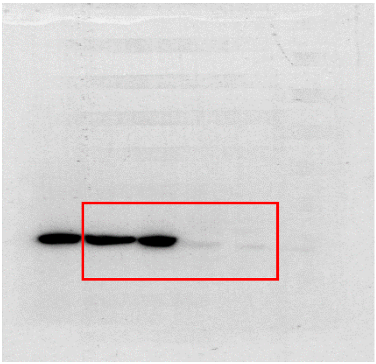

FKBP51

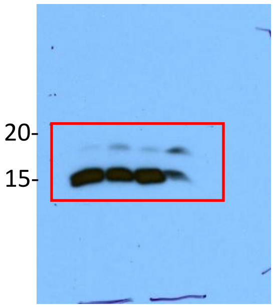

LC3I/II

Fig5C

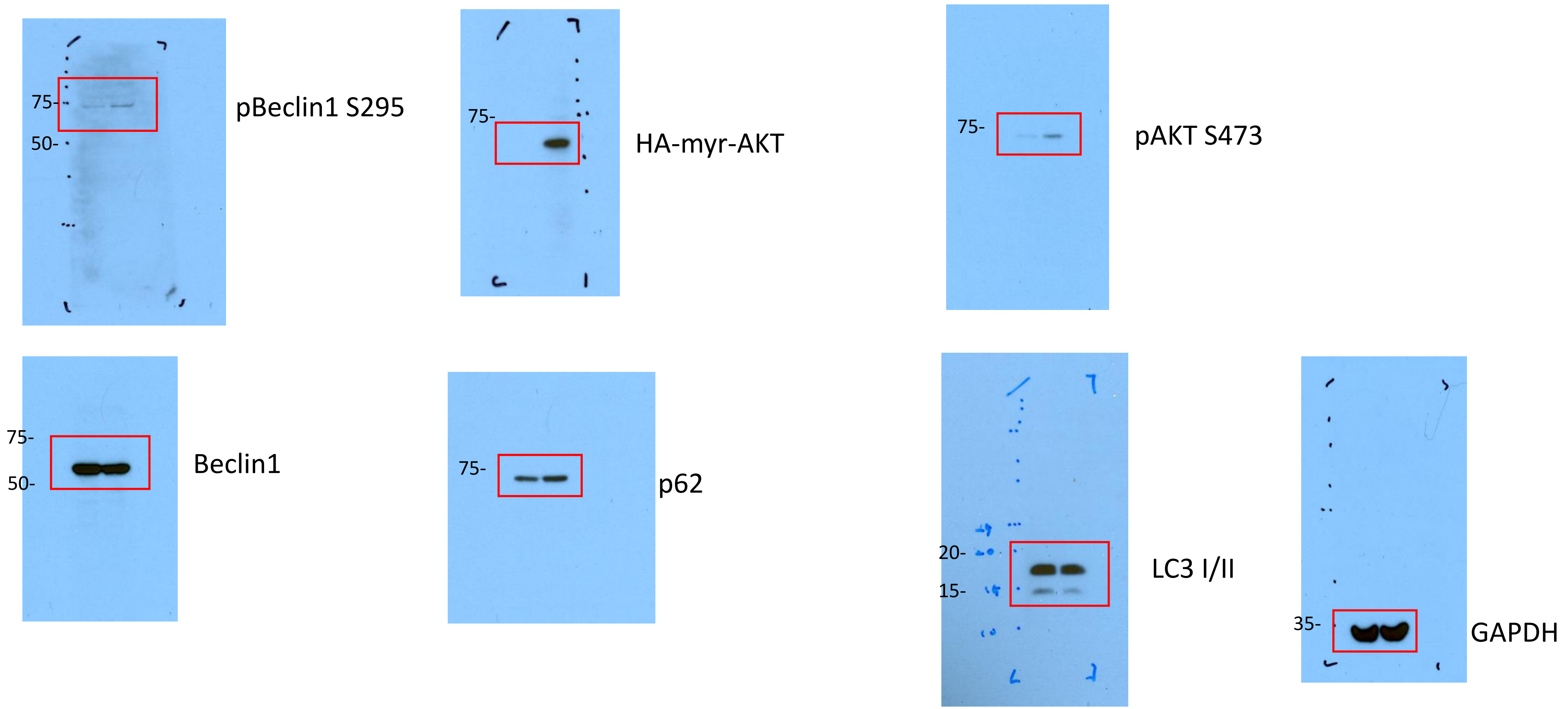

75-
50-
pBeclin1 S295
75-
HA-myr-AKT
75-
pAKT S473
75-
50-
Beclin1
75-
p62
20-
15-
LC3 I/II
35-
GAPDH

Fig5D

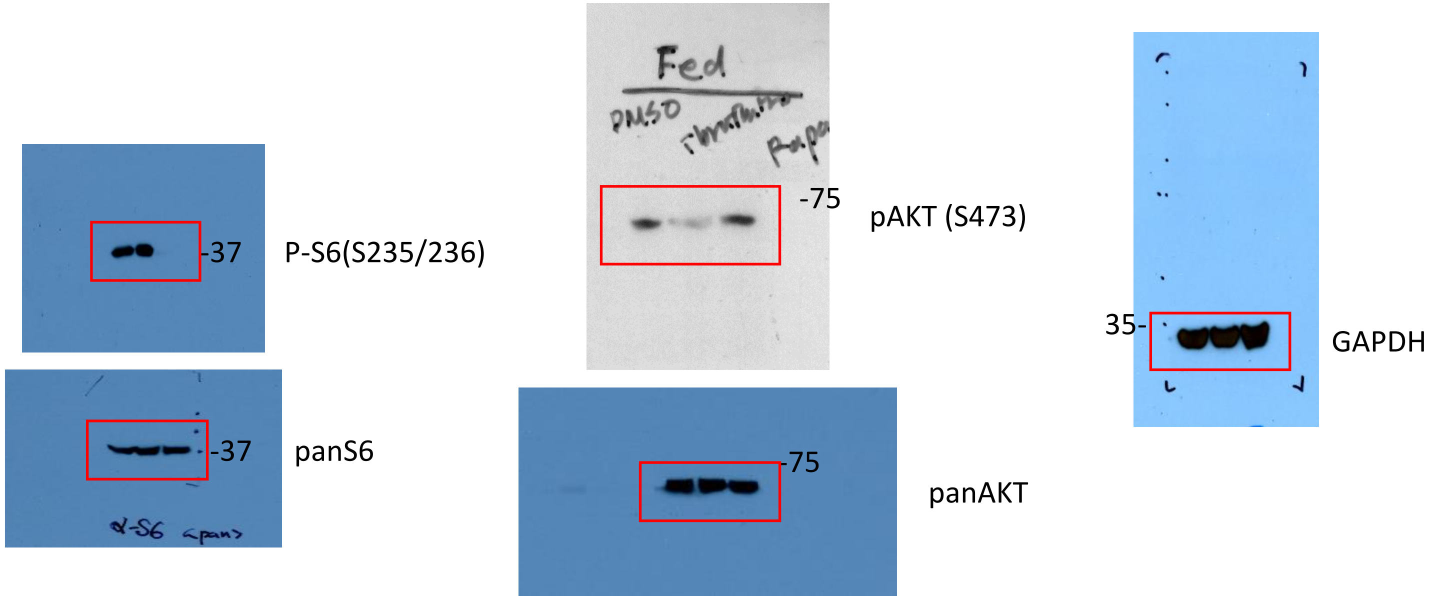
Fed
DMSO
-37
P-S6(S235/236)
-37
panS6
-75
pAKT (S473)
-75
panAKT
35-
GAPDH

Fig6C.

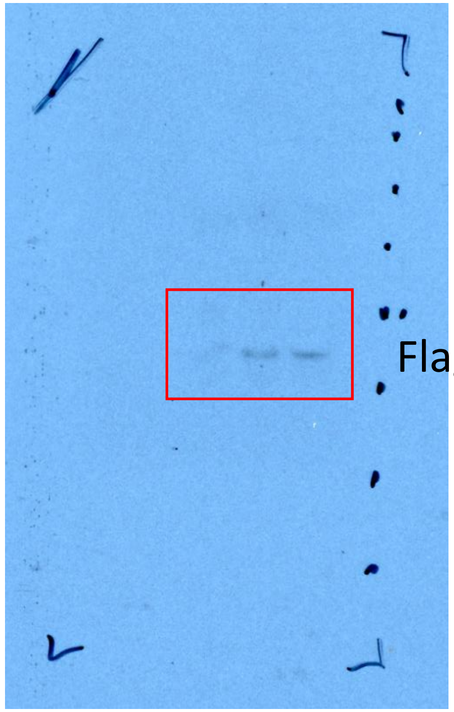

Flag-SRMS WT/K258A

Fig7A

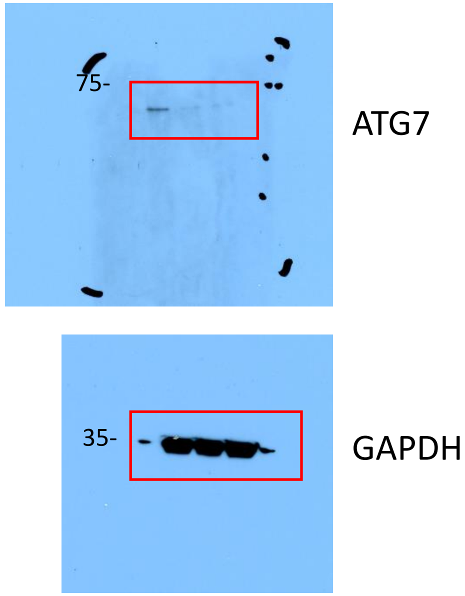

Fig7B

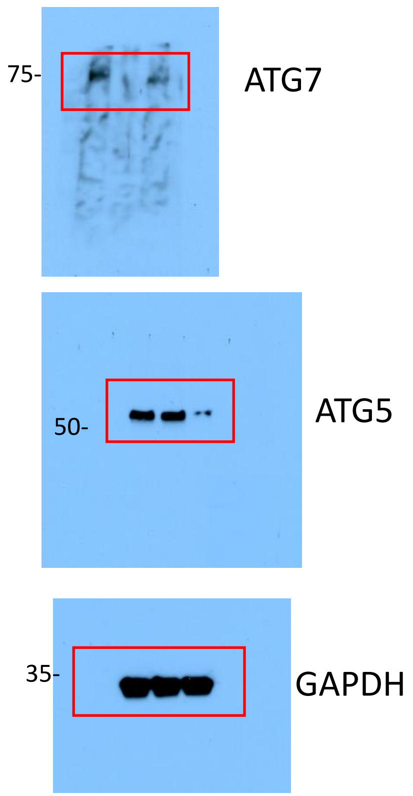

SFig1A

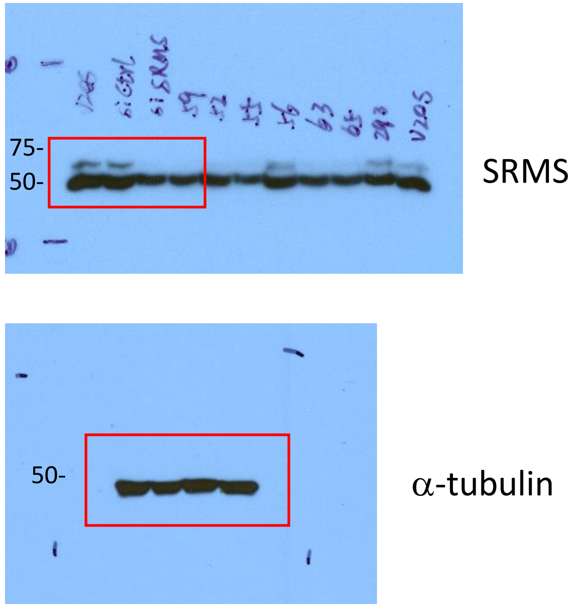

SFig1E

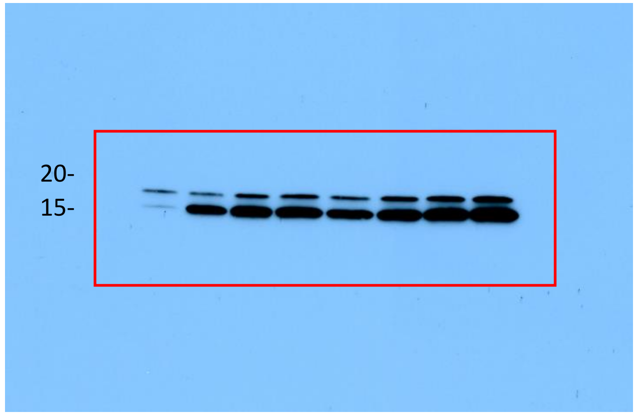

LC3 I/II

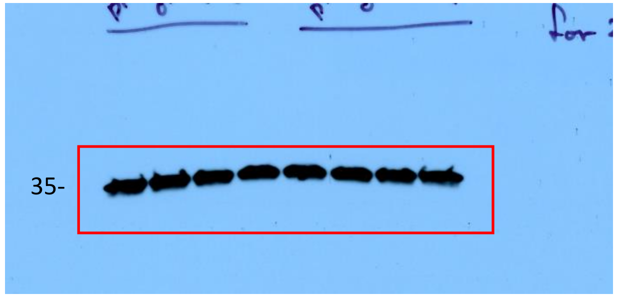

GAPDH

SFig1F

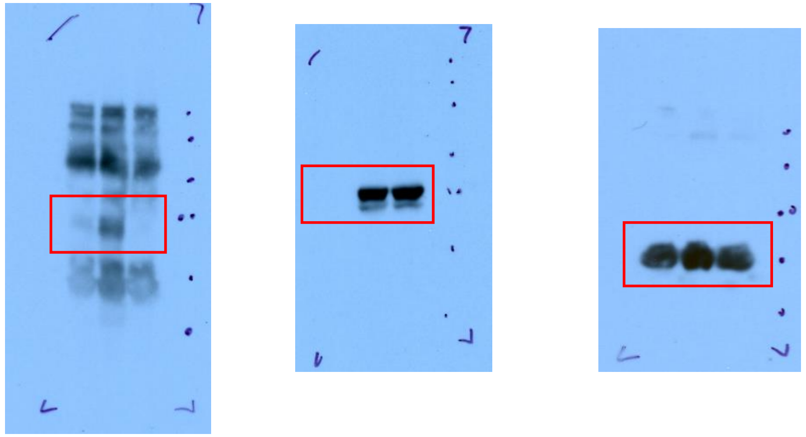

p-Tyr SRMS β-actin

SFig2A

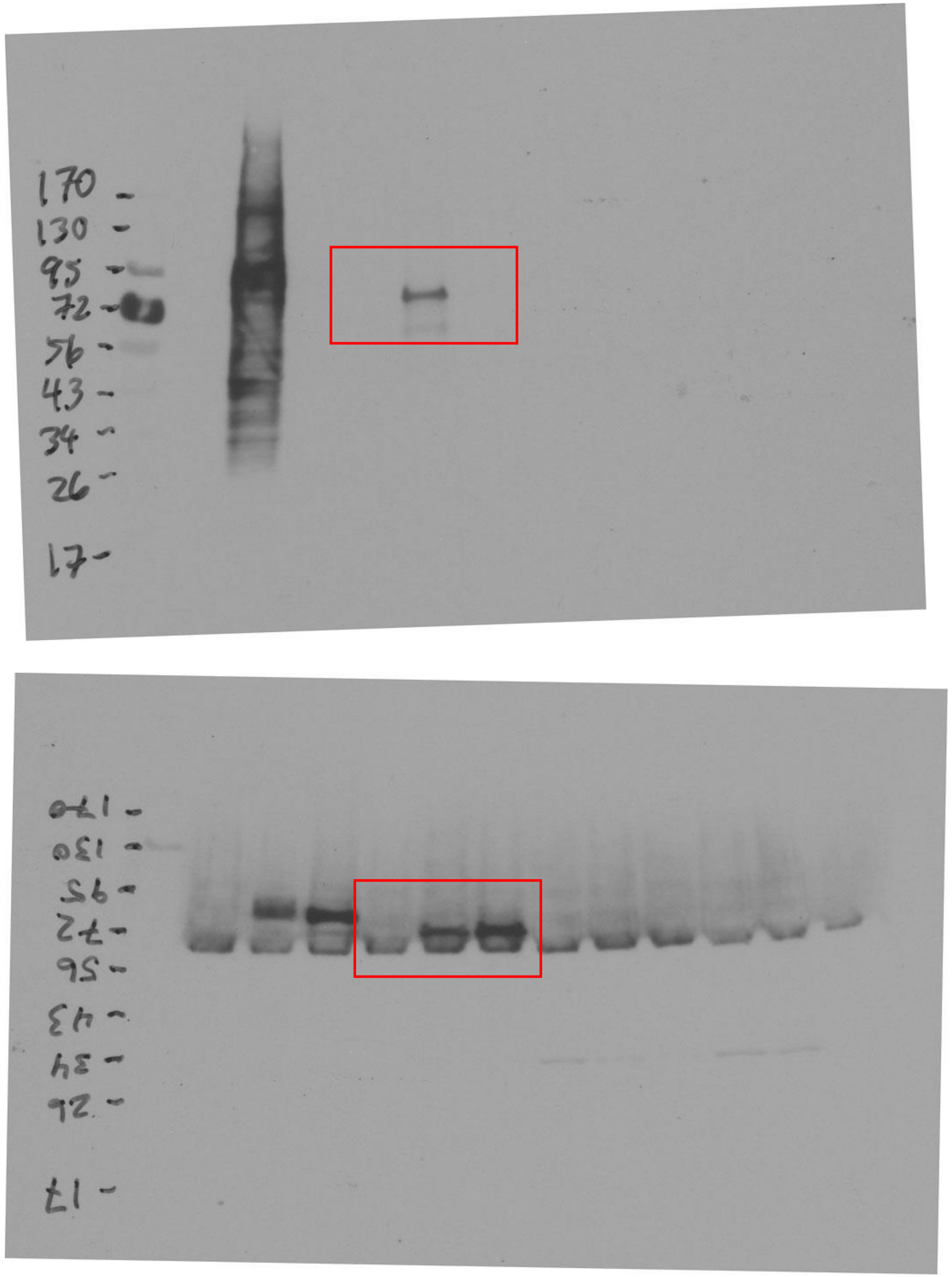

p-Tyr

SRMS

SFig2B

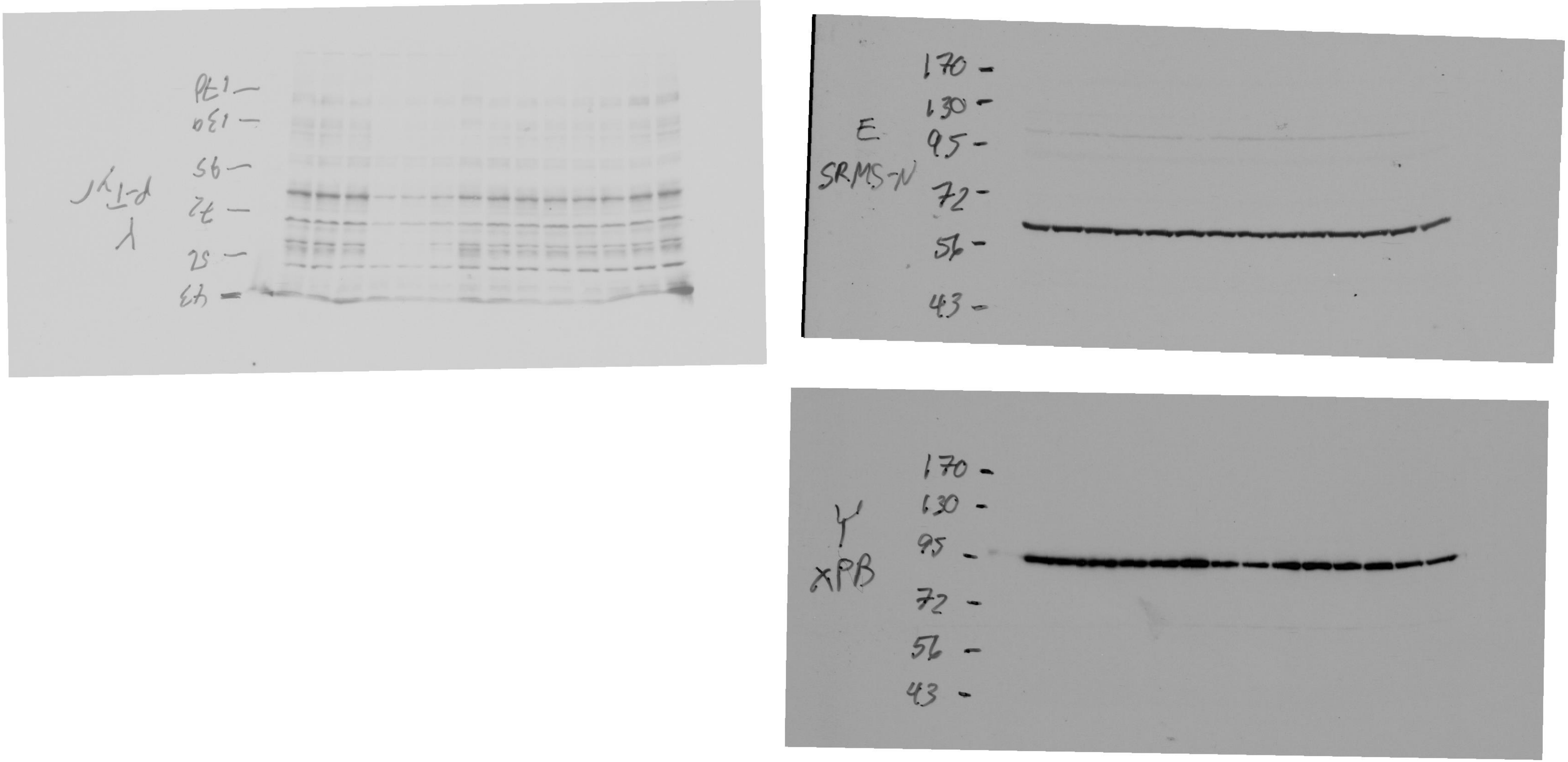

SFig2C

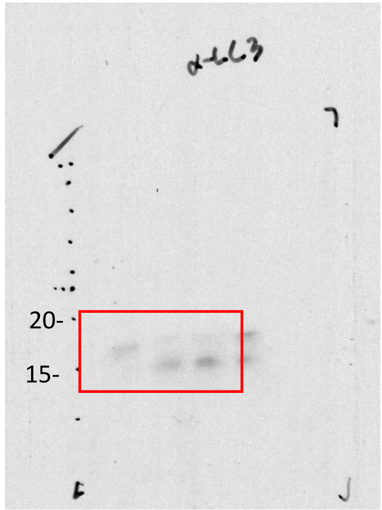

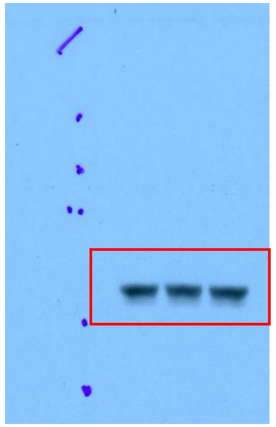

SFig2J

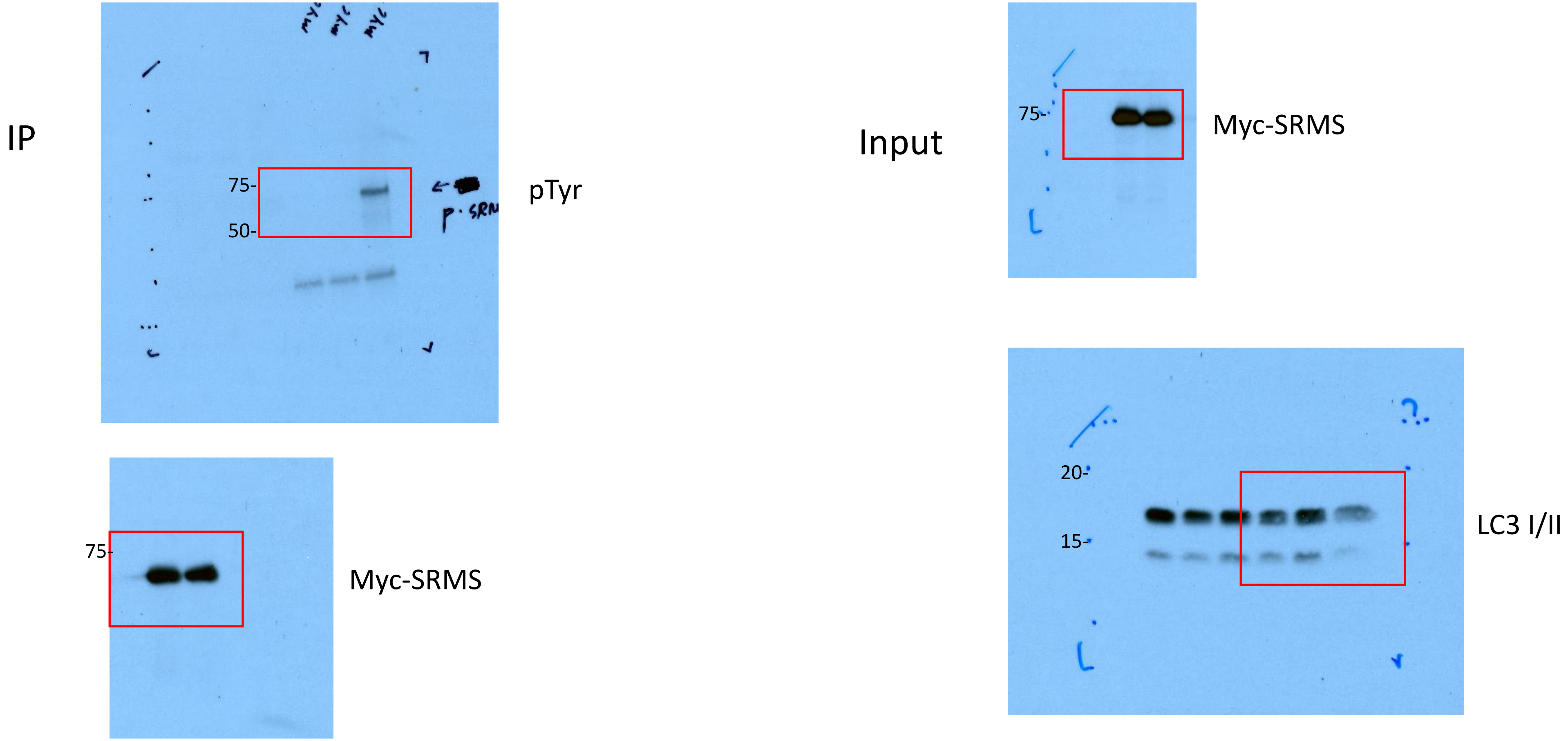

SFig3B

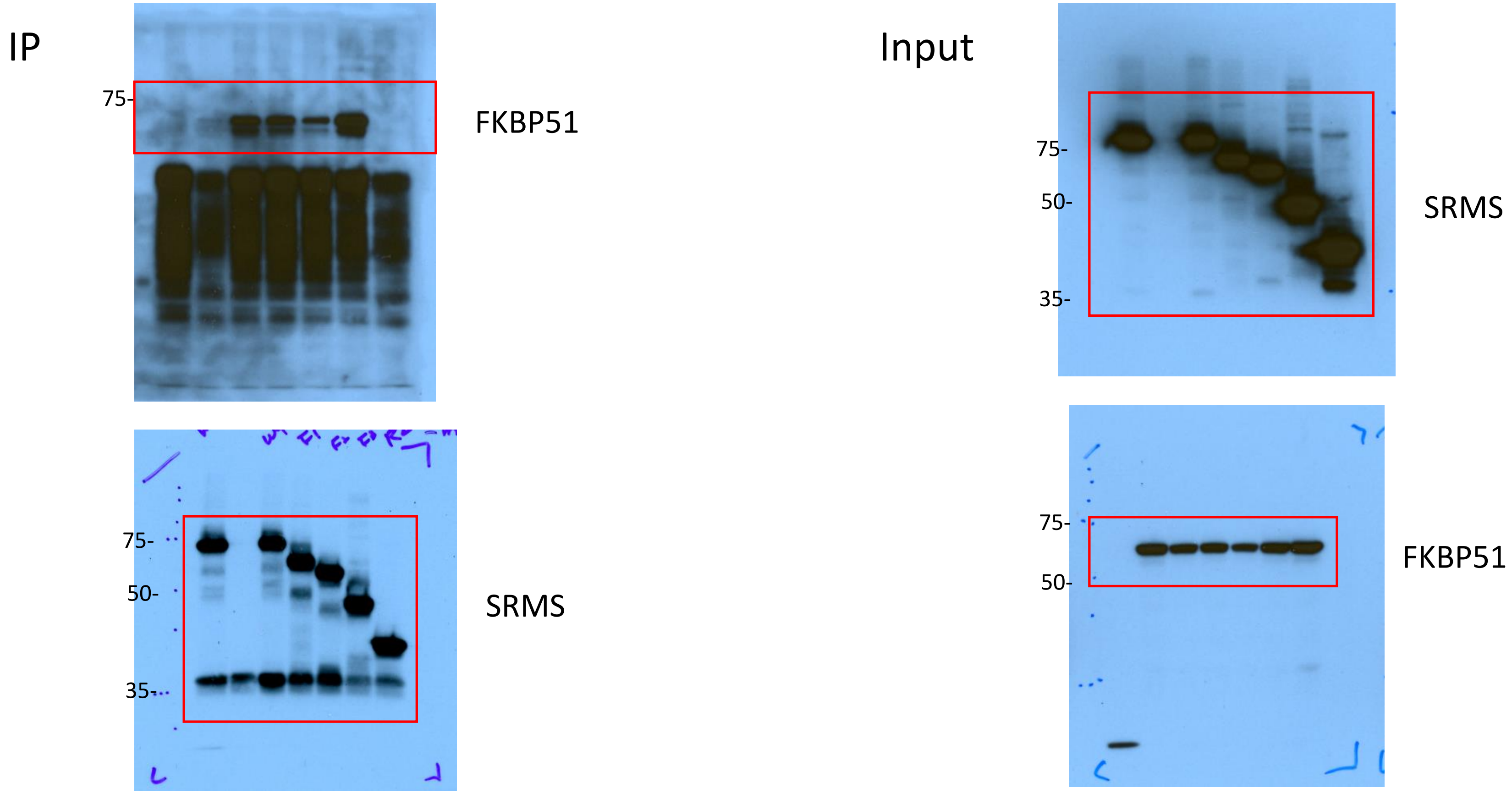

SFig3D

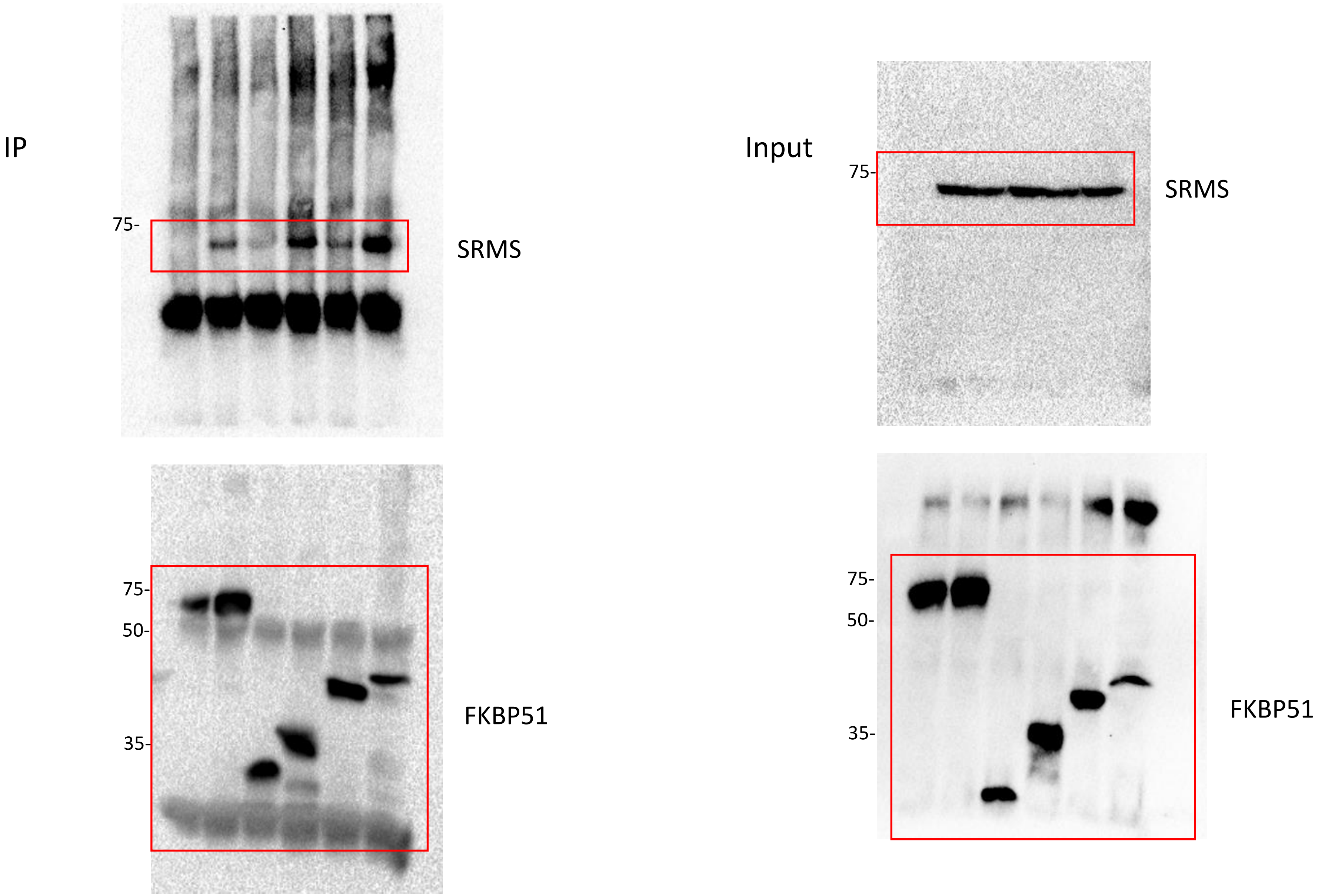

SFig3E

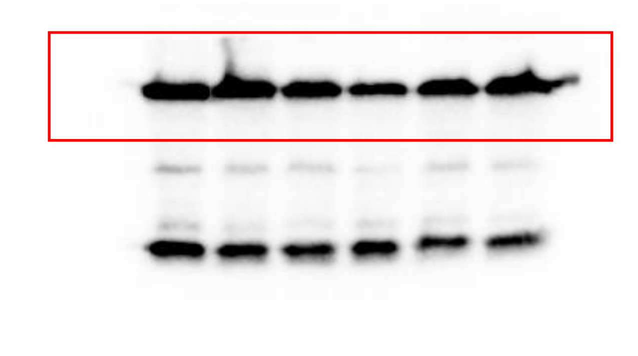

SRMS

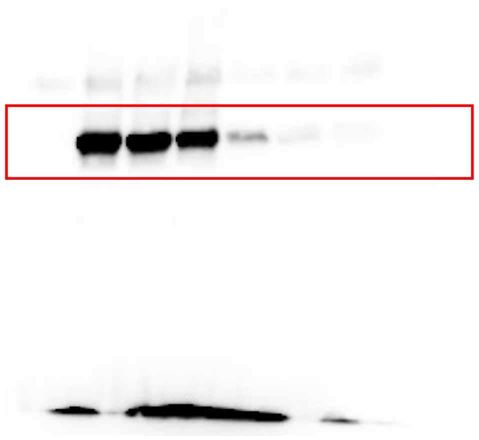

SRMS

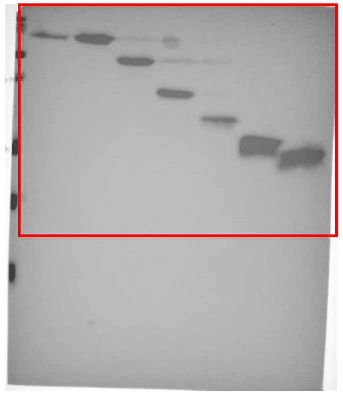

FKBP51

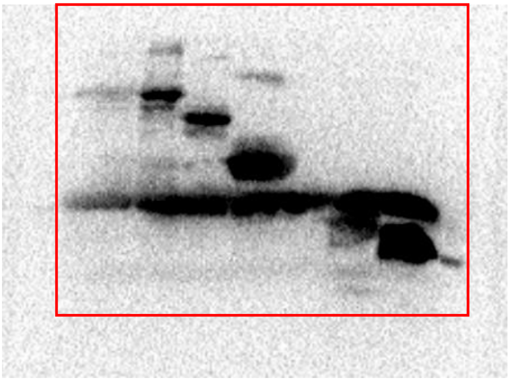

FKBP51

SFig3F

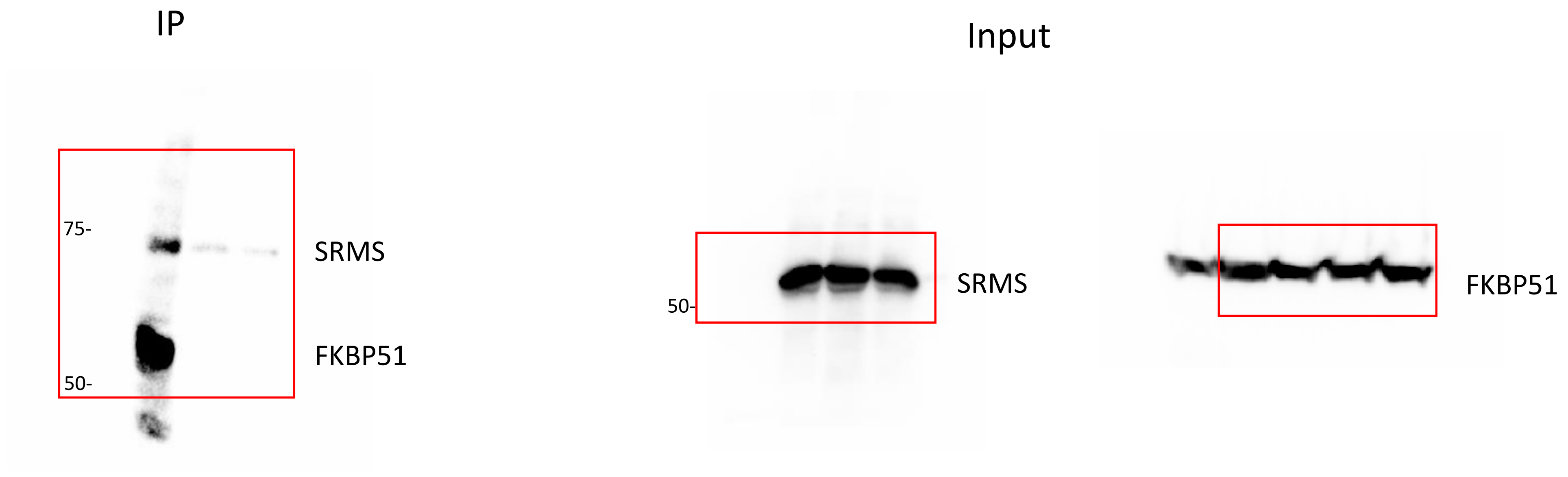

SFig4A

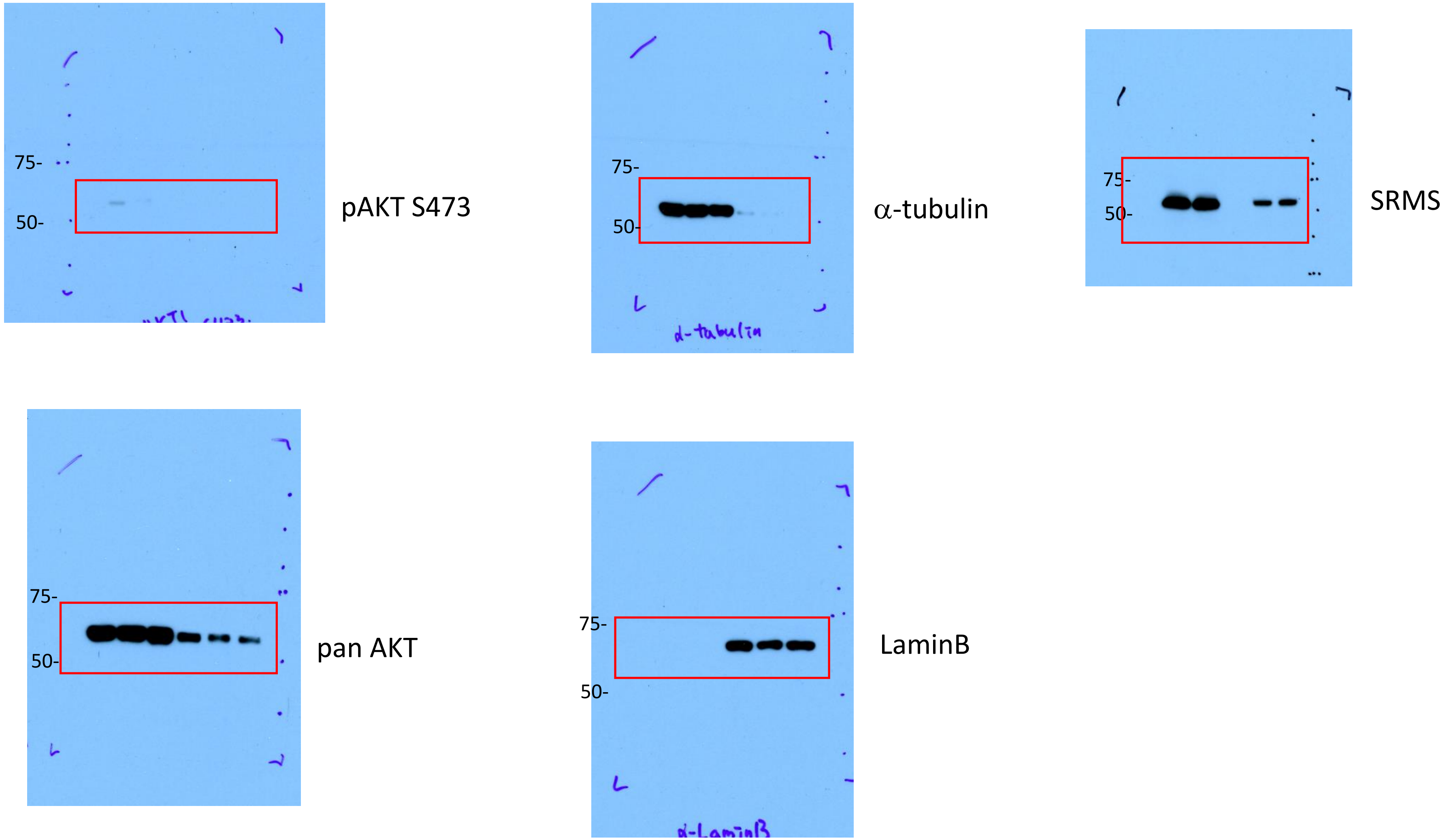
75-
50-
pAKT S473
75-
50-
α-tubulin
75-
50-
SRMS
75-
50-
pan AKT
75-
50-
LaminB

SFig4B

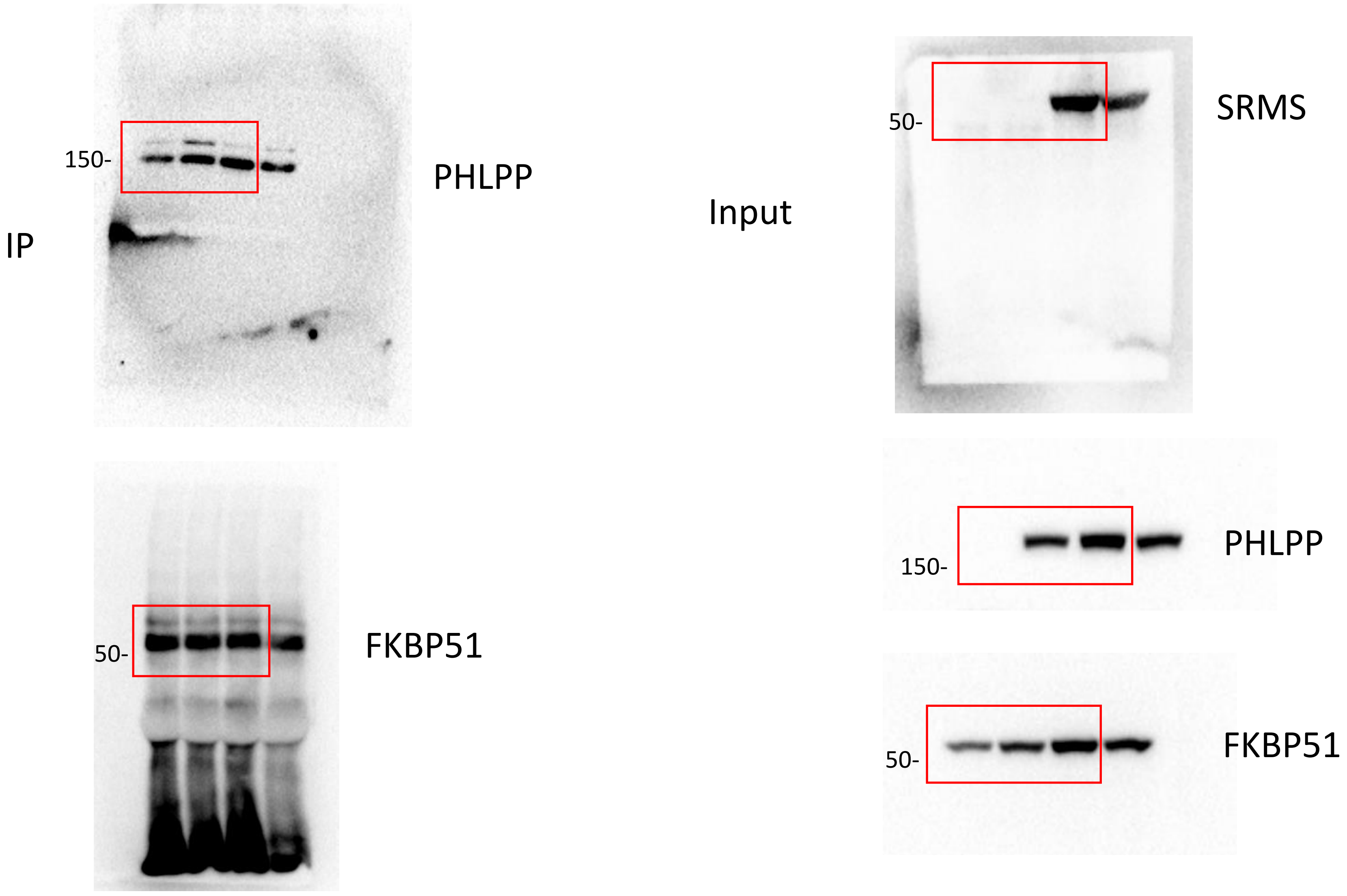

SFig4C

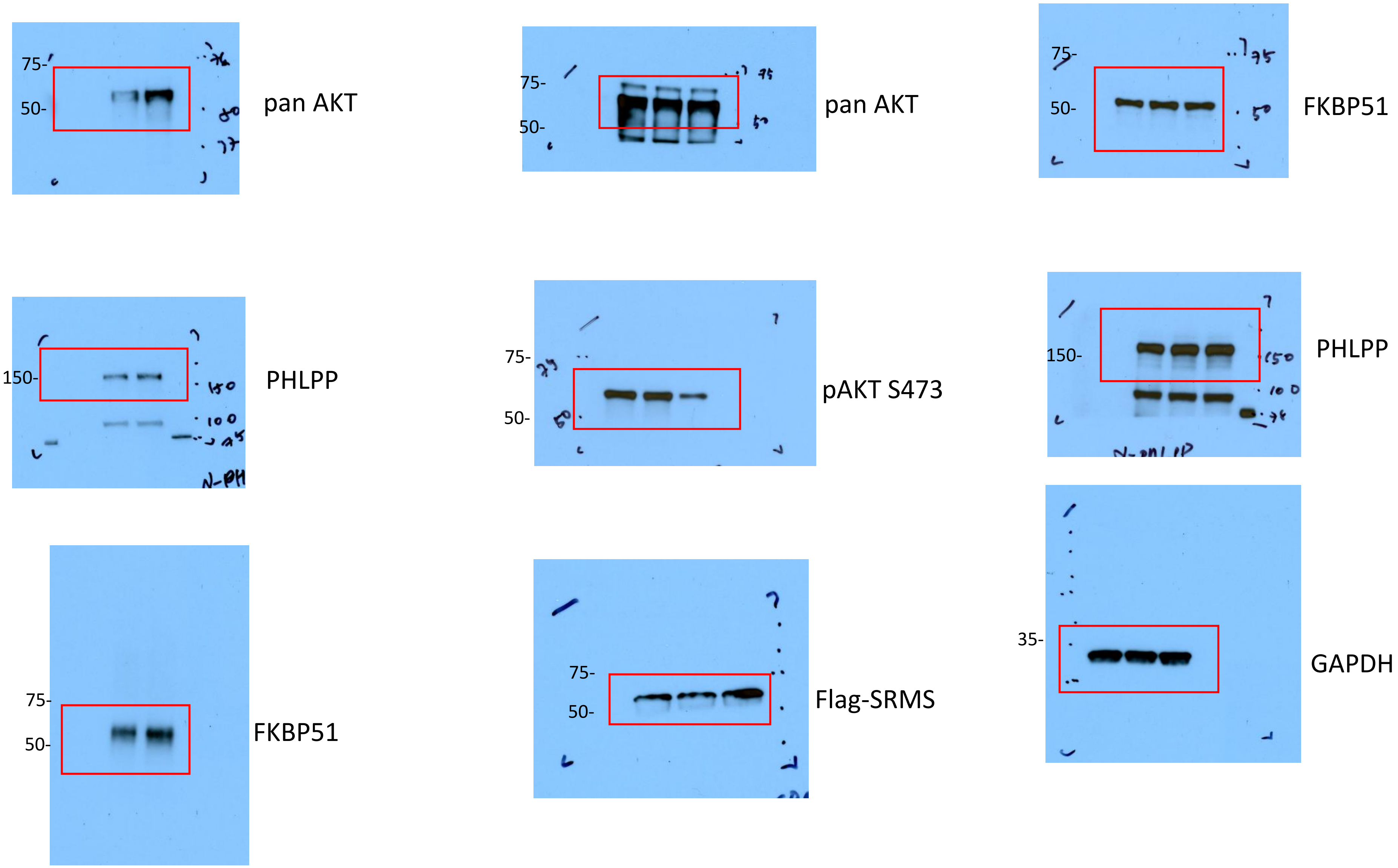

75-
50-
pan AKT
75-
50-
pan AKT
75-
50-
FKBP51
150-
PHLPP
75-
50-
pAKT S473
150-
PHLPP
75-
50-
FKBP51
75-
50-
Flag-SRMS
35-
GAPDH

SFig4D

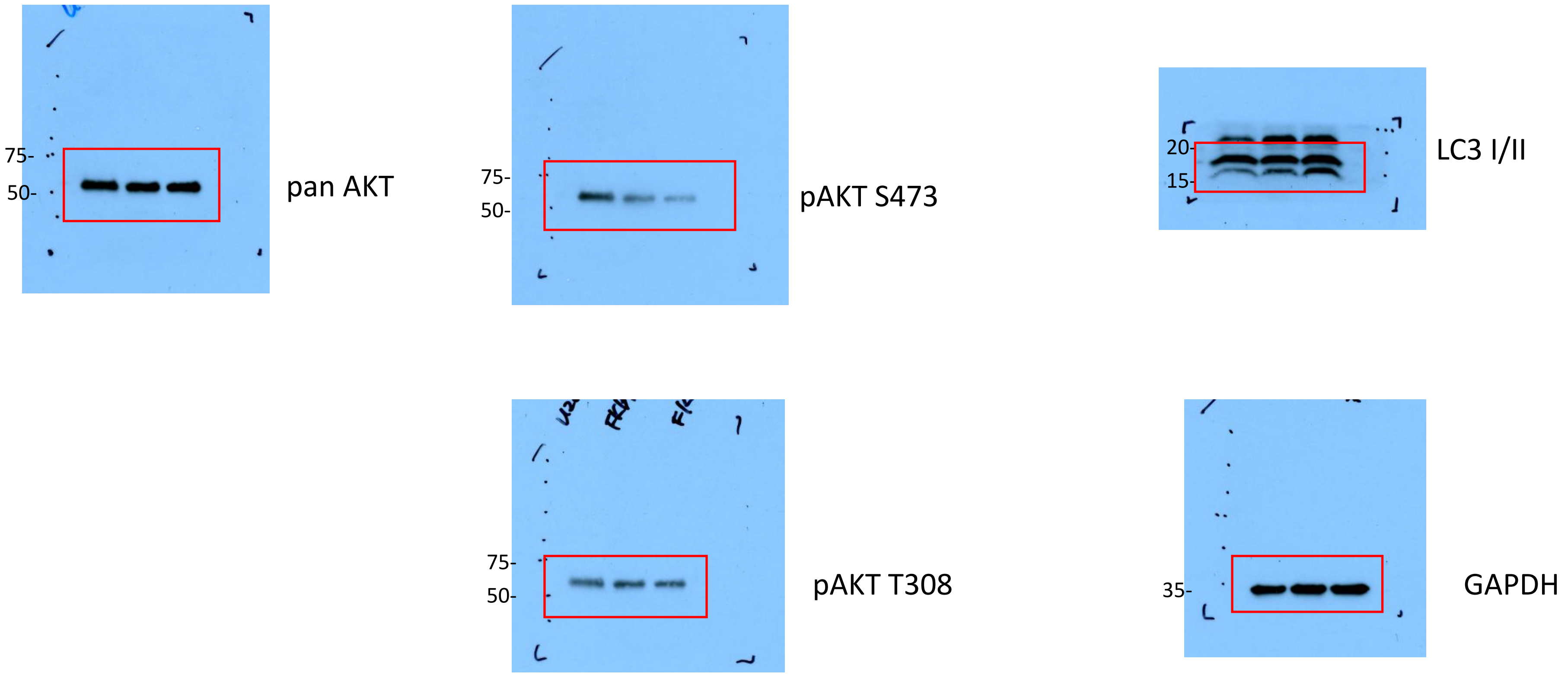
75-
50-
pan AKT
75-
50-
pAKT S473
20-
15-
LC3 I/II
75-
50-
pAKT T308
35-
GAPDH

SFig4F

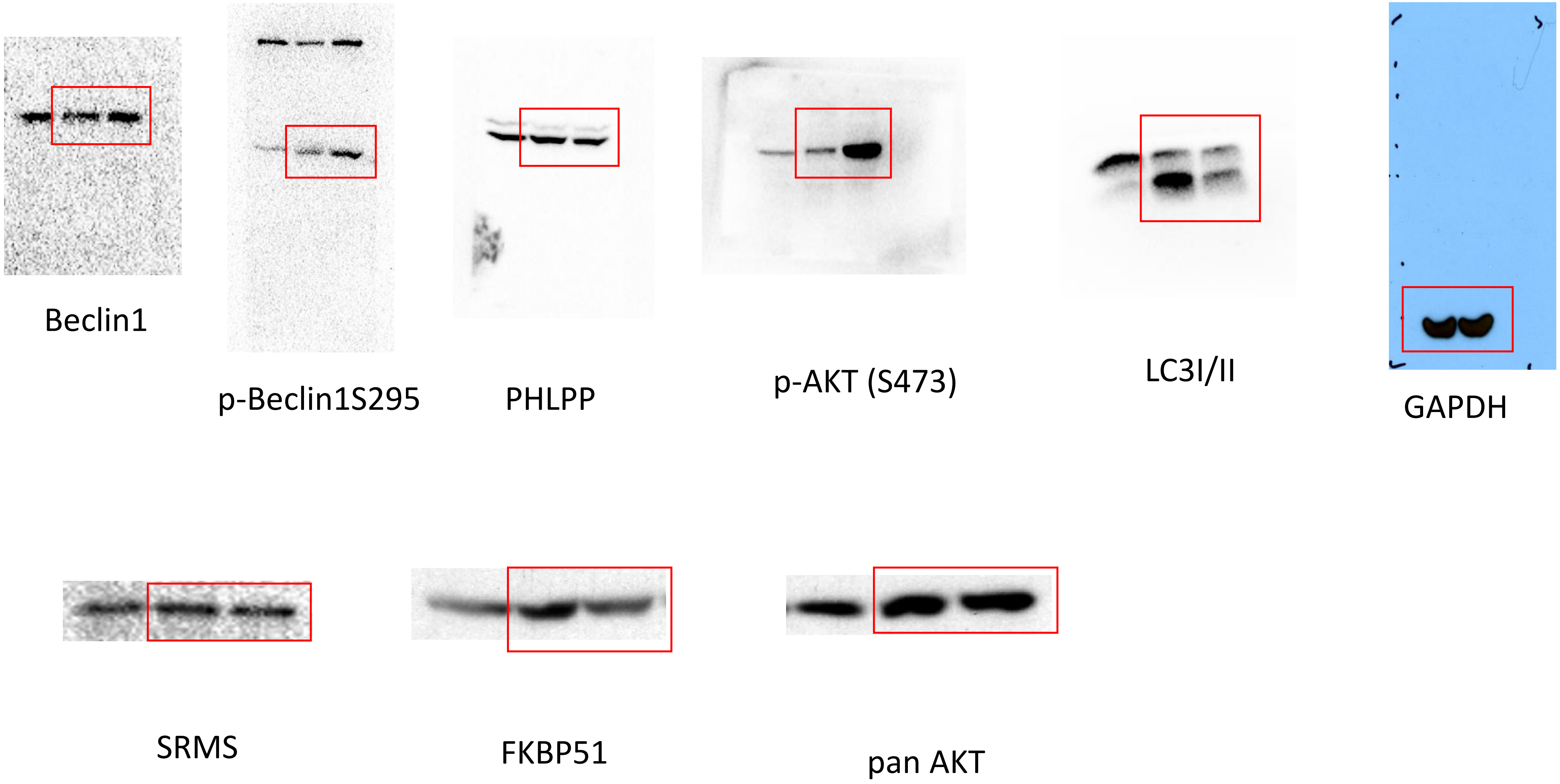
Beclin1
p-Beclin1S295
PHLPP
p-AKT (S473)
LC3I/II
GAPDH
SRMS
FKBP51
pan AKT

SFig4G

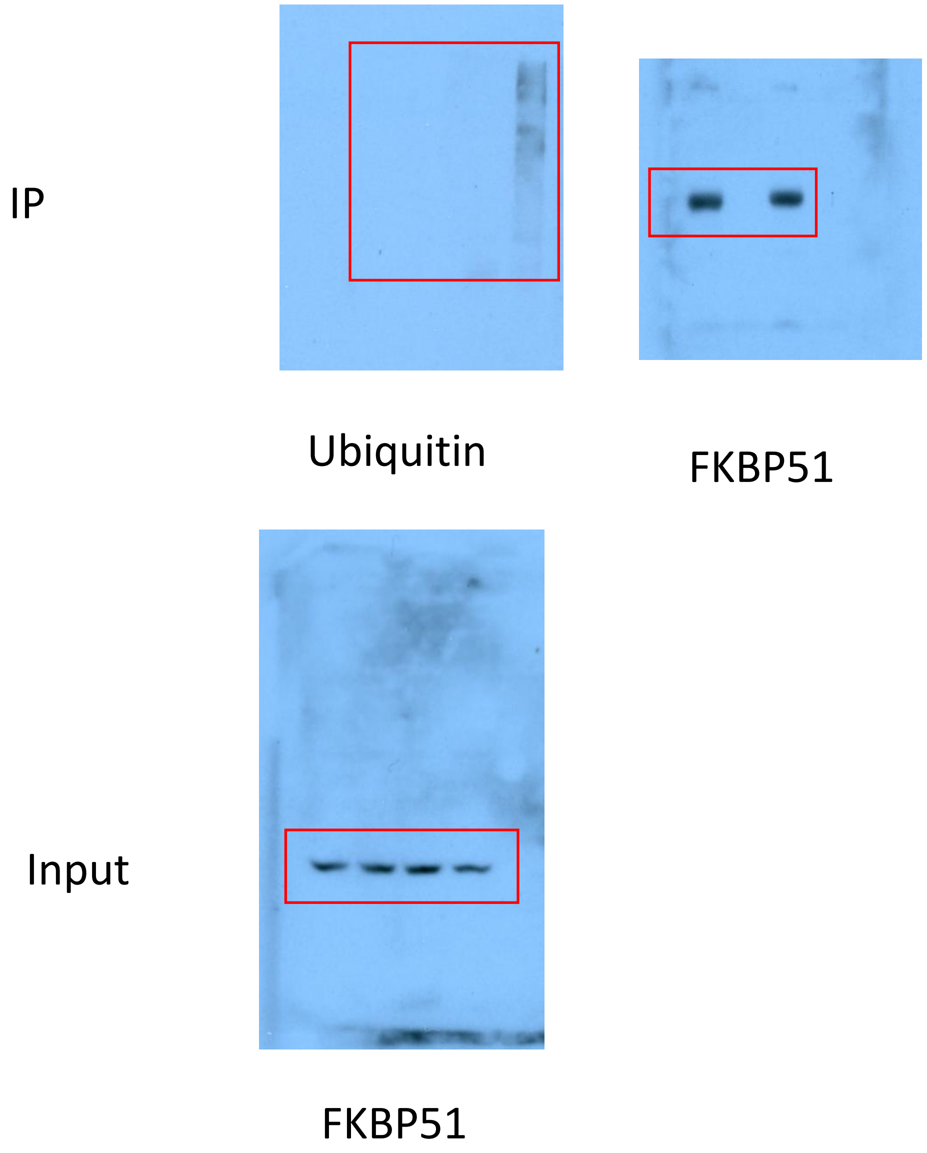

SFig4H

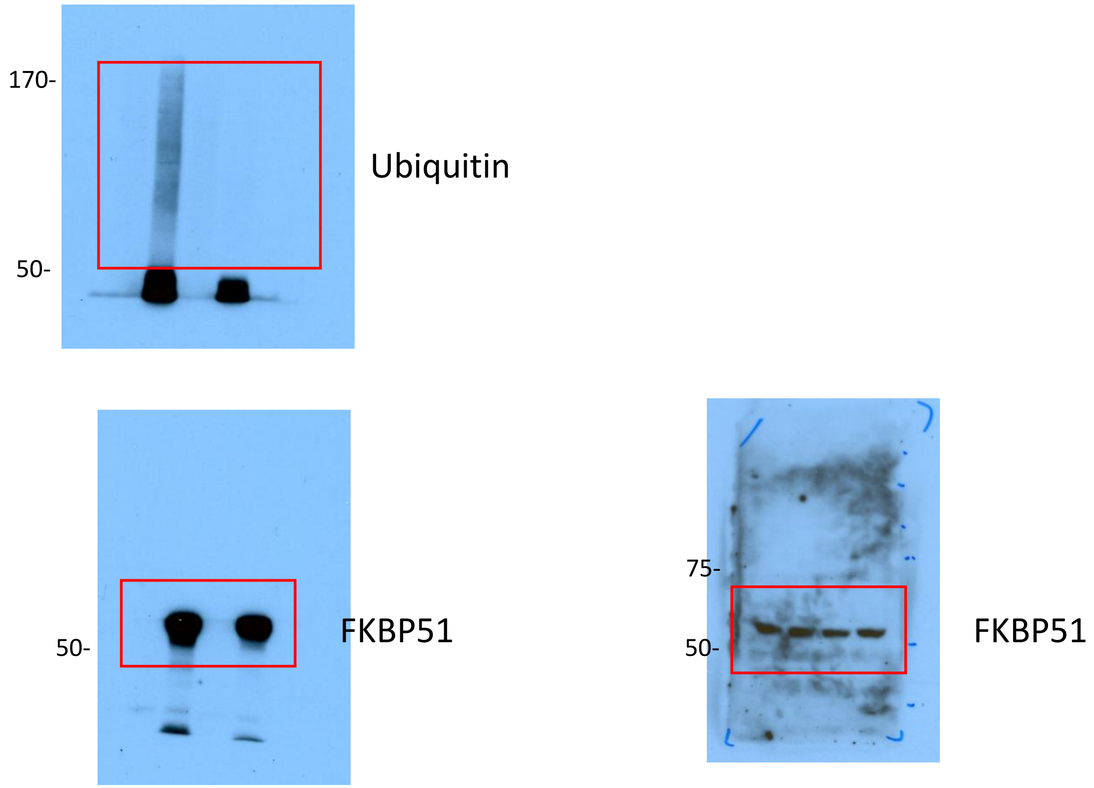

SFig4I

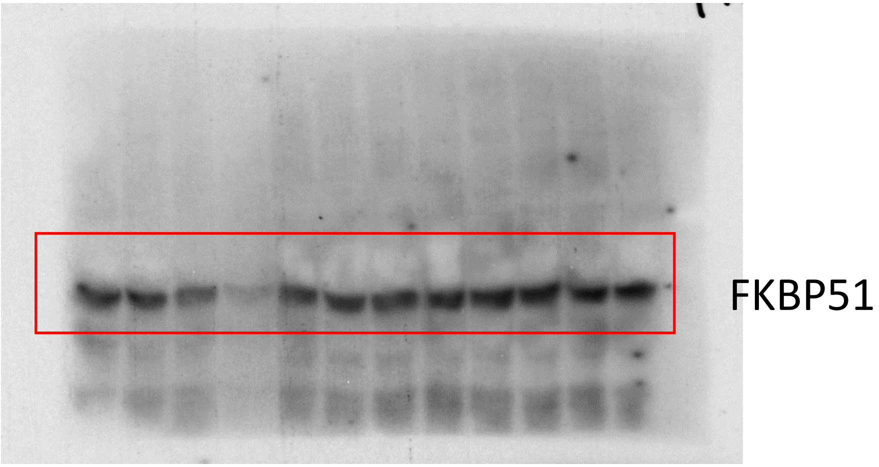

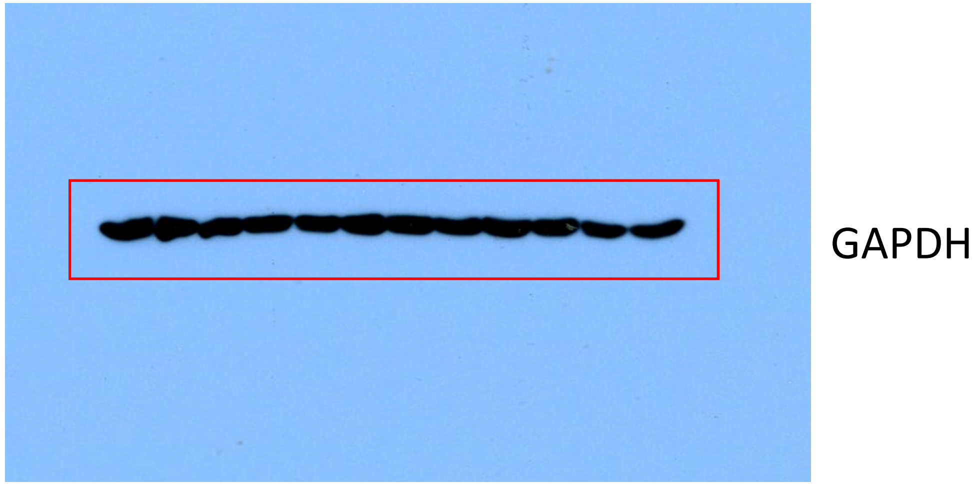

SFig5A

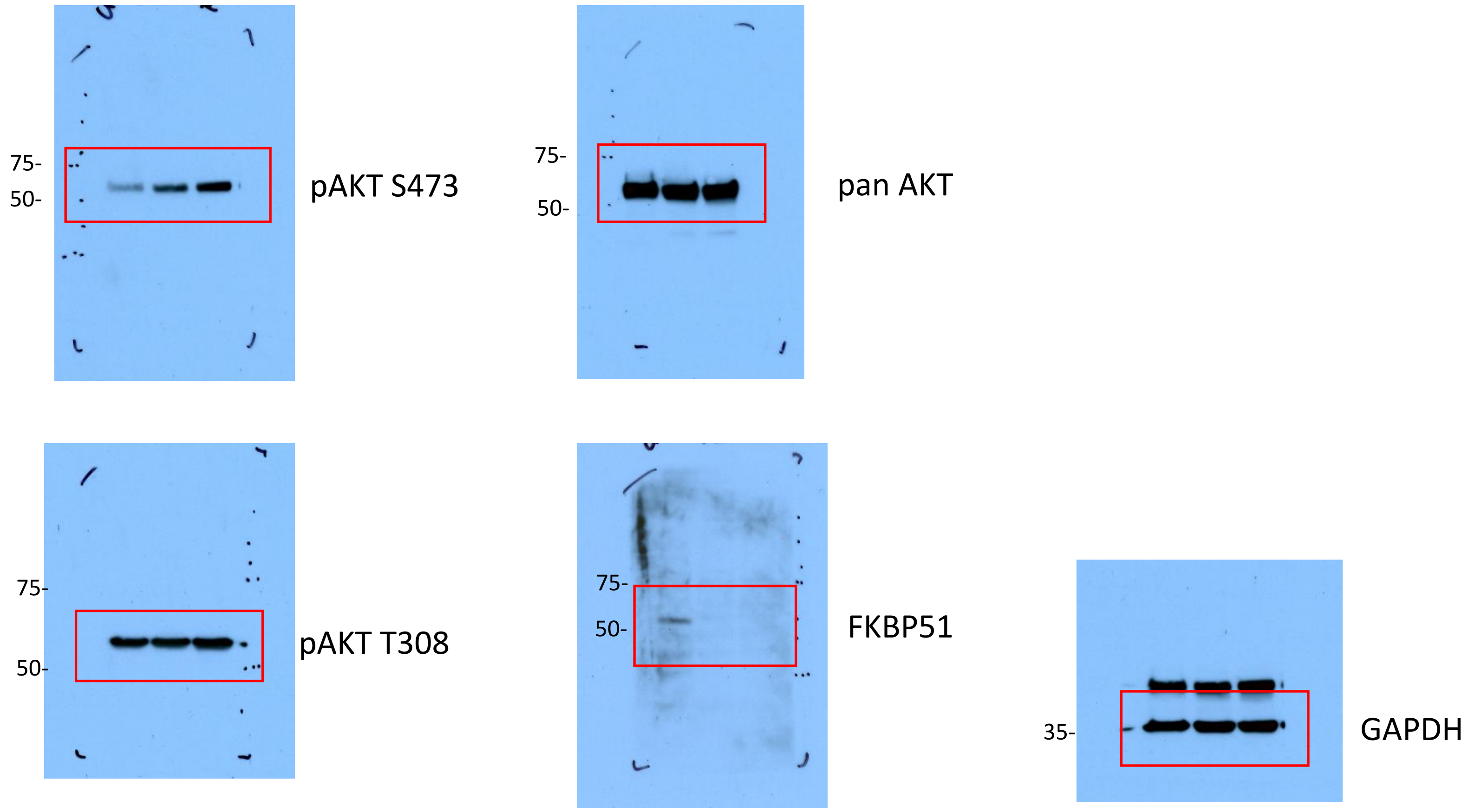
75-
50-
pAKT S473
75-
50-
pan AKT
75-
50-
pAKT T308
75-
50-
FKBP51
35-
GAPDH

SFig5C

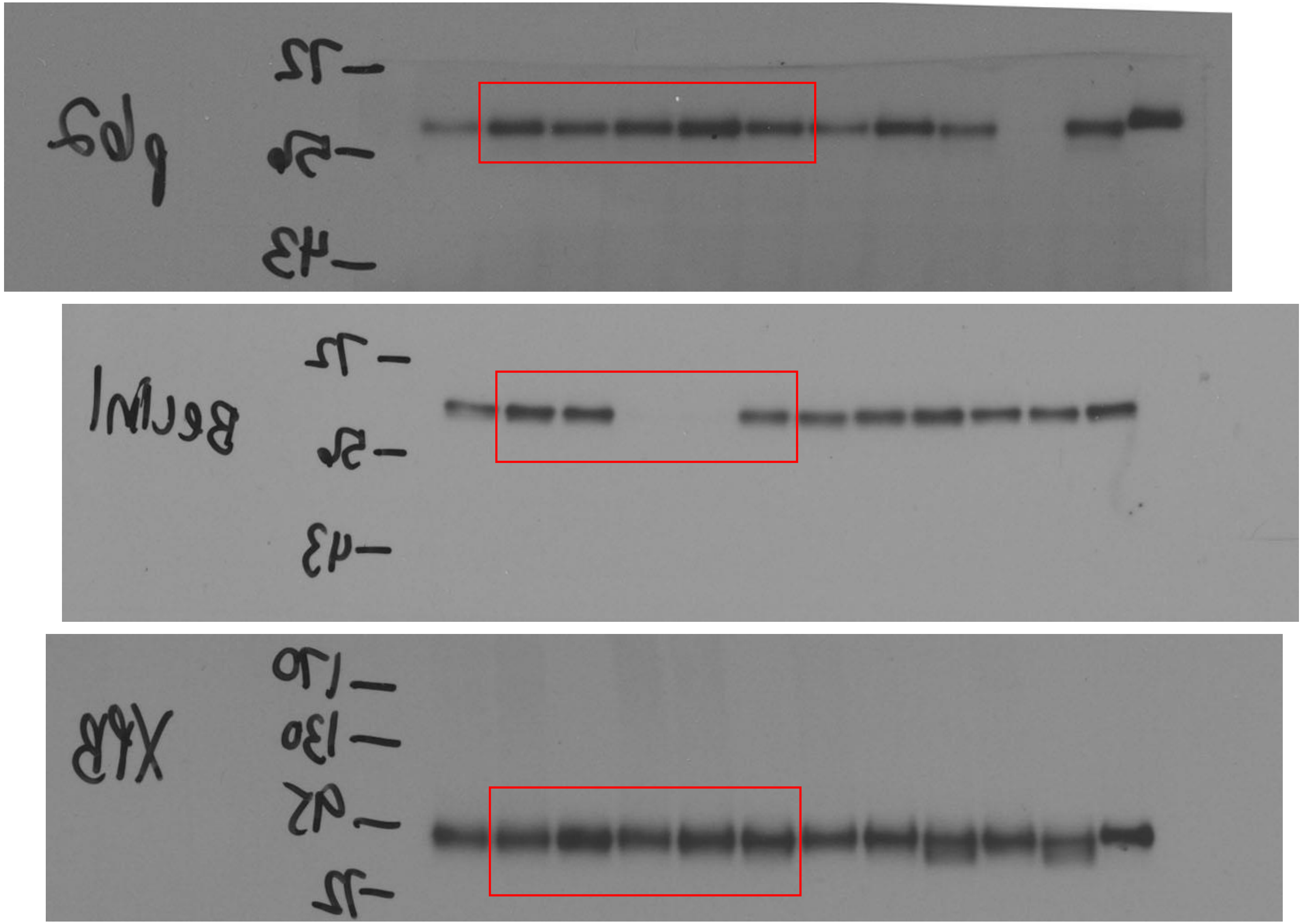

SFig5D

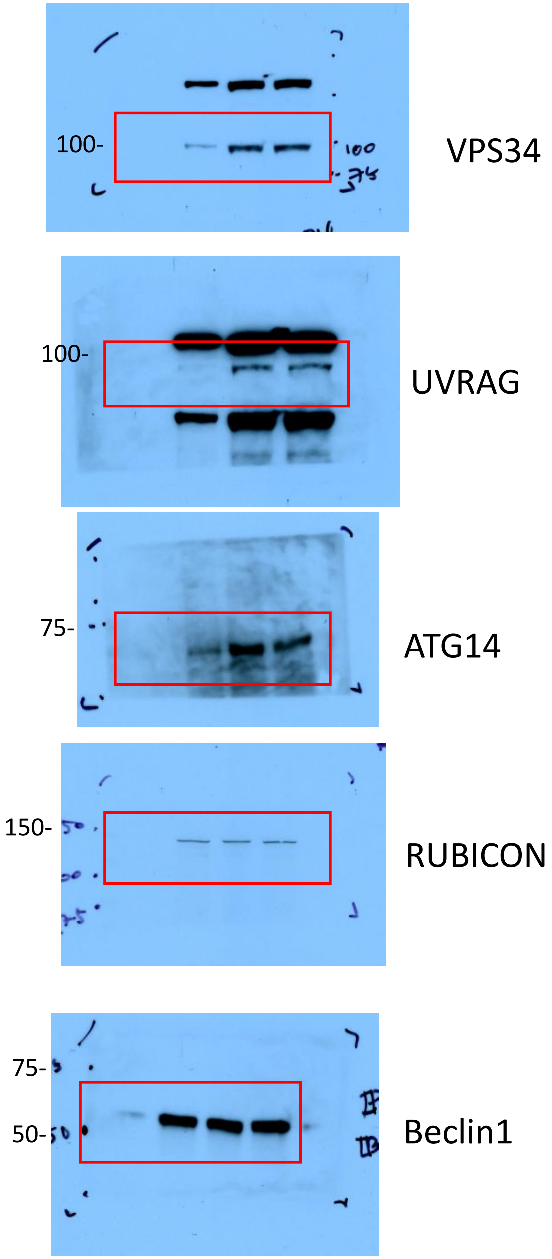
100-
VPS34
100-
UVRAG
75-
ATG14
150-
RUBICON
75-
50-
Beclin1

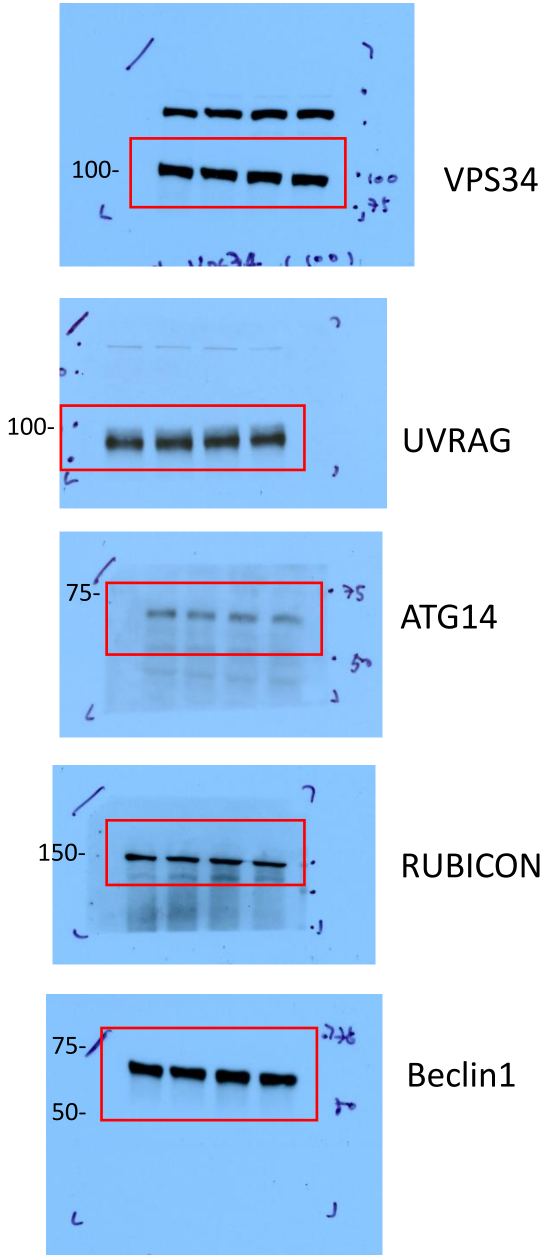
100-
VPS34
100-
UVRAG
75-
ATG14
150-
RUBICON
75-
50-
Beclin1

SFig5E

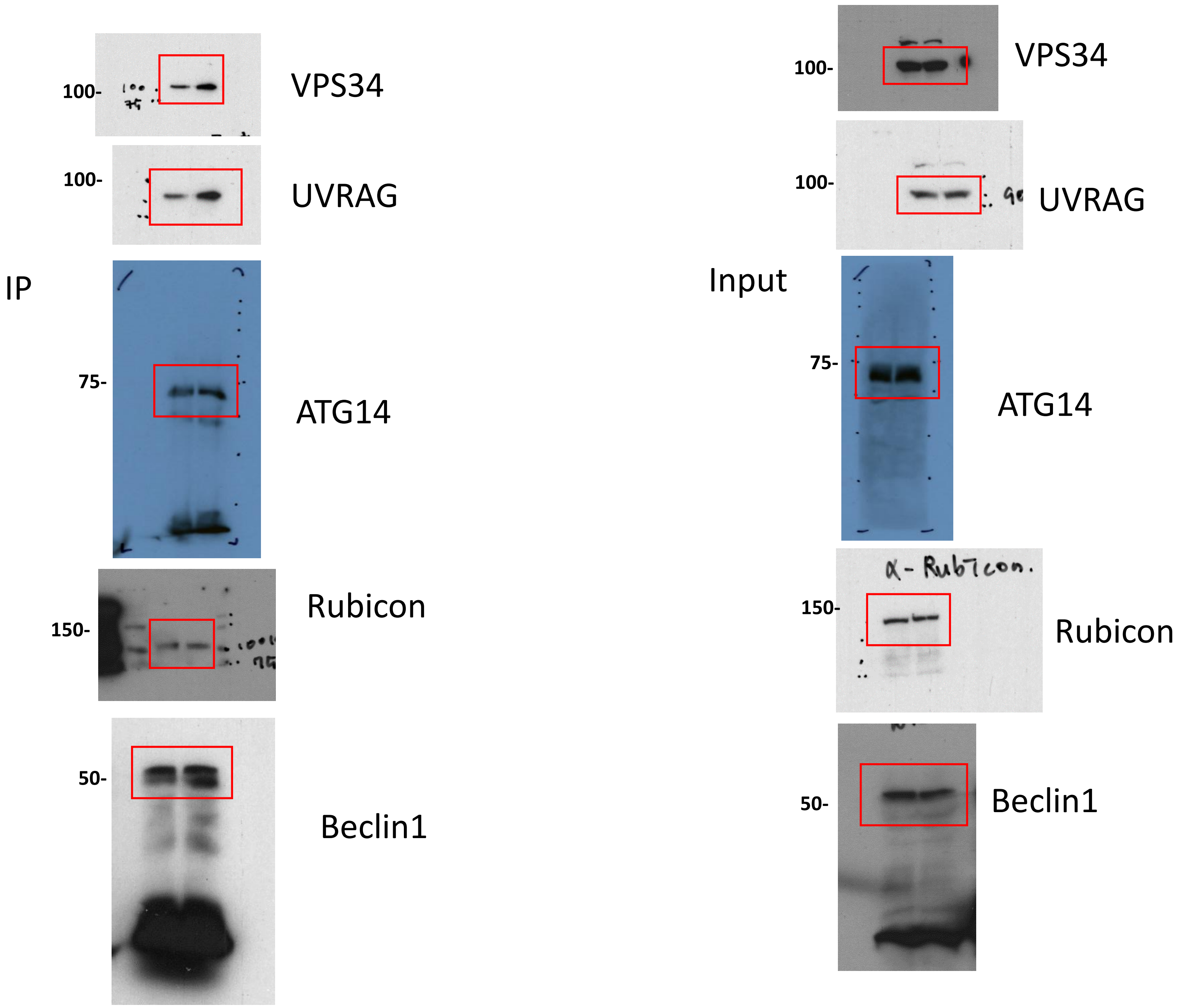
IP
100-
VPS34
100-
UVRAG
75-
ATG14
150-
Rubicon
50-
Beclin1
Input
100-
VPS34
100-
UVRAG
75-
ATG14
150-
Rubicon
50-
Beclin1

SFig6G

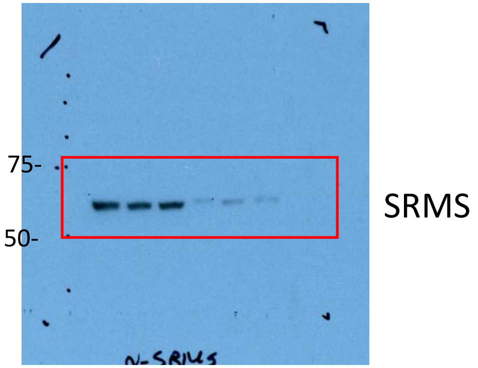

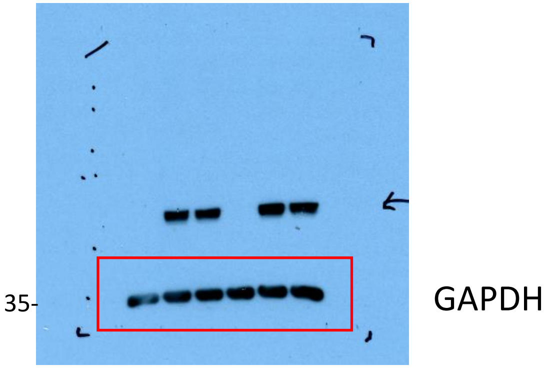

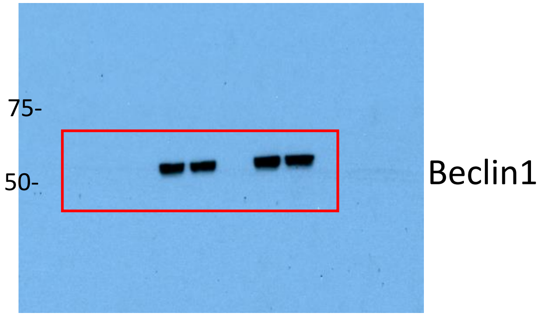

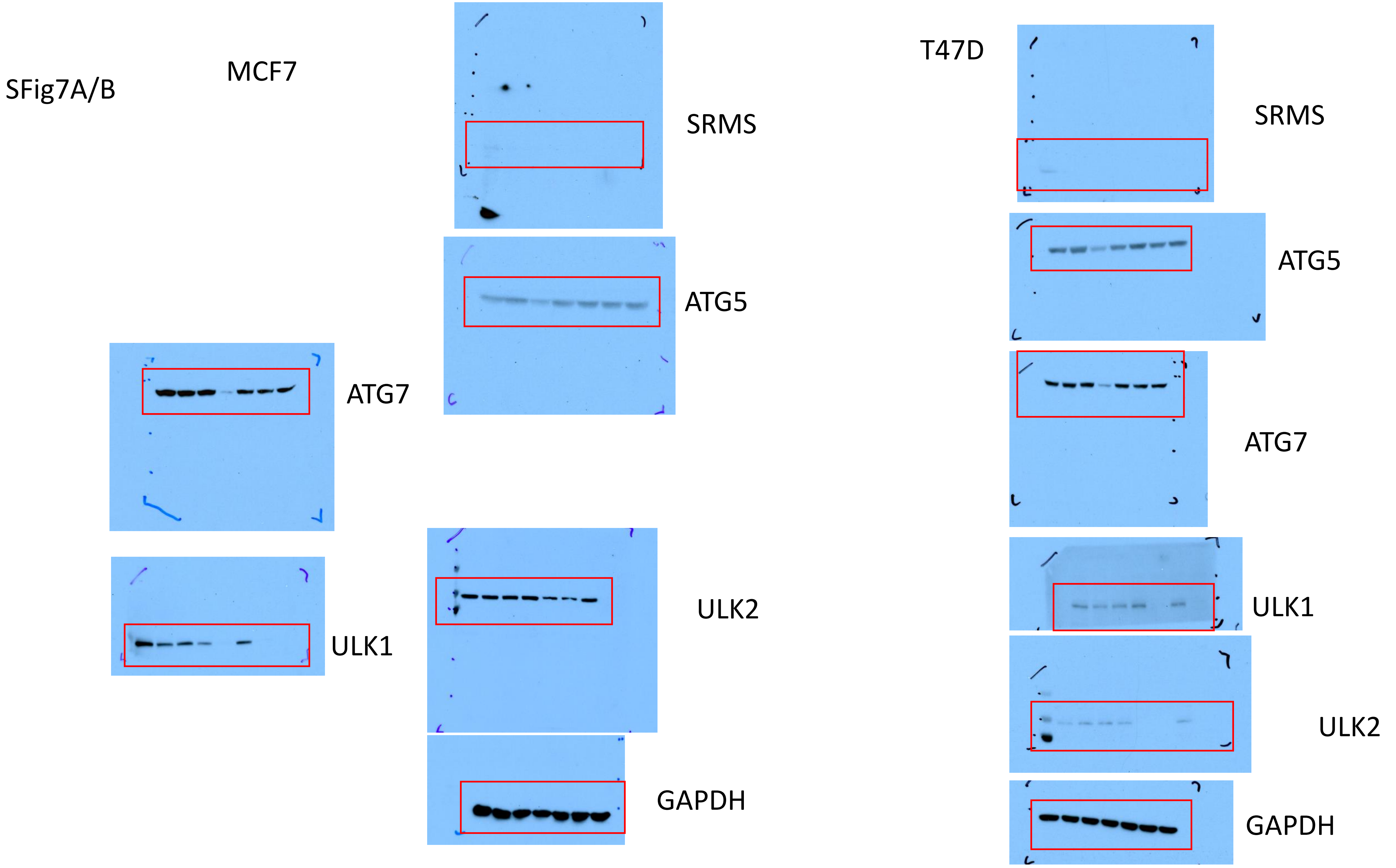
SFig7A/B
MCF7
SRMS
ATG5
ATG7
ULK1
ULK2
GAPDH
T47D
SRMS
ATG5
ATG7
ULK1
ULK2
GAPDH
